# Rapid Water Permeation by Aramid Foldamer Nanochannels with Hydrophobic Interiors

**DOI:** 10.1002/anie.202504170

**Published:** 2025-04-04

**Authors:** Saquib Farooq, Javid Ahmad Malla, Miroslava Nedyalkova, Rafael V. M. Freire, Indradip Mandal, Aurelien Crochet, Stefan Salentinig, Marco Lattuada, Charlie T. McTernan, Andreas F. M. Kilbinger

**Affiliations:** 1 Department of Chemistry, University of Fribourg, Chemin du Musée 9, CH-1700 Fribourg, Switzerland; 2 Artificial Molecular Machinery Laboratory, The Francis Crick Institute, 1 Midland Road, London, NW1 1AT, UK & Department of Chemistry, Britannia House, 7 Trinity Street, King’s College London, SE1 1DB, UK

**Keywords:** helical polymers, macrocycles, aramids, polymerization, foldamers, water transport, synthetic aquaporins, supramolecular, ion selectivity

## Abstract

Aquaporins are natural proteins that rapidly transport water across cell membranes, maintaining homeostasis, whilst strictly excluding salt. This has inspired their use in water purification and desalination, a critical emerging need. However, stability, scalability, and cost have prevented their widespread adoption in water purification membrane technologies. As such, attention has turned to the use of artificial water channels, with pore functionalized polymers and macrocycles providing a powerful alternative. Whilst impressive rates of transport have been achieved, the combination of a scalable, high-yielding synthesis and efficient transport has not yet been reported. Herein, we report such a system, with densely functionalized channel interiors, synthesized by high-yielding living polymerization with low polydispersities, showing high salt exclusion, and excellent water transport rates. Our aramid foldamers create artificial water channels with hydrophobic interiors, and single-channel water permeability rates of up to 10^8^ water molecules per second per channel, approaching the range of natural Aquaporins (c. 10^9^). We show that water transport rates closely correspond to the helical length, with the polymer which most closely matches bilayer thickness showing optimal efficacy, as supported by molecular dynamics simulations. Our work provides a basis for the scalable synthesis of next-generation artificial water channels.

## Graphical abstract

**Graphical Abstract:**
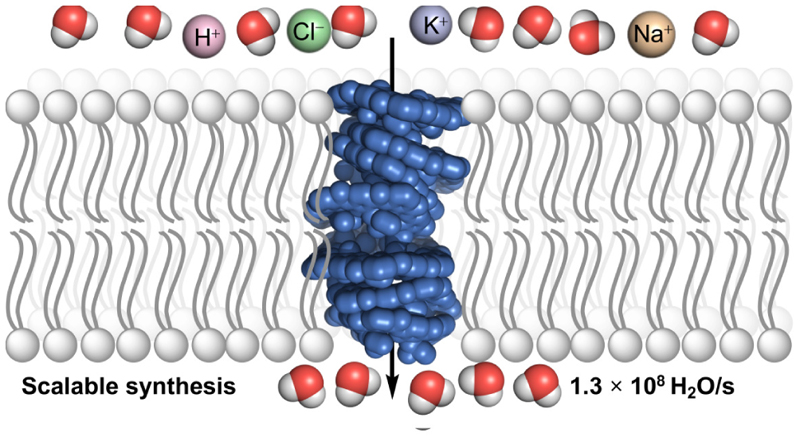
We compare the water permeability of stacked macrocycles and synthetic helical nanotubes of three different lengths across phospholipid bilayers. Our results demonstrate that covalent nanotubes with a length matching the bilayer width exhibit high water permeability, excellent salt and proton rejection, and performance comparable to natural aquaporins.

## Introduction

Biological water channels, such as the aquaporins, efficiently transport water across cell membranes while excluding ions.1 The Percec group was among the pioneers synthesizing aquaporin mimics based on dendritic dipeptides that allowed robust self-assembly into helical porous nanotubes in solution and in bulk.^2–5^ Inspired by aquaporin’s performance, researchers have developed a range of aquaporin- based biomimetic membranes for water purification and desalination.^6,7^ However, protein-based membranes have several limitations, including high production costs, challenges with scalability, and low stability.^8^ To overcome these limitations, the field has turned to artificial water channels.^9–27^

Artificial water channels are synthetic analogs of aquaporins with simpler structures, but which retain water transport and ion exclusion capabilities.^9^ Inspired by synthetic successes in mimicking biological ion channels^28–32^ first-generation artificial water channels, such as imidazole-quartets,^33^ and hydrazide pillars,^34^ with pore sizes ranging from 3 to 5 Å, focused on mimicking the one-dimensional water permeation route of aquaporins by constructing tubular transmembrane nanostructures. However, these channels have extensive hydrogen bond interactions between the pore walls and diffusing water wires, resulting in lower permeabilities.^34^

The idea of minimizing pore-wall/water interactions inspired the next generation of channels, with researchers incorporating hydrophobic pore-lining groups.^35,36^ This approach effectively minimizes water- pore wall interactions, leading to high water permeability.^37^ Several approaches have been successful, with carbon nanotubes showing high rates of water transport, but poor ion selectivity due to cation- π interactions promoting ion passage, resulting in a loss of salt exclusion.^36,37^

To achieve both high ion selectivity and ultrafast water permeation, Zeng and coworkers developed foldamers and helical polymers with inner pores decorated with aliphatic hydrocarbons (i.e. methyl), creating a slightly positive electrostatic surface that excludes cations.^36,38^ Recently, Aida and co-workers demonstrated that incorporating hydrophobic and non-polarizable fluorine atoms in the pore enables ultrafast water permeation, with rates surpassing those of natural aquaporins.^36,39,40^ The key weakness of these approaches lies in the difficulty of synthesizing suitable molecules in sufficient yields (for example, the fluorinated macrocycles were recovered in <0.1% yield).^39^

Whilst polymers can be synthesized in higher yields, control over size distribution and reproducibility remain significant challenges, with existing examples synthesized via step-growth polymerization.^38^

This will make application of these breakthroughs challenging. Further, as water transport properties are highly dependent on average molecular weight and dispersity, gaining control of polymerization with a high yielding reaction is essential.^35^

**Figure 1:**
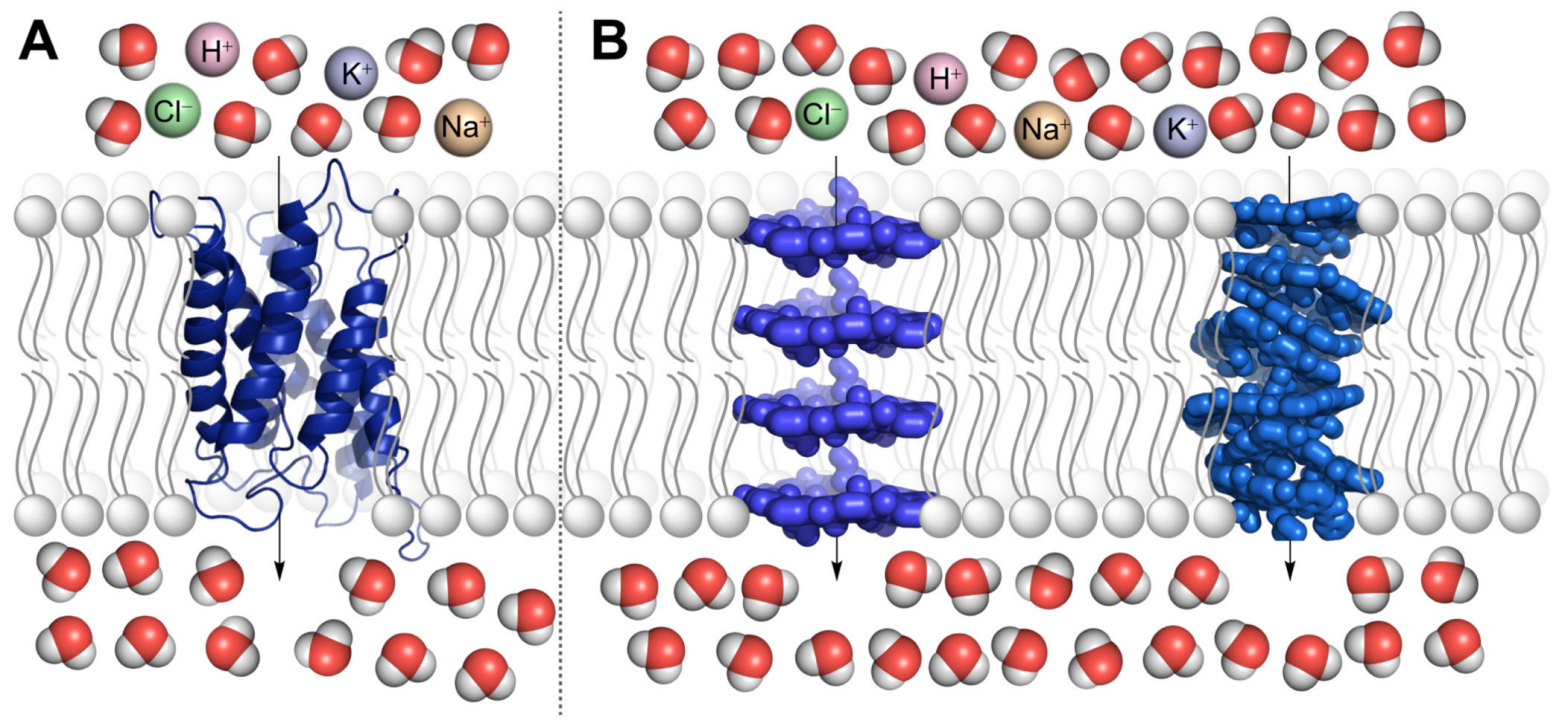
(A) Schematic representation of aquaporis and (B) water channel formation by macrocycle and helical polymers embedded in a vesicular phospholipid bilayer membrane, illustrating salt exclusion and water transport.

Inspired by this previous work, we now report a new class of helical polymers and macrocycles which show high water permeability and salt rejection, with both fluorine and hydrocarbons on the interior surface (Figure 1). The polymers are stabilized by H---F---O three center hydrogen bonds, promoting helical folding and rigidifying the structures. The polymers were synthesized by living chain polymerization, formed quantitatively with narrow polydispersities, and were characterized by ^1^H nuclear magnetic resonance spectroscopy (^1^H NMR), ^19^F nuclear magnetic resonance spectroscopy (^19^F NMR), matrix-assisted laser desorption ionization-time of flight mass spectrometry (MALDI-ToF), and size exclusion chromatoagphy (SEC) analysis.

We used chiral hydrocarbon side chains, which enabled the characterization of polymers and assessment of the stability of their helical folding *via* circular dichroism (CD) spectroscopy. Small-angle X-ray scattering (SAXS) analysis further supported the folded structure of the polymers in solution. Macrocycles formed from the same building blocks were characterized by single-crystal X-ray diffraction. Lipid vesicle studies showed high water channel activities by an osmotic shock assay, and effective salt exclusion by fluorescence measurements. Our systems are highly effective artificial water channels, formed in high yields from simple building blocks, and provide a route to scalable and effective water purification and desalination.

## Results and Discussion

Our system is based on the use of condensation agent **1** and monomer **2**, whose synthesis was achieved in six steps (54% overall yield, gram scale, SI Section 3). Key design principles were the incorporation of chiral hydrophobic alkyl chains, fluorine atoms to promote water transport and facilitate analysis, and the incorporation of a turn motif with a three-center H---F---H hydrogen bond.

**Figure 3:**
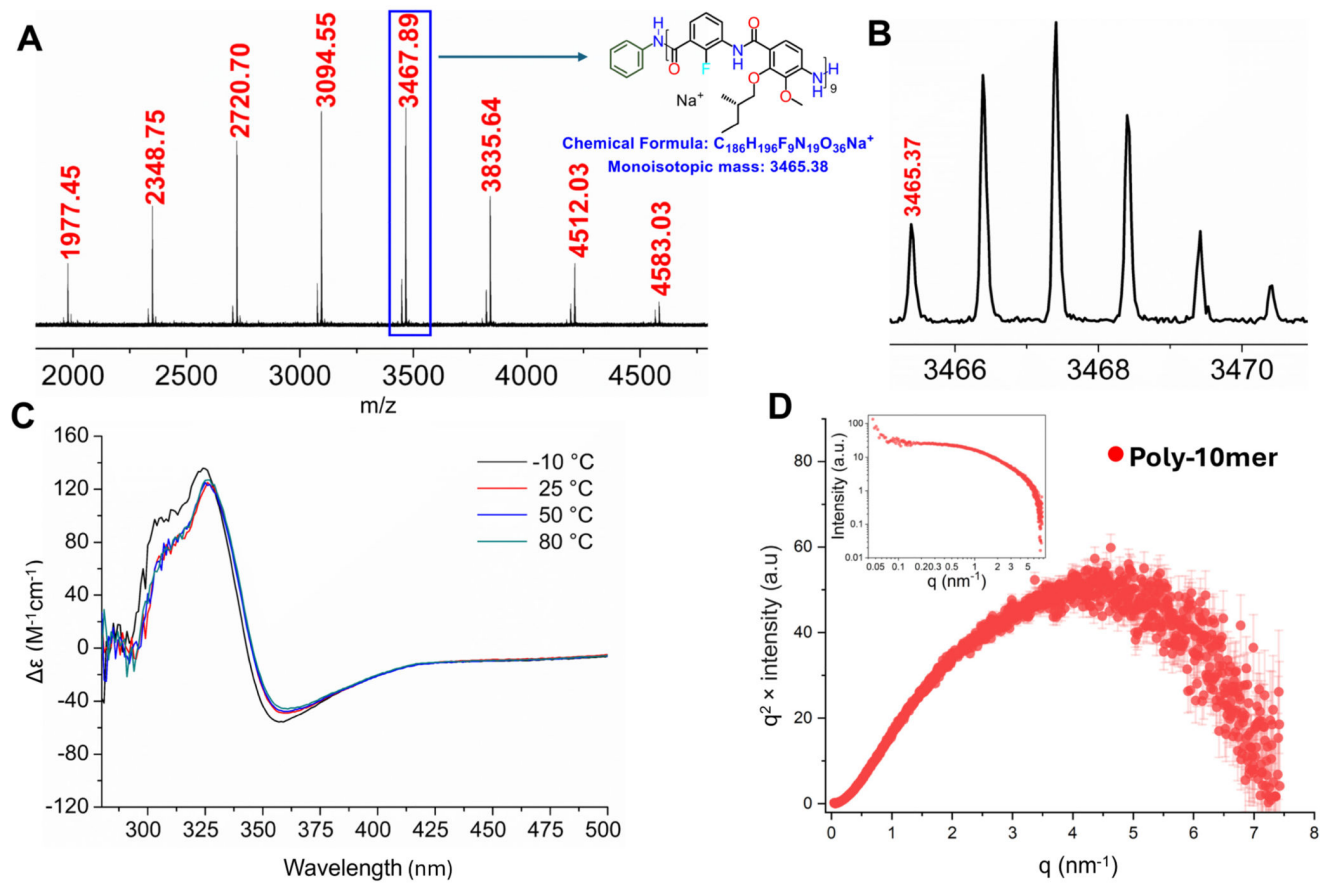
(A) Isotopically resolved MALDI-ToF mass spectrum (DCTB, NaTFA) of **Poly-10mer**. (B) Zoomed view of MALDI-ToF mass spectrum. (C) CD spectrum of **Poly-10mer** in toluene at different temperatures. (D) Kratky plot of the SAXS data of **Poly-10mer** in toluene/DMSO 9:1 v/v (and SAXS intensity *vs q* inset).

**Scheme 2:**
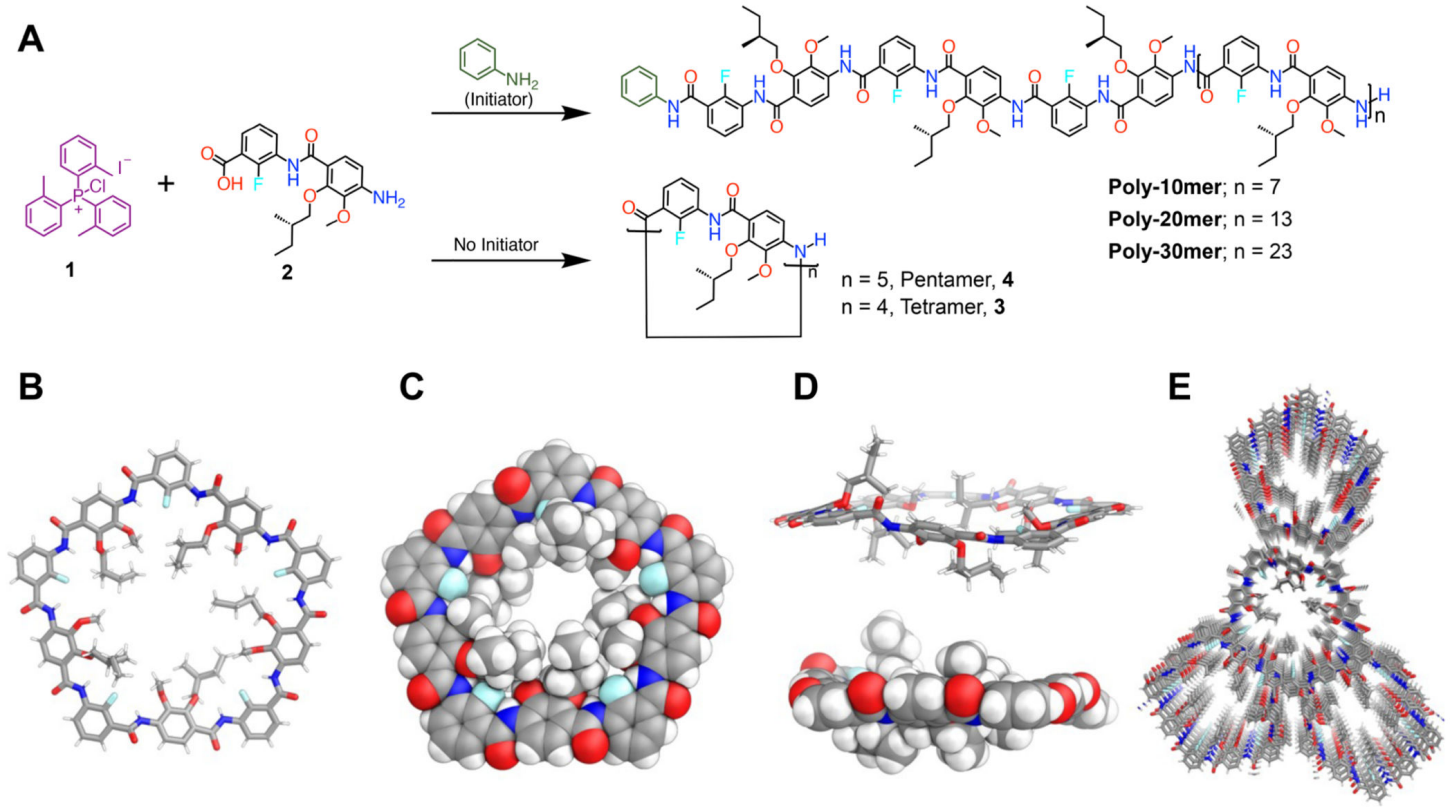
(A) (i) Slow addition of monomer **2** to a solution of **1** and initiator (aniline) at 55 °C yielding polymers **Poly-10mer** − **Poly-30mer** quantitatively (with respect to **2**). (ii) Reaction of monomer **2** with **1** at rt without an initiator yielding macrocycles **4** (5%) and **3** (1%) Conditions: **1** (3 eq.), Aniline **(1** eq.), **2** (10 - 30 eq.), Pyridine, CHCl_3_, 55 °C. (B) X-ray single crystal structure of **4**. (C) Space filling model of the X-ray single crystal structure of **4** top view and (D) side view. (E) 2D molecular packing along axis *b* (top view) showing accessible voids.

We performed a living polymerization using a procedure we recently reported (Scheme 2).^41,42^ **2** was added slowly (0.1 mL/h) to a solution containing an initiator (aniline) and the condensation agent, chloro-tri-o-tolylphosphonium iodide (**1**) (see Scheme S2, SI) at 55 °C. The crude mixture was concentrated and precipitated in cold methanol to obtain **Poly-10mer** in quantitative yield, which showed a narrow dispersity (Đ =1.14) and a number average molecular weight (M_n.sEc_ = 4.3 kDa) close to that expected from the [2]:[aniline] ratio of 10:1 (M_n-theo_ = 3.8 kDa). ^1^H NMR end-group analysis of **Poly-10mer** gave a molecular weight of 3.5 kDa, in good agreement with the theoretical target value and the data obtained by SEC. The identity of the end groups of **Poly-10mer** was further supported by an isotopically resolved MALDI-ToF mass spectrum (Figure 3A, B & Figure S36), with the major distribution consistent with aniline and amine end groups (SI Section 5, Figure S36).

To confirm the generality and robustness of the method, **Poly-20mer** and **Poly-30mer**, were synthesized, employing a 20:1 and 30:1 monomer-to-initiator ratio respectively. SEC analysis of **Poly-20mer** (M_n-SEC_ = 8.73 kDa, Đ =1.21, SI, Figure S39) and **Poly-30mer** (M_n-SEC_ = 11.14 kDa, Đ =1.13, SI, Figure S40) were in good agreement with the theoretical target values **(Poly-20mer:** M_n-theo_ = 7.5 kDa, and **Poly-30mer:** M_n-theo_= 11.2 kDa) (Scheme 2). A linear relationship between the monomer-to-initiator ratio and size of polymer formed was observed, confirming a living polymerization mechanism (SI Section 5, Figure S30 + Table Sl).

### Synthesis of macrocycles

To gain insight into the folded conformation of the polymer backbone, and to estimate the number of monomer residues likely in a single helical turn, we synthesized macrocyclic model compounds. Monomer **2** was reacted with **1** by slow addition (0.1 mL/h) in chloroform at room temperature, without an initiator (Scheme 2 & SI Section 3). After completion of the monomer addition, the crude product was concentrated and precipitated in methanol, and the resulting precipitate was subjected to silica gel flash chromatography using 20% diethyl ether in DCM to remove polymeric side products. The MALDI-ToF mass spectrum of the macrocycles revealed a major signal [m/z = 1884.10] corresponding to macrocycle **4** (as a sodium ion adduct), consisting of five units of monomer **2** and a minor signal [m/z = 1511.50] corresponding to macrocycle **3** (as a sodium ion adduct, Figure S34).

The reaction mixture was purified by preparative HPLC (10–20% diethyl ether in DCM), yielding pure **4** (5%) and **3 (1**%) as colorless solids. Due to the unoptimized macrocyclization conditions, significant polymeric byproducts were formed. The higher yield of **4** suggested that it may be the less strained of the two macrocycles and that a polymeric helical turn is likely close to five residues.

### X-Ray crystallographic analysis of oligomers and macrocycle

The single crystal x-ray structure of the dimer of monomer **2, 11** (SI Section 6),^43^ showed that the oligomers are rigidified by an inner rim of continuous three-centered hydrogen bonds (Figures S46 + S49). These three-centered hydrogen bonds should force polymers to fold into a helical conformation. The folded structure was confirmed by the X-ray crystal structure of the macrocycle 4,^43^ obtained by slow methanol diffusion into a chloroform solution. Single crystal diffraction data confirmed successful macrocyclization, with the top view of the structure (Scheme 2, B and C) showing a regular pentagonal shape with a defined, rigid, cavity of ca. 0.7 nm.

The chiral, hydrophobic alkoxy substituents and fluorine atoms must point inward in a macrocycle that forms all possible three-centered F--H--O- hydrogen bonds. However, a deviation from perfect planarity was observed (Figure 2, D), with the chiral, hydrophobic groups protruding above and below the macrocyclic plane due to their steric demands. Based on these observations, we assume a helical structure for the linear polymer in which all F--H--O hydrogen-bonds are formed.

It is, therefore, reasonable to assume that longer linear oligomers should adopt a similar rigidified folded structure, i.e. a helical conformation. Moreover, the packing pattern of macrocycle **4** along axis b showed the formation of nanochannels, stabilized by π-π stacking interactions (Figure 2, E). This strongly supports our ap plication of this system in lipid membranes to act as water channels, vide infra. We confirmed the solid-state assembly of macrocycle **4** by atomic force microscopy (AFM). A solution (4 mg/L) of **4** drop cast from CHCl3 solution onto a mica surface revealed linear fiber-like aggregates (SI Section 7, Figure S51).

### Conformational analysis of polymers in solution

The chiral side chains within the helical cavity induce a helical bias in the polymeric structure, allowing characterization by CD spectroscopy. At room temperature, we observed a strong signal from **Poly-10mer** in both toluene (Figure 3b & Figures S41 + S42) and CC1_4_(Figure S43). To evaluate the thermostability of the helical conformation of **Poly-10mer** in solution, temperature-dependent CD spectra were recorded. The CD signal remained nearly unchanged from +25 °C to +80 °C, indicating that the folded structure remains stable even at elevated temperatures, with a slight enhancement observed at -10 °C (Figure 3C, Figure S42). These results confirm the helical conformation of **Poly-10mer** in solution and highlight its exceptional thermal stability.

To further validate the folded structure of **Poly-10mer** in solution we conducted SAXS analysis. The SAXS data (Figure 3D) reveals an upturn at very low *q* values (< 0.07 nm^−1^), likely corresponding to large aggregates, confirmed by DLS (Figures S44 & S45). At higher *q* values, the data probes the subunits of these aggregates.

The intermediate flat region provides information about the size of the subunits. A Guinier analysis at the intermediate *q* values (from 0.3 nm^−1^ to 1.1 nm^−1^) yields an estimated radius of gyration of 1.1 nm for the overall size of the subunits (see Figure S56, SI) in hydrophobic solvent conditions (toluene/DMSO 9:1 v/v). Furthermore, the Kratky plot (*q*^2^ × intensity *vs q*) of **Poly-10mer** shown in Figure 3C exhibits a parabolic shape, with the curve converging to the baseline at high *q* values. Such a shape is characteristic of compacted or folded macromolecules.^1^ Therefore, this further confirms the folded structure of the **Poly-10mer** in solution.

### Water permeability across lipid bilayer membranes

**Figure 4:**
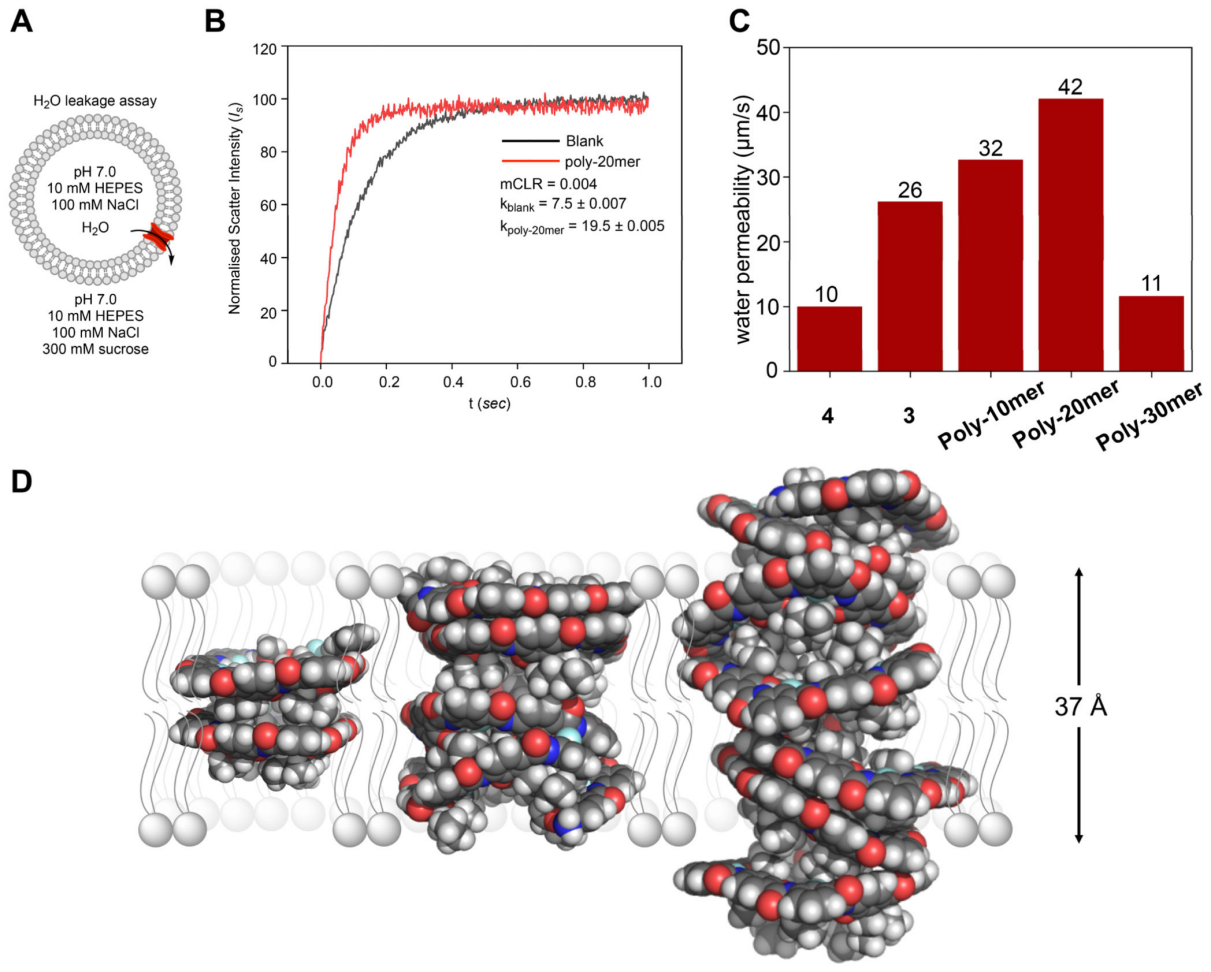
**(A)** Representation of water leakage assay. (B) Light scattering intensity vs. time plot of blank DOPC vesicles (blank) and DOPC vesicles with **Poly-20mer** with mCLR 0.004. (C) Comparison of corrected water permeability (corrected from blank) of all channels. (D) Molecular dynamics simulations of polymers with varying chain lengths, **Poly-10mer** (on left, L =18.7 Å), **Poly-20mer** (on middle, L = 33.1 Å), and **Poly-30mer** (on right L = 49.3Å) in octanol, showing size matching of **Poly-20mer** with DOPC bilayer (L = 36.7 Å) which shows the highest water flux.

The formation of nanochannels by macrocycle **4** in the solid state, and the folded structures of the polymers in hydrophobic solvents, encouraged us to investigate their water permeability and salt exclusion (ion permeation rates) across lipid membranes. We used a stopped-flow light scattering instrument to measure the light scattering intensity of vesicle suspensions on a millisecond scale. We prepared vesicles by mixing 1,2-dioleoyl-sn-glycero-3-phosphocholine (DOPC) with channel-forming compounds at a molar channel-to-lipid ratio (mCLR) of 0.004. We then extruded in 100 mM NaCl solution, buffered to pH 7.0 with HEPES (Figure 4A), forming vesicles with an average diameter of ~ 156 nm.^2^ Upon exposing these vesicles to a hypertonic solution (300 mM sucrose in pH 7.0 HEPES buffer), water efflux caused vesicle shrinkage, increasing light scattering intensity. Notably, we observed a significant rise in light scattering for both the macrocycles **(3** and **4)** and polymers **(Poly-10mer, Poly-20mer** and **Poly-30mer)** compared to controls, suggesting that all these molecules are able to facilitate water transport across lipid membranes (Figure 4A, B, & Figure S55, SI Section 9). Assuming 100% membrane insertion efficiency for all channels at mCLR = 0.004, the rate constants from scattering plots were used to calculate the permeability values for **3, 4, Poly-10mer, Poly-20mer**, and **Poly-30mer** using a previously published model (Figure 4C, SI Section 9).^13,39,3^

We then converted these values to estimate the single-channel water permeability, expressed as water molecules per second per channel. The resulting values were 7.6 × 10^7^, 8.02 × 10^7^, 1.0 × 10^8^, 1.3 × 10^8^, and 3.5 × 10^7^ for **3, 4 Poly-10mer**, **Poly-20mer**, and **Poly-30mer**, respectively. **Poly-20mer** is the most efficient water channel examined in this study, approaching the performance of biological aquaporins within an order of magnitude. To better understand the channel formation of **Poly-10mer** − **Poly-30mer** we reconsidered the X-ray single crystal structure of macrocycle **4**. The 2-methylbutoxy and methoxy side chains connected to the inside of macrocycle **4** prevent their close stacking in the solid state, with interplanar distances of c. 6.8 Å. This indicates that the space available on the inside of the macrocycle is insufficient to accommodate the total volume of all attached side chains. This in turn, suggests that polymers **Poly-10mer** − **Poly-30mer** may not form a tightly packed helix due to steric interactions of the interior chains. Estimating the tubular length of these helices is, therefore, difficult. For this reason, we employed molecular dynamics (MD) simulations to obtain a better estimate for the dimensions of the polymeric helical tubes in solution. MD simulations of individual polymer chains corresponding to **Poly-10mer** − **Poly-30mer** show that the number of repeat units per helical turn changes with the length of the modelled polymer. **Poly-10mer** has 4.5 repeat units per turn, but **Poly-30mer** requires 5 units per turn on average. This also affects the average length of the helical tube, with **Poly-10mer** exhibiting an average length of 18.7 Å, **Poly-20mer** 33.1 Å and **Poly-30mer** 49.3 Å.

Representative snapshots of the MD trajectory for each of the polymers are shown in Figure 4D. These show how the interior substitution of the helical tube prevents a tight helical fold, with substituents forcing openings in the helix. Considering that the hydrophobic thickness of a DOPC bilayer is 36.7 Å,^4^ and that **Poly-20mer** matches this length most closely, these results are in agreement with the observed water transport efficiency (Figure 4D). A good correspondence of the helical length with lipid bilayer thickness seems to be crucial for efficient water transport.

### Ion transport studies across lipid bilayer membrane

**Figure 5:**
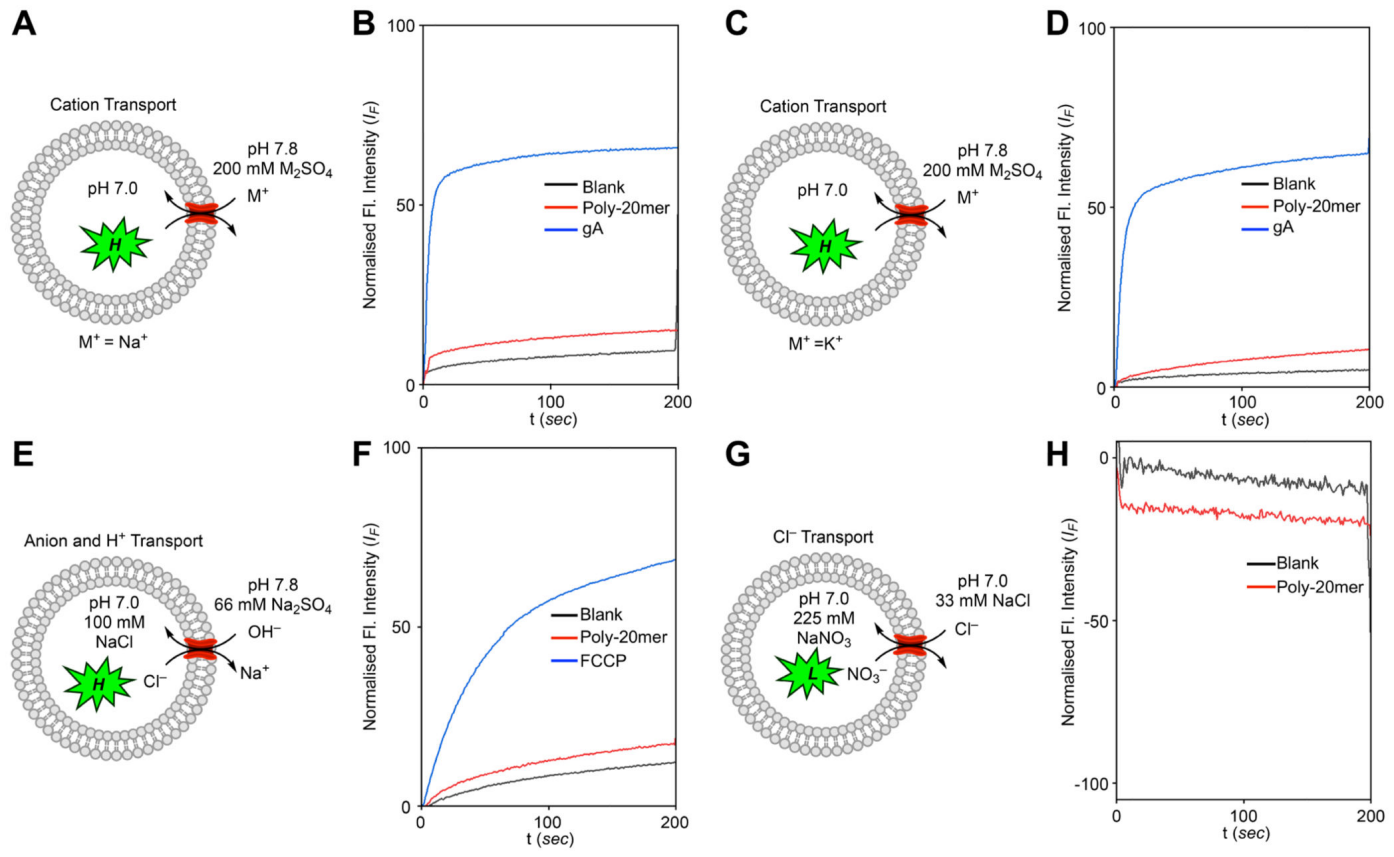
(A-B). Representation of vesicles and sodium ion transport by gA (0.1 µM) and **Poly-20mer** by HPTS assay. (C-D). Representation of vesicles and potassium ion transport by gA (0.1 µM) and **Poly-20mer** by HPTS assay. (E-F) Vesicular representations and assay details of anion and proton transport. The H^+^ transport activities of FCCP (0.2 µM) and **Poly-20mer** in HPTS assay (G-H). Representations of assay details and chloride anion transport of **Poly-20mer** across the EYPC-LUVs⊃Lucigenin (**Poly-20mer** =0.4 mol% with respect to lipid in all assays).

A key requirement for artificial water channels is effective salt exclusion. To verify the salt (Na^+^ and K^+^) rejection ability of our most effective channel, **Poly-20mer**, we conducted an ion transport experiment using LUVs prepared from egg yolk phosphatidylcholine (EYPC) lipids with the pH-sensitive fluorescent dye HPTS (8-hydroxypyrene-1,3,6-trisulfonic acid trisodium salt, **1** mM) encapsulated in a pH 7, 10 mM HEPES buffer.^18, 34^ Vesicles, containing pre-incorporated channel **Poly-20mer** (0.4mol% with respect to lipid), were then exposed to 200 mM M_2_ SO_4_ (M = Na^+^ or K^+^, (Figure 5A-D and Figure S56). The change in fluorescence intensity over time was monitored after adding NaOH to establish a pH gradient (ΔpH = 0.8) across the membrane. **Poly-20mer** showed minimal response to the Na^+^ (Figure 5B) and K^+^ (Figure 5D) gradients, confirming its impermeability to these cations. In contrast, gramicidin (a peptide cation channel) showed significant activity (~65 % for both Na^+^ and K^+^). These findings clearly demonstrate that our channels do not transport Na^+^ or K^+^ across the bilayer membrane at appreciable rates.

Furthermore, proton and anion translocation were assessed using a HPTS assay (Figure 5E + F). In an iso-osmolar environment, a pH gradient (ΔpH = 0.8) should drive a proton transporter to export protons, increasing HPTS fluorescence intensity. Similarly, an anion transporter would bring hydroxyl ions into the vesicles, increasing HPTS fluorescence intensity. **Poly-20mer** exhibited no significant transport activity, whereas carbonyl cyanide-4-(trifluoromethoxy)phenylhydrazone (FCCP, a proton carrier) demonstrated substantial transport activity (~70 %). These observations confirm that **Poly-20mer** does not transport protons or anions at appreciable rates.

Finally, we examined the chloride transport capabilities of **Poly-20mer** using the fluorescent dye lucigenin, which is quenched in the presence of chloride. Under intravesicular and extravesicular conditions of NaNO_3_ (225 mM) and NaCl (33.3 mM) (Figure 5 G + H), no lucigenin quenching occurred in the presence of **Poly-20mer**. This result confirms that water channel **Poly-20mer** effectively excludes anions, whilst retaining high levels of water transport activity.

## Conclusion

We have successfully demonstrated the synthetic design and characterization of functional polymers (**Poly-10mer, Poly-20mer, Poly-30mer**) and macrocycles (**3** and **4**) as high-performance artificial water channels. These structures were designed with inner cavities functionalized by fluorine and chiral alkyl substituents, imparting hydrophobicity and ion selectivity. The foldamers were synthesized *via* a living polymerization approach, yielding polymers with narrow dispersities and a simple, high yielding, purification by precipitation. The polymers were characterized using SEC, MALDI-ToF, and NMR (^1^H and ^1^ÅF). Circular Dichroism (CD) spectroscopy confirmed that the polymers fold to form helices, while SAXS analysis further supported their folded structure. Additionally, X-ray single-crystal structures of oligomers and macrocycles confirmed the presence of a key turn-inducing three-center hydrogen bond in the solid state and revealed the formation of nanochannels. We used this structural information to demonstrate the functional utility of these polymers and macrocycles in transporting water molecules across bilipid membranes with high efficiency and selectivity. **Poly-20mer** acted as the most efficient channel, with high water permeability, excellent salt and proton rejection, and performance similar to the evolutionarily optimized aquaporins, but showing vastly superior stability. Molecular dynamics (MD) simulations aligned closely with experimental data, with the high-water transport rate of **Poly-20mer** relating to its length, and optimal balance of flexibility and structural rigidity.

This work represents a significant advance in developing scalable and efficient artificial water channels, providing a robust platform for next-generation water purification and desalination technologies. Further optimization of these water channels is underway, seeking to achieve water transport rates beyond those of biological aquaporins.

## Supplementary Material

Figure S1
