# Supplementary material for: Rapid Water Permeation by Aramid Foldamer Nanochannels with Hydrophobic Interiors
Source: Angew Chem Int Ed Engl. Author manuscript; Available in PMC 2025 Apr 29. (PMC7617624; doi:10.1002/anie.202504170)
Supplement: Figure S1 [file EMS204452-supplement-Figure_S1.pdf]

Supplementary Information

Rapid Water Permeation by Aramid Foldamer Nanochannels with Hydrophobic Interiors

Saquib Farooq,[a] Javid Ahmad Malla,[b] Miroslava Nedyalkova,[a] Rafael V.M. Freire,[a] Indradip Mandal,[a] Aurelien Crochet,[a] Stefan Salentinig,[a] Marco Lattuada,[a] Charlie T. McTernan,[b,*] and Andreas F. M. Kilbinger.[a,*]

[a]Department of Chemistry, University of Fribourg, Chemin du Musée 9, CH-1700 Fribourg, Switzerland.

[b]Artificial Molecular Machinery Laboratory, The Francis Crick Institute, 1 Midland Road, London, NW1 1AT, UK and Department of Chemistry, Britannia House, 7 Trinity Street, King's College London, SE1 1DB, UK.

Table of Contents:

1. Materials 3
2. Instrumentation 3
   AFM measurements 3
   DLS measurements 4
   SAXS measurements 4
   Preparative HPLC 4
   MD stimulations 4
3. Experimental procedures 5
   Synthesis of monomer **2** 5
   Synthesis of precursor **5** 6
   Synthesis of precursor **8** 7
   Synthesis of precursor **9** 7
   Synthesis of precursor **10** 8
   Synthesis of **2** 8
   Synthesis of dimer **11** 9
   Synthesis of polymers 10
   Synthesis of macrocycles 11
4. NMR spectra 13
5. Polymer characterization data 28
6. X-Ray single Crystal data 38
7. AFM studies 47
8. HRMS studies 48
9. Water Transport Experiments 50
10. Ion Transport studies 53
11. Molecular dynamics simulations 57
12. Previous reported aramide water channels 59
13. References 59

1. Materials

2-methyl-butene, methyl iodide, *p*-toluene sulfonyl chloride (TsCl), *t*Bu*OH*, oxalyl chloride, anhydrous K_2CO_3, anhydrous tris(*o*-tolyl) phosphine, Pd on carbon (10 wt. % loading), and aniline were purchased from Sigma-Aldrich. 2-Fluoro-3-nitrobenzoic acid and (*S*)-2-methylbutan-1-ol were purchased from Fluorochem. 1 M iodine monochloride solution in dichloromethane was purchased from Acros Organics and used without further purification. Anhydrous pyridine, dry chloroform, dry *N*,*N*-dimethylformamide (DMF), *N*,*N*-dimethylacetamide (DMAc), dry *N*-methyl-2-pyrrolidone (NMP) were purchased from Acros Organics and used without further purification. Deuterated solvents (DMSO-d_6, and $CDCl_3$) were purchased from Cambridge Isotope Laboratories, Inc. All polymerizations were performed in flame-dried glassware. Florescence measurements were performed using FS-5 fluorometer (Edinburgh Instruments) connected to a temperature controller using Hellma quartz cuvettes. Lipids were purchased from Avanti Polar Lipids. HEPES buffer, HPTS, Lucigenin, Triton X–100, NaOH, and inorganic salts were purchased molecular biology grade from Sigma. Large unilamellar vesicles (LUVs) were prepared by using a mini extruder, equipped with a polycarbonate membrane of 200 nm pore size, purchased from Avanti Polar Lipids. Sephadex G-50 was used for Size exclusion Chromatography to isolate liposomes from dyes in their respective buffers. Carbonyl cyanide-*p*-trifluoromethoxyphenylhydrazone (FCCP) was purchased from Fluorochem and gramicidin A (gA) was purchased from Thermofischer.

2. Instrumentation

All ^{1}H NMR, ^{13}C NMR and ^{19}F NMR spectra were recorded on a Bruker Avance III (400 MHz and 300 MHz) FT NMR spectrometer. Chemical shifts for ^{1}H and ^{13}C were given in ppm relative to the residual solvent peak ($CDCl_3$: 7.276 for ^{1}H; $CDCl_3$: 77.16 for ^{13}C and DMSO-d_6: 2.50 for ^{1}H; DMSO-d_6: 39.51 for ^{13}C. MALDI-ToF mass spectra were measured on a Bruker ultraflextreme^{TM} using *trans*-2-[3-(*tert*-butylphenyl)-2-methyl-2-propenylidene]malononitrile (DCTB) as matrix and sodium trifluoroacetate (NaTFA) or silver trifluoroacetate (AgTFA) as the counter ion source. HRMS (ESI+) mass spectra were measured on double-focusing (BE geometry) magnetic sector mass spectrometer DFS (ThermoFisher Scientific, Bremen, Germany); solid probe inlet; EI at 70 eV; source temperature 200 °C; acceleration voltage 5 kV; electric scan mode; mass range 300–3500 m/z at 10,000 resolution (10% valley definition) and scan rate of 100–200 s per decade; mass accuracy ≤ 2 ppm after calibration with perfluorokerosene (PFK, Fluorochem, Derbyshire, UK). Relative molecular weights and molecular weight distributions were measured by size exclusion chromatography (SEC) with chloroform ($CHCl_3$) as eluent with a flow rate of 1 mL/min at 40 °C. The chloroform SEC system was calibrated with polystyrene standards, ranging from 10^3 to 3×10^6 Da with an automated PSS security System (Agilent Technologies 1260 infinity II) with a set of two MZ-Gel SD plus linear columns (300 x 8 mm, 5 µm particle size) for $CHCl_3$ SEC. Signals were recorded by an interferometric refractometer (Agilent 1260 series, as refractive index or RI traces). All polymer samples were filtered through a PTFE syringe membrane filter (0.45 µm pore size, VWR) before SEC measurements.

AFM measurements

AFM images were taken with the use of a Park NX10 instrument (Park Systems Corp., Suwon, Korea), equipped with Smart Scan software version 1.0 RTM. All measurements were performed in an acoustic enclosure (JPK Instruments AG, Berlin, Germany) equipped with antivibration table (e-Stable mini, Kurashiki Kako Co., LTD, Okayama, Japan). Tips used for imaging were TAP300AL-G (Budget Sensors, Sofia, Bulgaria) with a declared tip radius of less than 10 nm. All images were taken in tapping mode, scanning rate of 0.5 ± 0.3 Hz and with an imaging size of 1024 x 1024 or 2048 x 2048 pixels. Raw data was elaborated with the use of XEI software (Park Systems).

DLS measurements

Dynamic Light Scattering (DLS) measurements were carried out in Nano Lab 3D™ (LS Instruments, Switzerland) equipped with a 685 nm laser source. Measurements were performed at a 90° detector angle, averaging 10 repetitions of 10 seconds each per sample. The temperature was set to 25 °C for all measurements.

SAXS measurements

Small angle X-ray scattering (SAXS) measurements were performed with a SAXSPoint 5.0 (Anton Paar, Graz, Austria) equipped with a Ga MetalJet source (Excillum, Kista, Sweden) and with 2D EIGER R Hybrid Photon Counting (HPC) detectors (Dectris, Baden, Germany). Measurements were carried out at two sample-detector distances (1.608 m and 0.500 m) and the data was merged for broader q-range. Sample **Poly-10mer** was measured in a concentration of 5 mg/mL, at 25 °C, in borosilicate glass capillaries (2 mm thickness) for 3 hours. The 2D scattering profiles were radially integrated into 1D curves and plotted as a function of the scattering intensity versus the magnitude of the scattering vector q. A background sample (toluene:DMSO 9:1 v/v) was also measured, from which the sample scattering was subtracted. The Guinier analysis fit used to estimate radius of gyration is (Eq. S1):

$$\ln\big(I(q)\big) = \ln(I_0) - \frac{R_g^2}{3}\, q^2 \qquad Eq.S1$$

Where I_0 is the estimated intensity at $q = 0$ and R_g is the radius of gyration. The R_g can be estimated from the slope of a linear fit of ln(I(q)) versus q^2.

Preparative HPLC

Preparative high-performance liquid chromatography (HPLC) of Jasco (pump - PU-2087 Plus, UV/VIS detector-UV-2075 Plus) was used to purify the macrocycles with diethyl ether and DCM as eluent with a flow rate of 20 ml/min using preparative columns (250 × 20 mm) Lichrosphere-100 Si 10 μm.

MD stimulations

Simulations Protocol

Molecular dynamics simulations were performed using the GROMACS program.[S1-S3] The prepared initial structures for the **Poly-10mer**, **Poly-20mer**, and **Poly-30mer** were uploaded into the ATB topology builder[4] to get the optimized starting structure based on semiempirical QM theory for the MD simulation. Then, the initial energy minimization was done, followed by the MD simulation with the GROMOS54A7 force field. The molecules were placed at the center of a suitable cubic box depending on the length of the structure (4x4x4 nm^3, 5.5x5.5x5.5 nm^3, and 7.1x7.1x7.1 nm^3). The pre-equilibrated octanol box (.gro) was used. The octanol topology file (octanol.itp) was obtained from ATB server. Bonds involving hydrogen atoms were constrained using the SHAKE algorithm. The short-range electrostatic cut-off and the short-range van der Waals (vdW) cut-off was set at 1.2 nm, and the particle mesh Ewald (PME) method was used for long-range electrostatic interactions. The Nosé–Hoover thermostat with a coupling constant of τt = 1 ps was used for maintaining a constant temperature at T = 298.15 K, and the Parrinello–Rahman barostat with a coupling constant of τp = 1 ps was used for keeping the pressure at a constant value of P = 1 bar. The production simulation was run in the isothermal–isobaric ensemble (NPT) for 100 ns with a timestep of 2 fs.

3. Experimental procedures

Synthesis of monomer **2**

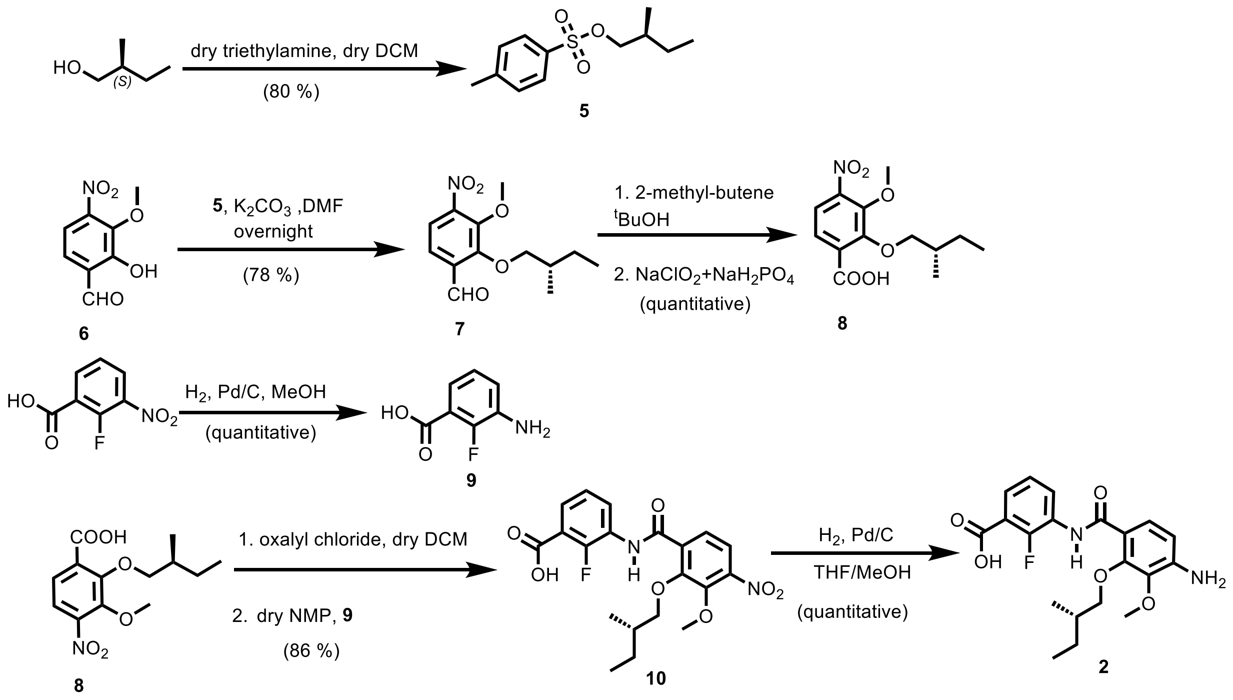

Scheme S1: Synthesis of monomer **2**.

Synthesis of precursor **5**

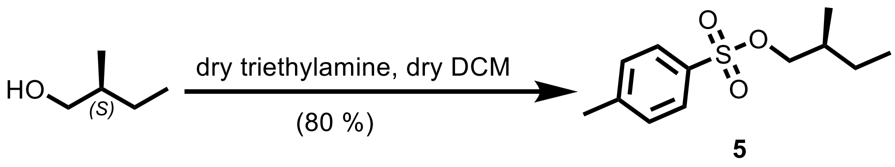

(*S*)-2-Methylbutyl 4-methylbenzenesulfonate (**5**): Commercially available (*S*)-2-methylbutan-1-ol (8.0 g, 9 mmol, 1 eq) and dry triethylamine (19 mL, 1.5 eq) was dissolved in dry dichloromethane at 0 °C. After 10 minutes *p*-toluene sulfonyl chloride (TsCl) was added portion-wise, and the reaction was stirred overnight at room temperature. The solvent was concentrated under reduced pressure and was extracted with dichloromethane. The organic portion was washed with brine, $NaHCO_3$ and water. The crude product was purified by column chromatography with hexane: diethyl ether (95:5) to obtain **5** (17.8 g, yield 80 %) as a colorless liquid. ^{1}H NMR (400 MHz, $CDCl_3$) δ 7.77 (d, *J* = 8.3 Hz, 2H), 7.33 (d, *J* = 8.0 Hz, 2H), 3.84 (ddd, *J* = 26.4, 9.4, 6.1 Hz, 2H), 2.44 (s, 3H), 1.68 (dt, *J* = 12.8, 6.4 Hz, 1H), 1.43 – 1.31 (m, 1H), 1.18 – 1.06 (m, 1H), 0.86 (d, *J* = 6.8 Hz, 3H), 0.81 (t, *J* = 7.5 Hz, 3H). ^{13}C NMR (101 MHz, $CDCl_3$) δ 144.73 (s), 133.32 (s), 129.91 (s), 128.00 (s), 77.48 (s), 77.16 (s), 76.84 (s), 74.94 (s), 34.47 (s), 25.55 (s), 21.74 (s), 16.07 (s), 11.06 (s).

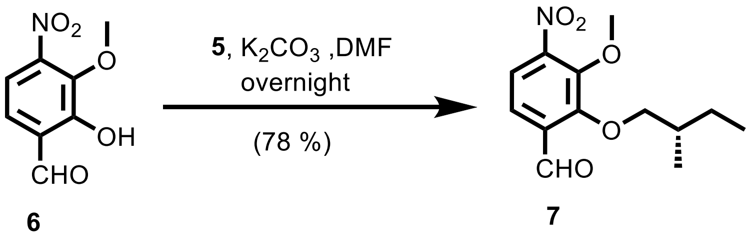

(*S*)-3-Methoxy-2-(2-methylbutoxy)-4-nitrobenzaldehyde (**7**): 2-hydroxy-3-methoxy-4-Nitro benzaldehyde **6**[5] (4 g, 20 mmol, 1 eq) and K_2CO_3 (5 g, 1.8 eq) was dissolved in 20 mL of DMF. After 30 minutes **5** (6.0 g, 1.3 eq) was added and the reaction was stirred overnight at 60 °C. The conversion was monitored by HPLC. The crude reaction was filtered. The filtrate was extracted with ethyl acetate and water, (3 times) and the organic portion was washed with brine and dried over $MgSO_4$ and concentrated in vacuo. The crude product was purified by column chromatography with hexane: ethyl-acetate (90:10) to obtain **7** (4 g, 78 %) as a light-yellow liquid. ^{1}H NMR (400 MHz, $CDCl_3$) δ 10.42 (d, *J* = 0.9 Hz, 1H), 7.65 (d, *J* = 8.6 Hz, 1H), 7.49 (dd, *J* = 8.6, 0.8 Hz, 1H), 4.06 (dd, *J* = 9.1, 5.8 Hz, 1H), 4.01 (s, 4H), 3.99 – 3.96 (m, 1H), 1.99 – 1.86 (m, 1H), 1.59 (dqd, *J* = 14.9, 7.5, 5.5 Hz, 1H), 1.37 – 1.27 (m, 1H), 1.08 (d, *J* = 6.8 Hz, 3H), 0.97 (t, *J* = 7.5 Hz, 3H). ^{13}C NMR (101 MHz, $CDCl_3$) δ 188.48 (s), 157.42 (s), 149.08 (s), 147.44 (s), 132.93 (s), 123.03 (s), 119.16 (s), 81.05 (s), 77.48 (s), 77.16 (s), 76.84 (s), 62.71 (s), 35.84 (s), 26.06 (s), 16.57 (s), 11.40 (s).

Synthesis of precursor 8

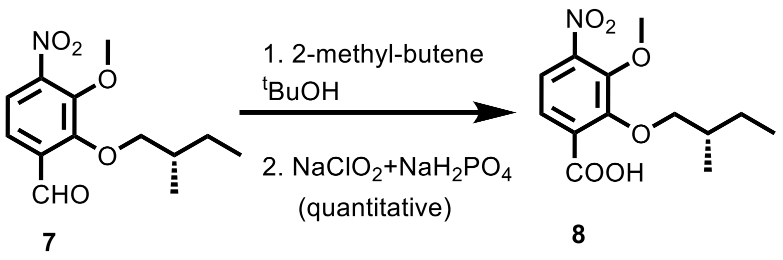

(*S*)-3-Methoxy-2-(2-methylbutoxy)-4-nitrobenzoic acid (**8**): 80% $NaClO_2$ in 1 M aq. NaH_2PO_4 at room temperature was added dropwise to a stirred solution of **7** (4 g, 14.9 mmol,1 eq.) and 20 mL of 2-methyl-butene in 44 mL of *t*BuOH. The conversion was monitored by HPLC. Upon completion, the mixture was concentrated in a vacuo and was extracted with ethyl acetate and water. The organic layer was washed with 1N HCl, brine and water, dried over $MgSO_4$ and concentrated in vacuo to yield **8** (4 g, 97 %) as a light-yellow solid. ^{1}H NMR (400 MHz, $CDCl_3$) δ 7.95 (d, *J* = 8.7 Hz, 1H), 7.58 (d, *J* = 8.7 Hz, 1H), 4.16 (dd, *J* = 8.9, 5.9 Hz, 1H), 4.07 (dd, *J* = 8.9, 6.8 Hz, 1H), 4.01 (s, 3H), 1.97 (dd, *J* = 13.0, 6.3 Hz, 1H), 1.59 (ddd, *J* = 9.5, 7.4, 3.7 Hz, 1H), 1.39 – 1.27 (m, 1H), 1.10 (d, *J* = 6.8 Hz, 3H), 0.98 (dd, *J* = 9.4, 5.5 Hz, 3H). ^{13}C NMR (101 MHz, $CDCl_3$) δ 164.55 (s), 153.85 (s), 148.30 (s), 147.02 (s), 127.52 (s), 127.09 (s), 119.72 (s), 77.48 (s), 77.16 (s), 76.84 (s), 35.74 (s), 25.98 (s), 16.43 (s), 11.32 (s).

Synthesis of precursor 9

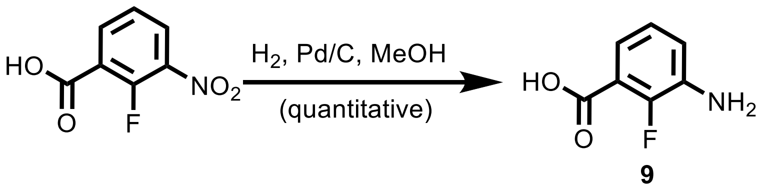

3-Amino-2-fluorobenzoic acid (**9**): 2-Fluoro-3-nitrobenzoic acid (5 g) was dissolved in the methanol. To the resulting mixture, Pd/C (10 wt. % loading) was slowly added and hydrogenated overnight under a hydrogen atmosphere (20 bar). The crude mixture was filtered through celite, and the filtrate was concentrated under reduced pressure to obtain the pure product as a red solid. ^{1}H NMR (400 MHz, DMSO) δ 12.87 (s, 1H), 7.04 – 6.64 (m, 3H), 5.29 (s, 2H). ^{19}F NMR (377 MHz, DMSO) δ -133.54 (dd, *J* = 9.0, 3.9 Hz). ^{13}C NMR (101 MHz, DMSO) δ 165.84 (s), 150.59 (s), 148.11 (s), 137.51 (d, *J* = 13.1 Hz), 123.82 (d, *J* = 4.1 Hz), 119.58 (d, *J* = 5.4 Hz), 117.42 (s), 40.65 – 40.26 (m), 40.26 – 39.71 (m), 39.73 (s), 39.62 (d, *J* = 21.0 Hz), 39.31 (s), 39.10 (s), 38.89 (s).

Synthesis of precursor **10**

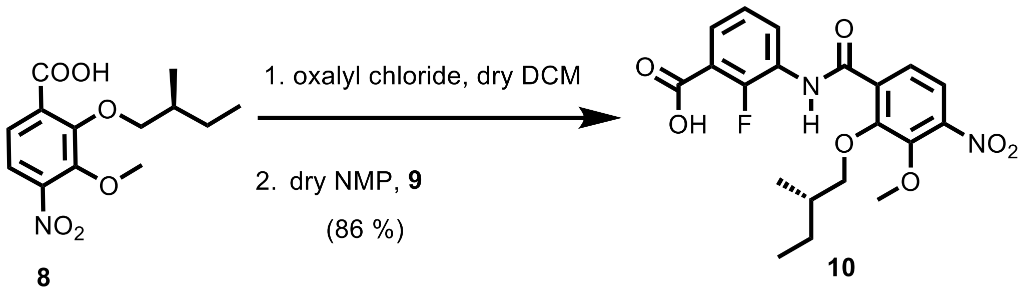

(*S*)-2-Fluoro-3-(3-methoxy-2-(2-methylbutoxy)-4-nitrobenzamido) benzoic acid (**10**): (*S*)-3-methoxy-2-(2-methylbutoxy)-4-nitrobenzoic acid **8** (4 g, 14.4 mmol, 1 eq) was dissolved in dry dichloromethane under nitrogen flush and 1.1 eq of oxalyl chloride was added followed by two drops of DMF and the reaction mixture was stirred at room temperature for 2-3 hours. The solvent was evaporated, and the resulting acid chloride was dissolved in dry NMP (20mL). The 3-amino-2-fluorobenzoic acid **9** (2.4 g, 15.1 mmol, 1.0 eq) was dissolved in dry NMP (5 mL) and was added dropwise to the resulting acid chloride solution. The reaction mixture was stirred overnight at room temperature. The crude reaction was precipitated in cold water (three-fold than NMP used in the reaction). The resulting precipitate was filtered out and washed in the methanol to obtain the pure product **10** (5.5 g yield, 86 %) as a white solid. ^{1}H NMR (400 MHz, $CDCl_3$) δ 10.20 (d, *J* = 3.1 Hz, 1H), 8.86 – 8.79 (m, 1H), 8.06 (d, *J* = 8.8 Hz, 1H), 7.82 (ddd, *J* = 8.4, 7.0, 1.7 Hz, 1H), 7.63 (d, *J* = 8.8 Hz, 1H), 7.31 (t, *J* = 8.1 Hz, 1H), 4.15 – 3.99 (m, 5H), 2.02 (dd, *J* = 12.9, 6.6 Hz, 1H), 1.59 (ddd, *J* = 9.9, 7.6, 3.8 Hz, 1H), 1.38 – 1.20 (m, 3H), 1.07 (d, *J* = 6.7 Hz, 1H), 0.93 (t, *J* = 7.5 Hz, 3H). ^{19}F NMR (377 MHz, $CDCl_3$) δ -125.80 (d, *J* = 6.5 Hz). ^{13}C NMR (126 MHz, $CDCl_3$) δ 168.85 (d, *J* = 2.6 Hz), 161.72 (s), 153.68 (s), 152.78 (s), 151.62 (s), 147.56 (s), 147.25 (s), 130.77 (s), 127.77 – 127.54 (m), 127.42 (s), 126.75 (s), 124.61 (d, *J* = 4.4 Hz), 119.69 (s), 117.60 (d, *J* = 8.0 Hz), 81.76 (s), 63.06 (s), 35.53 (s), 26.02 (s), 16.39 (s), 11.17 (s).

Synthesis of **2**

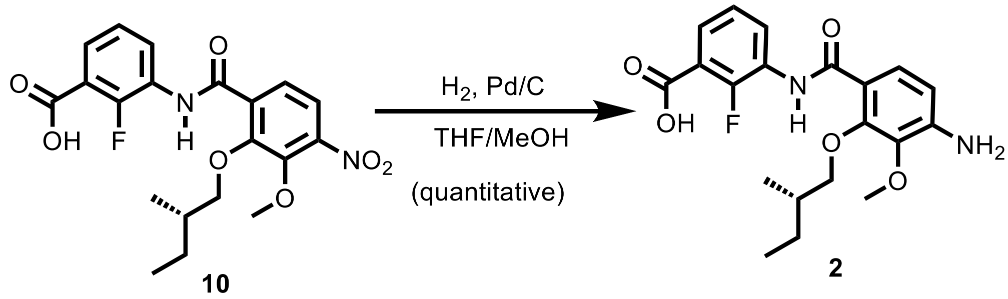

(*S*)-3-(4-Amino-3-methoxy-2-(2-methylbutoxy)benzamido)-2-fluorobenzoic acid (**2**): (*S*)-2-Fluoro-3-(3-methoxy-2-(2-methylbutoxy)-4-nitrobenzamido)benzoic acid **10** (3.5 g) was dissolved in the methanol/THF (60:40). To the resulting mixture Pd/C (10 wt. % loading) was slowly added and hydrogenated for overnight under hydrogen atmosphere (20 bar) at 30 °C. The crude mixture was filtered through celite, and filtrate was concentrated under reduced pressure to obtain the pure product **2** as a red solid (3.23 g, quant.). ^{1}H NMR (300 MHz, $CDCl_3$) δ 10.35 (d, *J* = 3.1 Hz, 1H), 8.91 – 8.82 (m, 1H), 7.82 (d, *J* = 8.7 Hz, 1H), 7.76 – 7.67 (m, 1H), 7.29 – 7.21 (m, 1H), 6.59 (d, *J* = 8.7 Hz, 1H), 4.11 – 3.82 (m, 5H), 2.02 (dd, *J* = 12.8, 6.7 Hz, 1H), 1.60 (ddd, *J* = 13.0, 7.4, 5.3 Hz, 1H), 1.34 – 1.19 (m, 1H), 1.08 (dd, *J* = 16.0, 3.8 Hz, 3H), 0.93 (t, *J* = 7.4 Hz, 3H). ^{19}F NMR (282 MHz, $CDCl_3$) δ -126.12 (s). ^{13}C NMR

(75 MHz, $CDCl_3$) δ 168.97 (s), 163.99 (s), 151.95 (s), 145.49 (s), 138.87 (s), 128.57 (d, *J* = 10.1 Hz), 127.90 (s), 127.34 (s), 125.99 (s), 124.20 (d, *J* = 4.5 Hz), 115.61 (s), 110.77 (s), 80.55 (s), 60.33 (s), 35.28 (s), 26.02 (s), 16.39 (s),11.10 (s).

MS (HR-MS): m/z calculated for $C_{20}H_{22}FN_2O_5^-$ $(M\text{-}H)^-$ = 389.1518, Found $[M\text{-}H]^-$ = 389.1519 (Δppm = 0.26)

Synthesis of dimer **11**

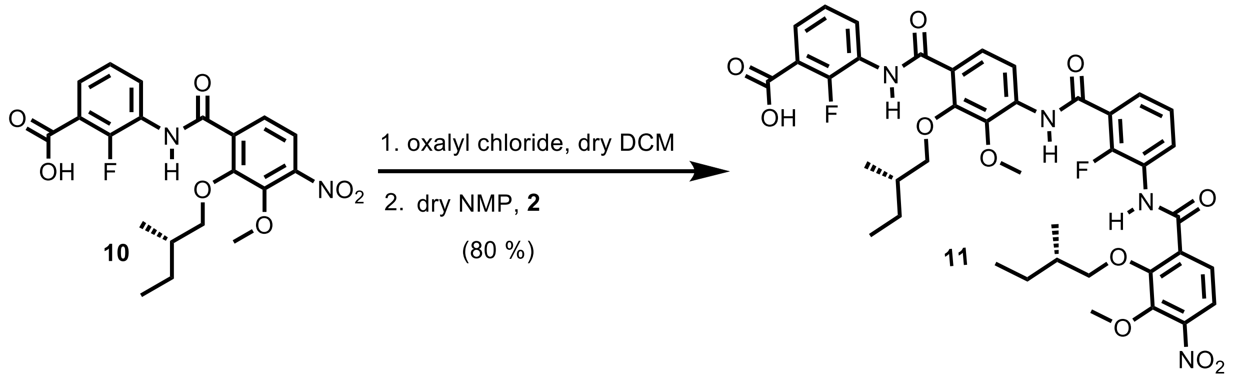

(*S*)-3-Methoxy-2-(2-methylbutoxy)-4-nitrobenzoic acid **10** (1 g, 2.37 mmol, 1 eq) was dissolved in dry dichloromethane under nitrogen and 1.5 eq of oxalyl chloride was added, followed by two drops of DMF, and the reaction mixture was stirred at room temperature for 3 hours. The solvent was evaporated, and the resulting acid chloride was dissolved in dry NMP. (*S*)-3-(4-amino-3-methoxy-2-(2-methylbutoxy)benzamido)-2-fluorobenzoic acid **2** (1 g, 2.37 mmol, 1 eq) was dissolved in dry NMP and was added dropwise to the resulting acid chloride solution. The reaction mixture was stirred overnight at room temperature. The crude reaction was precipitated in cold water (threefold than NMP used in the reaction). The resulting precipitate was filtered out and washed with methanol to obtain the pure product **11** as a white solid (1.52 g). ^1H NMR (400 MHz, $CDCl_3$) δ 10.25 (d, *J* = 3.3 Hz, 1H), 10.14 (d, *J* = 2.4 Hz, 1H), 9.19 (d, *J* = 13.1 Hz, 1H), 8.91 – 8.84 (m, 1H), 8.72 (td, *J* = 8.0, 1.7 Hz, 1H), 8.51 (d, *J* = 8.9 Hz, 1H), 8.08 (dd, *J* = 12.6, 8.9 Hz, 2H), 7.93 (td, *J* = 7.8, 1.7 Hz, 1H), 7.79 – 7.73 (m, 1H), 7.66 (d, *J* = 8.8 Hz, 1H), 7.41 (t, *J* = 8.0 Hz, 1H), 7.29 (t, *J* = 8.3 Hz, 1H), 4.16 – 4.10 (m, 4H), 4.07 (s, 3H), 3.99 (d, *J* = 2.6 Hz, 3H), 1.89 – 1.82 (m, 1H), 1.66 – 1.56 (m, 3H), 1.36 – 1.27 (m, 4H), 1.12 (d, *J* = 6.7 Hz, 3H), 1.07 (d, *J* = 6.7 Hz, 3H), 0.94 (td, *J* = 7.5, 3.5 Hz, 6H). ^{19}F NMR (377 MHz, $CDCl_3$) δ -126.35 (s), -131.50 (s) ^{13}C NMR (101 MHz, $CDCl_3$) δ 167.58 (s), 162.98 (s), 161.56 (s), 152.59 (s), 150.53 (s), 147.45 (s), 147.15 (s), 141.52 (s), 136.49 (s), 130.56 (s), 127.93 (s), 127.18 (d, *J* = 27.9 Hz), 126.99 – 126.77 (m), 126.62 (d, *J* = 23.8 Hz), 125.54 (s), 124.39 (s), 121.88 (s), 119.77 (s), 115.84 (s), 81.55 (s), 81.00 (s), 63.02 (s), 61.45 (s), 35.54 (s), 35.29 (s), 25.96 (d, *J* = 13.9 Hz), 16.36 (d, *J* = 8.4 Hz), 11.08 (d, *J* = 4.7 Hz).

MS (HR-MS): m/z calculated for $C_{40}H_{41}F_2N_4O_{11}^-$ $(M\text{-}H)^-$ = 791.2745 Found $[M\text{-}H]^-$ = 791.2758 (Δppm = 1.64).

Synthesis of polymers

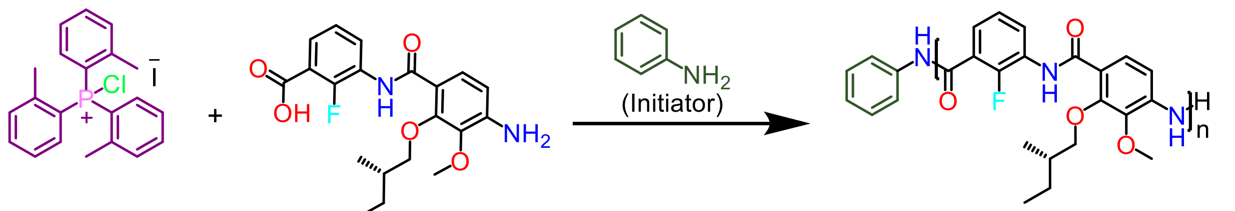

The required amount of **PHOS3** (**1**) was weighed (3 eq. of **1**) into a Schlenk flask and dissolved in dry chloroform (0.24 M). To the resulting **PHOS3** solution, appropriate amount of initiator solution (aniline, 2.5 mg, 0.027 mmol, 1 eq.) and anhydrous pyridine were added. In a separate Schlenk flask monomer **2** (10-30 eq. relative to initiator aniline) was dissolved in dry chloroform (0.2 M) under argon atmosphere. Then, the monomer **2** solution was added dropwise to the reaction mixture at 55 °C using a syringe pump (0.1 mL/h). Thereafter, the reaction mixture was concentrated under high vacuum and precipitated into excess methanol to obtain the polymer in quantitative yield.

Poly-10mer - ^{1}H NMR (400 MHz, $CDCl_3$) δ 10.18 (d, *J* = 31.2 Hz, 10H), 9.18 (dd, *J* = 24.4, 13.1 Hz, 9H), 8.72 (d, *J* = 7.3 Hz, 9H), 8.52 (d, *J* = 8.7 Hz, 9H), 8.08 (t, *J* = 7.6 Hz, 9H), 7.83 (d, *J* = 7.8 Hz, 9H), 7.36 (s, 13H), 6.60 (d, *J* = 8.4 Hz, 1H), 4.15 – 3.95 (m, 44H), 2.07 (d, *J* 7.2 Hz, 9H), 1.61 (s, 13H), 1.28 (d, *J* = 22.3 Hz, 13H), 1.09 (dd, *J* = 19.9, 6.3 Hz, 30H), 1.00 – 0.87 (m, 30H). ^{19}F NMR (377 MHz, $CDCl_3$) δ -130.76 (s), -131.10 (s), -131.45 (s), -131.57 (s).

Poly-20mer - ^{1}H NMR (400 MHz, $CDCl_3$) δ 10.14 (dd, *J* = 30.2, 20.9 Hz, 16H), 9.12 (d, *J* = 12.1 Hz, 15H), 8.67 (d, *J* = 7.9 Hz, 17H), 8.50 (t, *J* = 8.9 Hz, 15H), 8.11 – 8.01 (m, 16H), 7.83 (d, *J* = 8.7 Hz, 16H), 7.35 (d, *J* = 7.3 Hz, 19H), 6.60 (d, *J* = 8.5 Hz, 1H), 4.15 – 3.93 (m, 78H), 2.04 (d, *J* = 5.7 Hz, 20H), 1.39 – 1.23 (m, 32H), 1.11 (dd, *J* = 8.7, 4.8 Hz, 50H), 0.99 – 0.89 (m, 49H). ^{19}F NMR (377 MHz, $CDCl_3$) δ -131.03 (s), -131.37 –-131.42 (m).

Poly-30mer - ^{1}H NMR (400 MHz, $CDCl_3$) δ 10.12 (dd, *J* = 17.0, 9.7 Hz, 31H), 9.12 (d, *J* = 12.1 Hz, 31H), 8.82 – 8.62 (m, 31H), 8.51 (d, *J* = 8.8 Hz, 31H), 8.07 (d, *J* = 7.7 Hz, 30H), 7.84 (s, 29H), 7.36 (t, *J* = 7.4 Hz, 35H), 6.60 (d, *J* = 8.6 Hz, 1H), 4.16 – 3.91 (m, 156H), 2.04 (d, *J* = 7.3 Hz, 40H), 1.61 (d, *J* = 7.6 Hz, 83H), 1.37 – 1.23 (m, 85H), 1.15 – 1.06 (m, 91H), 0.94 (td, *J* = 7.2, 3.6 Hz, 96H). ^{19}F NMR (377 MHz, $CDCl_3$) δ -131.20 (d, *J* = 131.8 Hz), -131.40 – -131.93 (m), -132.30 – -133.06 (m).

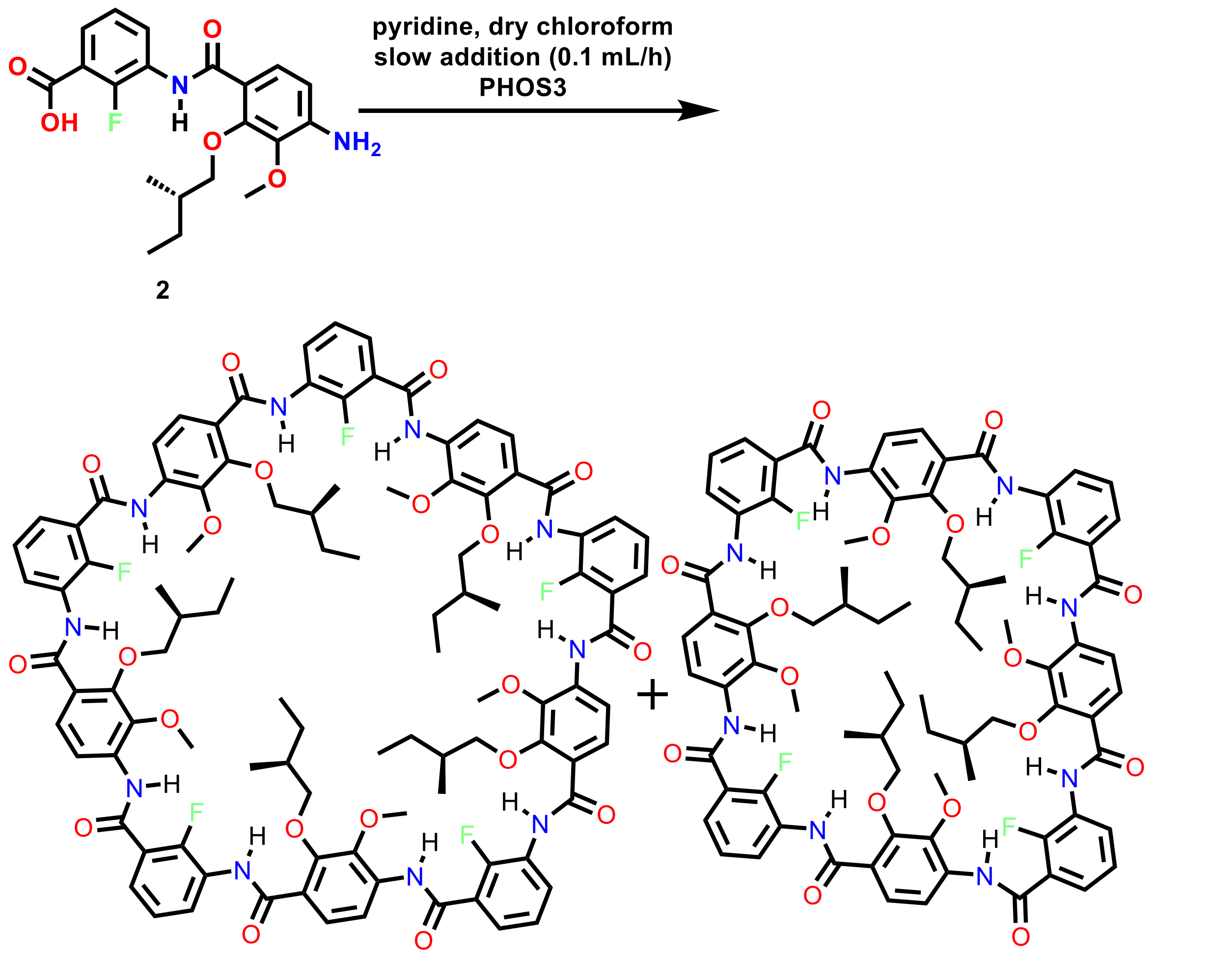

4 **3**

Scheme S2: Synthesis of macrocycles.

PHOS-3 reagent (**1**, 300 mg, 0.642 mmol, 2.5 eq.) was weighted in a Schlenk flask inside a glove box and dissolved in 15 mL of dry chloroform. To this solution, anhydrous pyridine (0.165 mL, 8 eq.) was added. The Monomer **2** (100 mg, 0.256 mmol, 1 eq.) was dissolved in chloroform (2.56 mL) and was added dropwise to the reaction mixture at room temperature using a syringe pump (0.1 mL/h) under argon. The crude reaction mixture was concentrated under reduced pressure, and the resulting crude mixture was precipitated in cold methanol to get rid of pyridinium salt and phosphine oxide. The resulting precipitate was subjected to a short-pack silica column (20% diethyl ether in DCM). The solution was concentrated and was subjected to preparatory HPLC using 10-20 % diethyl ether in dichloromethane to yield (5 %) pentamer (**4**) and (1 %) tetramer (**3**) macrocycles.

Pentamer Macrocycle (**4**) white solid.^{1}H NMR (400 MHz, $CDCl_3$) δ 10.27 (d, *J* = 2.5 Hz, 1H), 9.33 (d, *J* = 15.2 Hz, 1H), 8.92 (td, *J* = 8.0, 1.6 Hz, 1H), 8.62 (d, *J* = 9.0 Hz, 1H), 8.16 (d, *J* = 9.0 Hz, 1H), 7.98 (td, *J* = 7.8, 1.7 Hz, 1H), 7.42 (t, *J* = 8.0 Hz, 1H), 4.48 – 3.79 (m, 2H), 2.20 (dd, *J* = 12.9, 6.5 Hz, 1H), 1.69 (ddd, *J* = 13.2, 7.4, 5.5 Hz, 1H), 1.45 – 1.34 (m, 3H), 1.24 (t, *J* = 6.5 Hz, 3H), 1.04 (t, *J* = 7.5 Hz, 1H).^{13}C NMR (126 MHz, $CDCl_3$) δ 163.02 (s), 161.18 (s), 151.69 (s), 150.64 (s), 149.76 (s), 141.56 (s), 137.12 (s), 128.60 (s), 127.52 (d, *J* = 12.2 Hz), 126.90 (d, *J* = 36.6 Hz), 125.71 (s), 121.48 (s), 120.78 (d, *J* = 10.2 Hz), 116.52 (s), 81.07 (s), 77.63 (d, *J* = 26.9 Hz), 77.41 (s), 77.16 (s), 76.91 (s), 61.73 (s), 35.60 (s), 29.85 (s), 26.28 (s), 16.72 (s), 11.38 (s). ^{19}F NMR (377 MHz, $CDCl_3$) δ -132.77 (d, *J* = 6.4 Hz).

MS (MALDI-TOF) m/z, calcd for $C_{100}H_{105}F_5N_{10}O_{20}$ 1860.74 (M^+), found 1884.10 ($M+Na^+$).

Tetramer-Macrocycle (**3**). White solid. ^1H NMR (500 MHz, $CDCl_3$) δ 10.51 (s, 1H), 9.42 (d, *J* = 15.4 Hz, 1H), 8.91 (t, *J* = 7.7 Hz, 1H), 8.52 (d, *J* = 8.7 Hz, 1H), 8.07 (d, *J* = 8.8 Hz, 1H), 7.86 (t, *J* = 7.2 Hz, 1H), 7.41 (t, *J* = 8.0 Hz, 1H), 4.13 – 3.96 (m, 5H), 2.04 (s, 1H), 1.37 – 1.30 (m, 2H), 1.13 (d, *J* = 6.7 Hz, 3H), 0.92 (t, *J* = 7.5 Hz, 3H). ^{19}F NMR (471 MHz, $CDCl_3$) δ -132.84 (s).
MS (MALDI-TOF) m/z, calcd for $C_{80}H_{84}F_4N_8O_{16}$ 1488.59 (M^+), found 1511.51 ($M+Na^+$).

4. NMR spectra

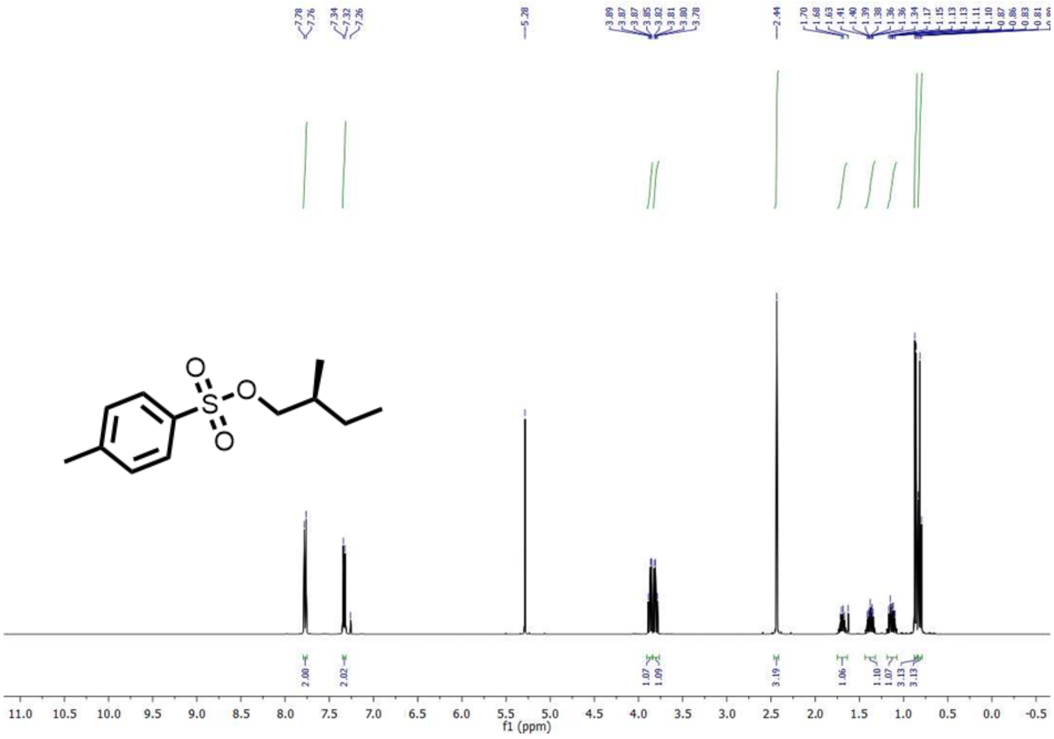

Figure S1. ^{1}H NMR spectrum (400 MHz, $CDCl_3$ at 298 K) of **5**.

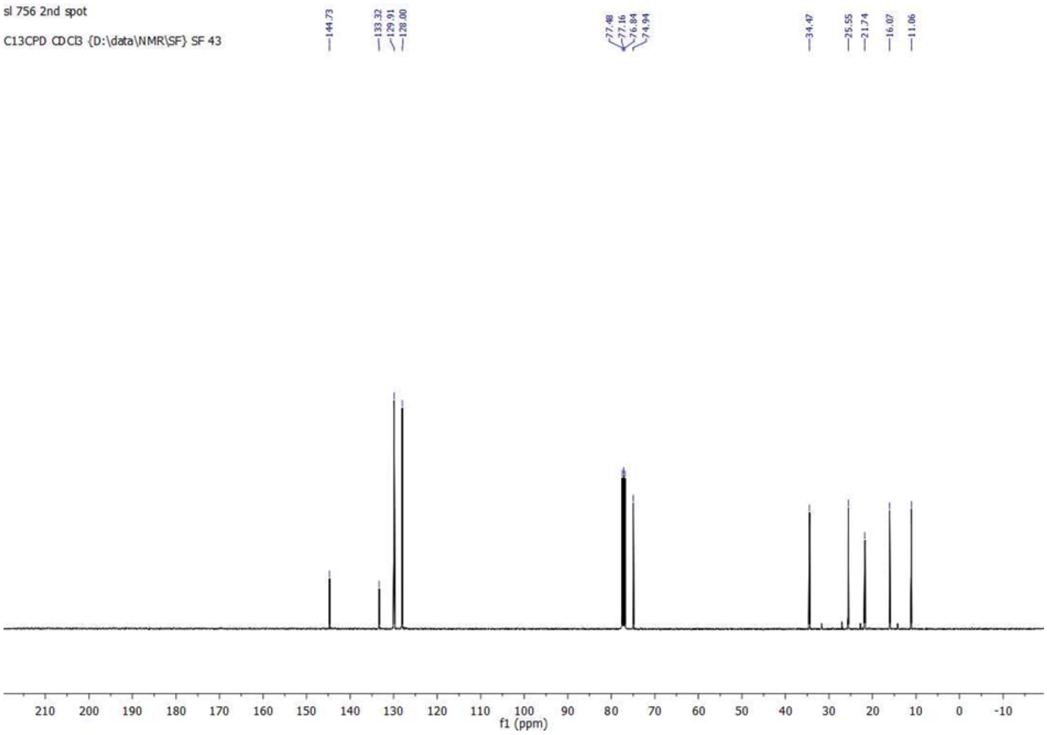

Figure S2. ^{13}C NMR spectrum (101 MHz, $CDCl_3$ at 298 K) of **5**.

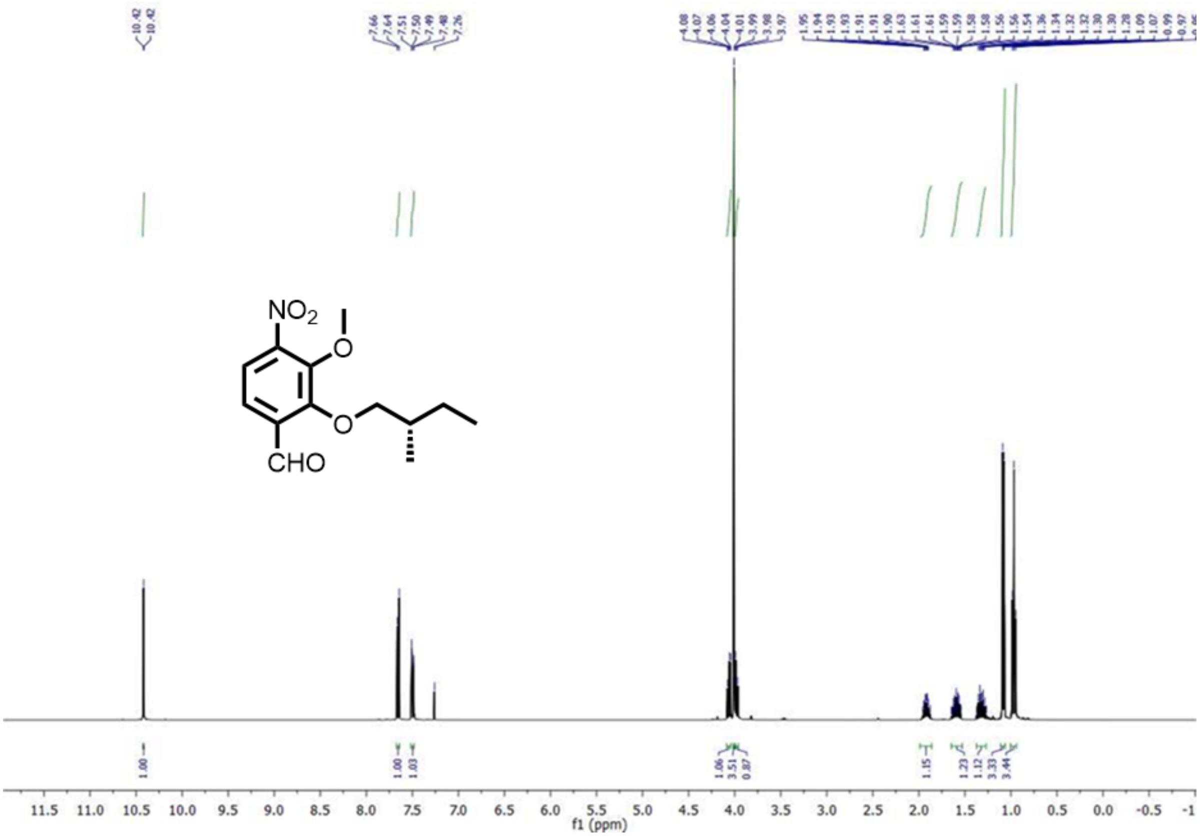

Figure S3. ^{1}H NMR spectrum (400 MHz, $CDCl_3$ at 298 K) of **7**.

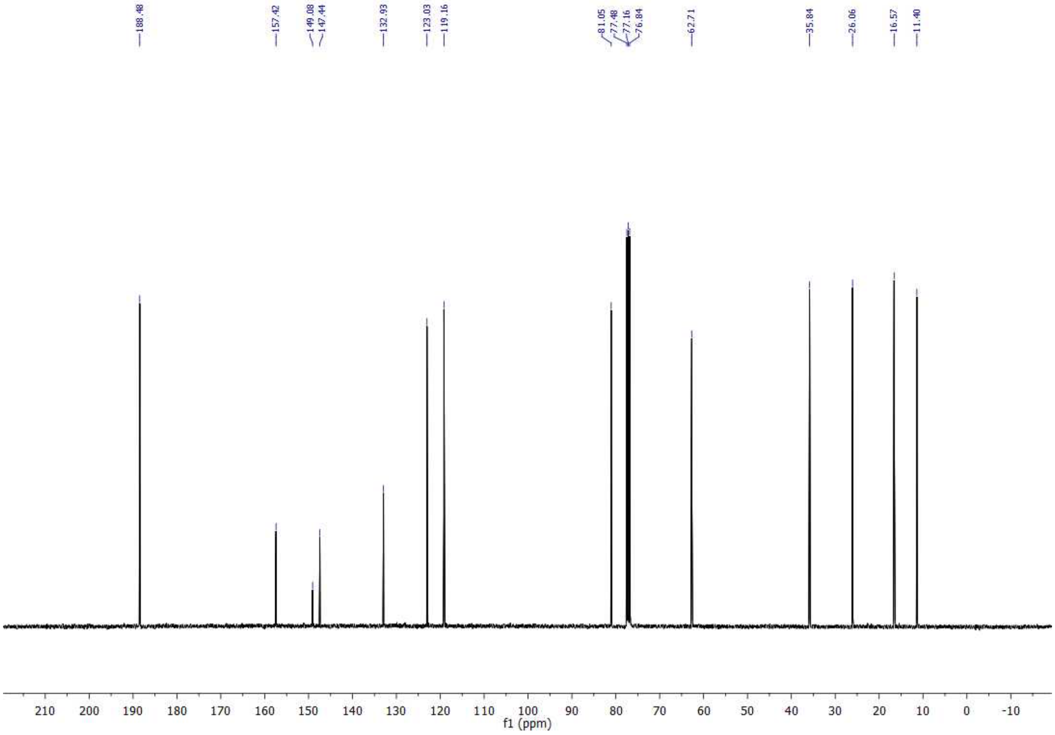

Figure S4. ^{13}C NMR spectrum (101 MHz, $CDCl_3$ at 298 K) of **7**.

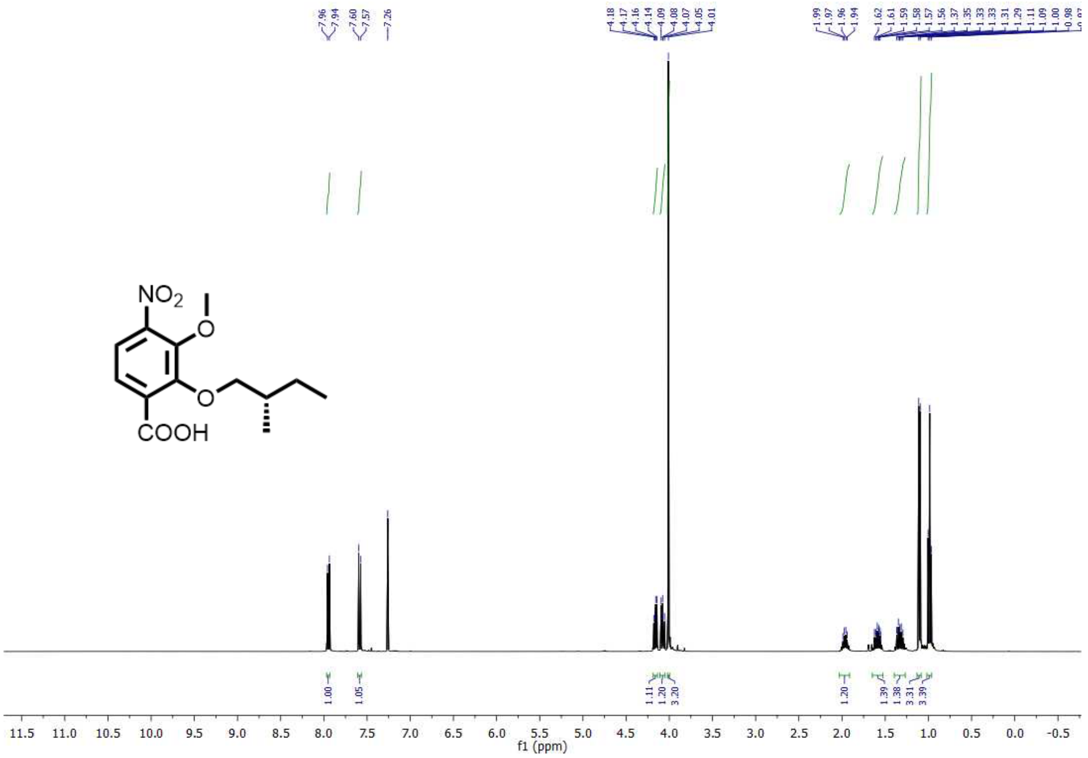

Figure S5. ^{1}H NMR spectrum (400 MHz, $CDCl_3$ at 298 K) of **8**.

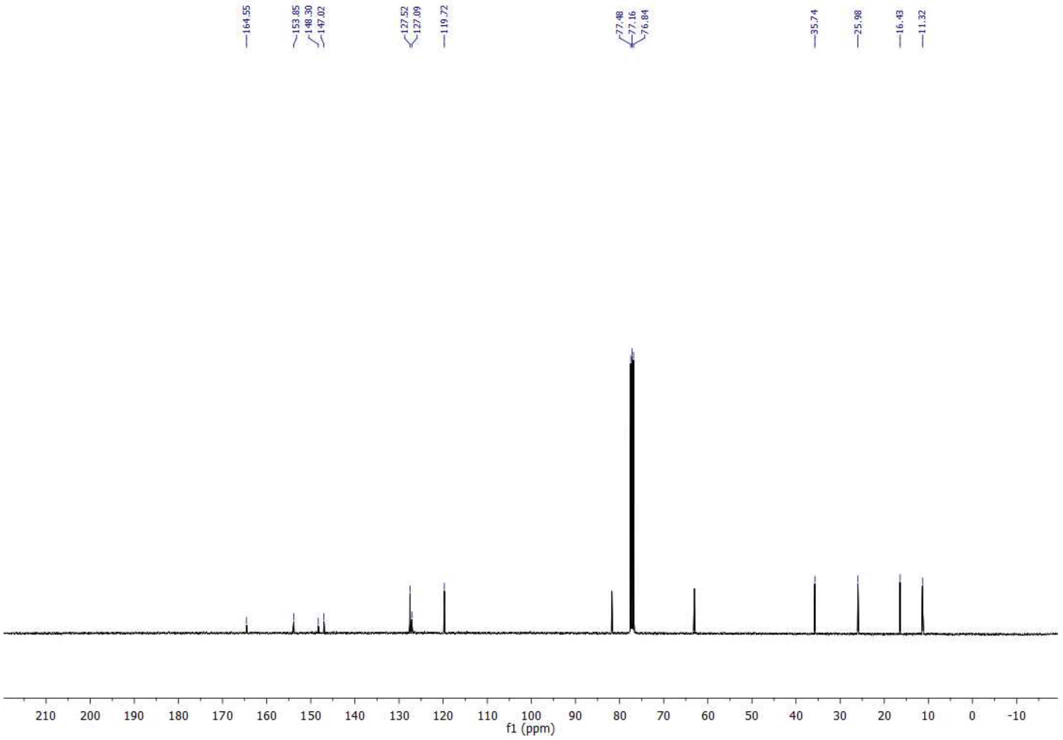

Figure S6. ^{13}C NMR spectrum (101 MHz, $CDCl_3$ at 298 K) of **8**.

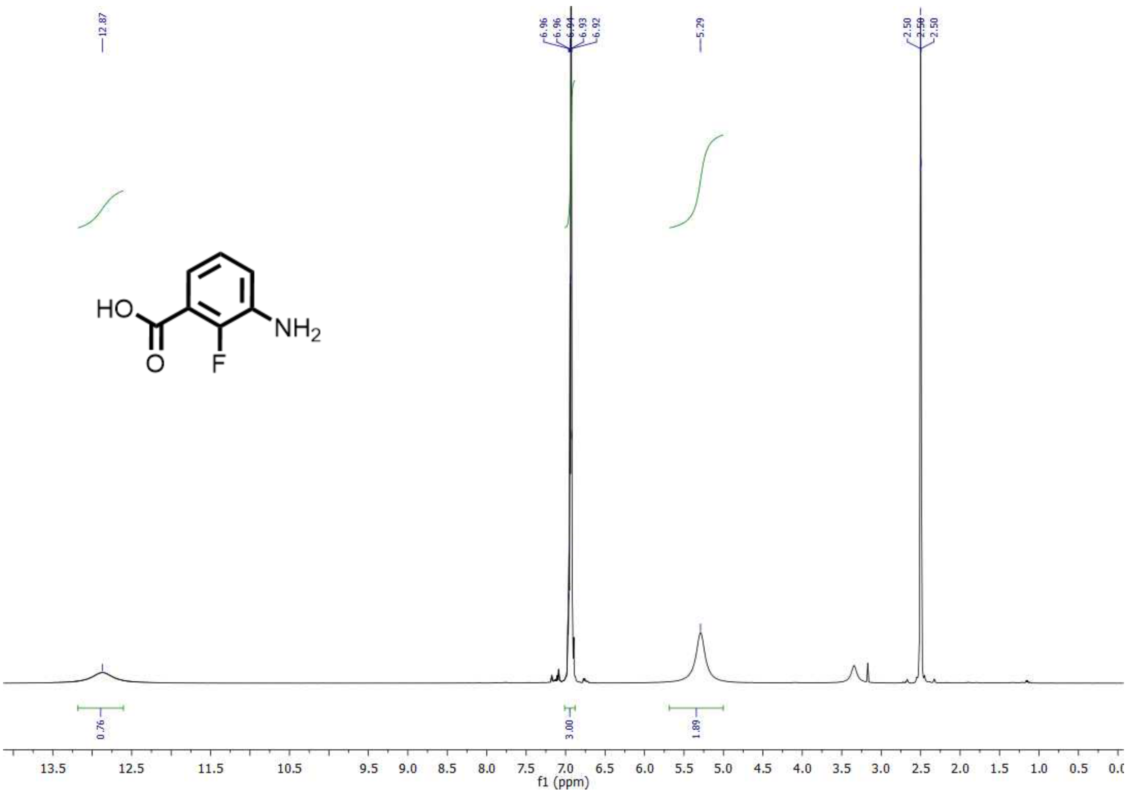

Figure S7. ¹H NMR spectrum (400 MHz, DMSO-d_6 at 298K) of **9**.

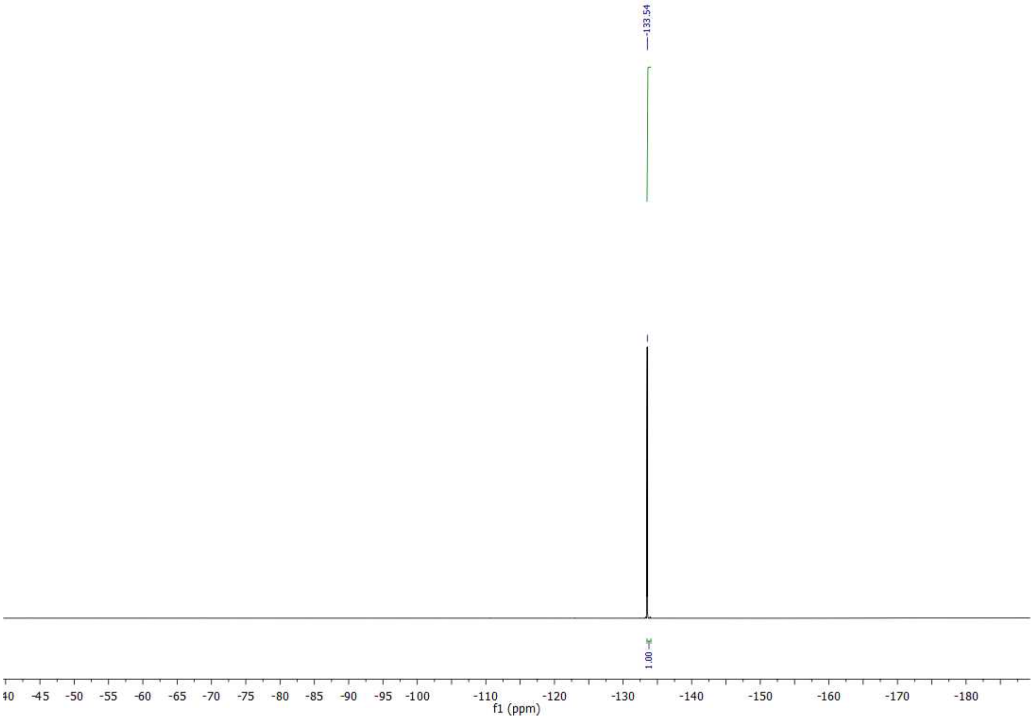

Figure S8. ¹⁹F NMR spectra (377 MHz, DMSO-d_6 at 298K) of **9**.

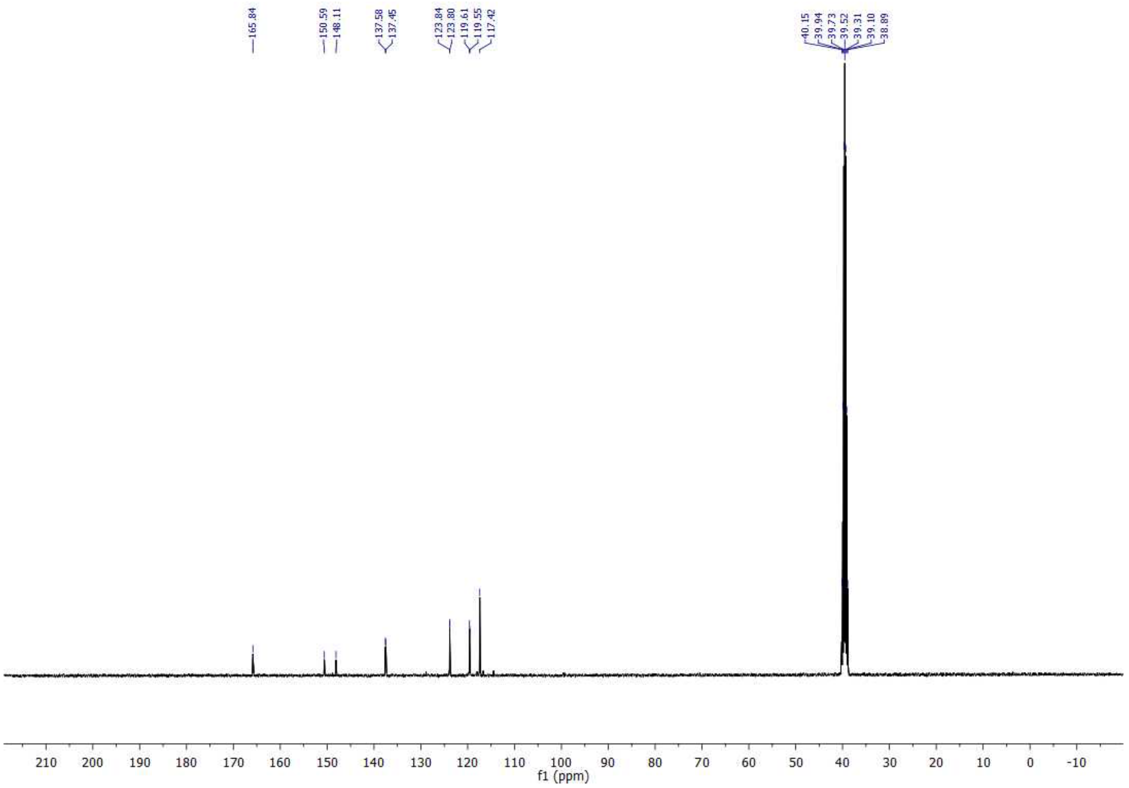

Figure S9. ^{13}C NMR spectrum (101 MHz, DMSO-d_6 at 298K) of **9**.

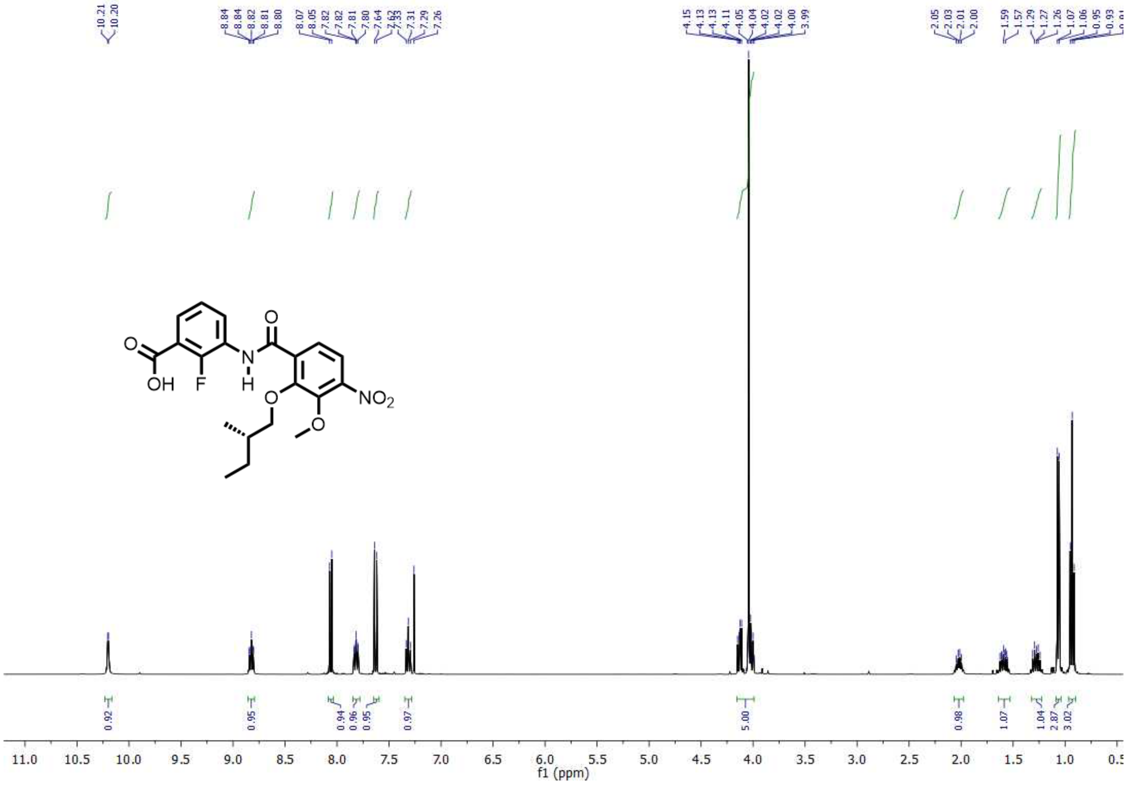

Figure S10. ^{1}H NMR spectrum (400 MHz, $CDCl_3$ at 298 K) of **10**.

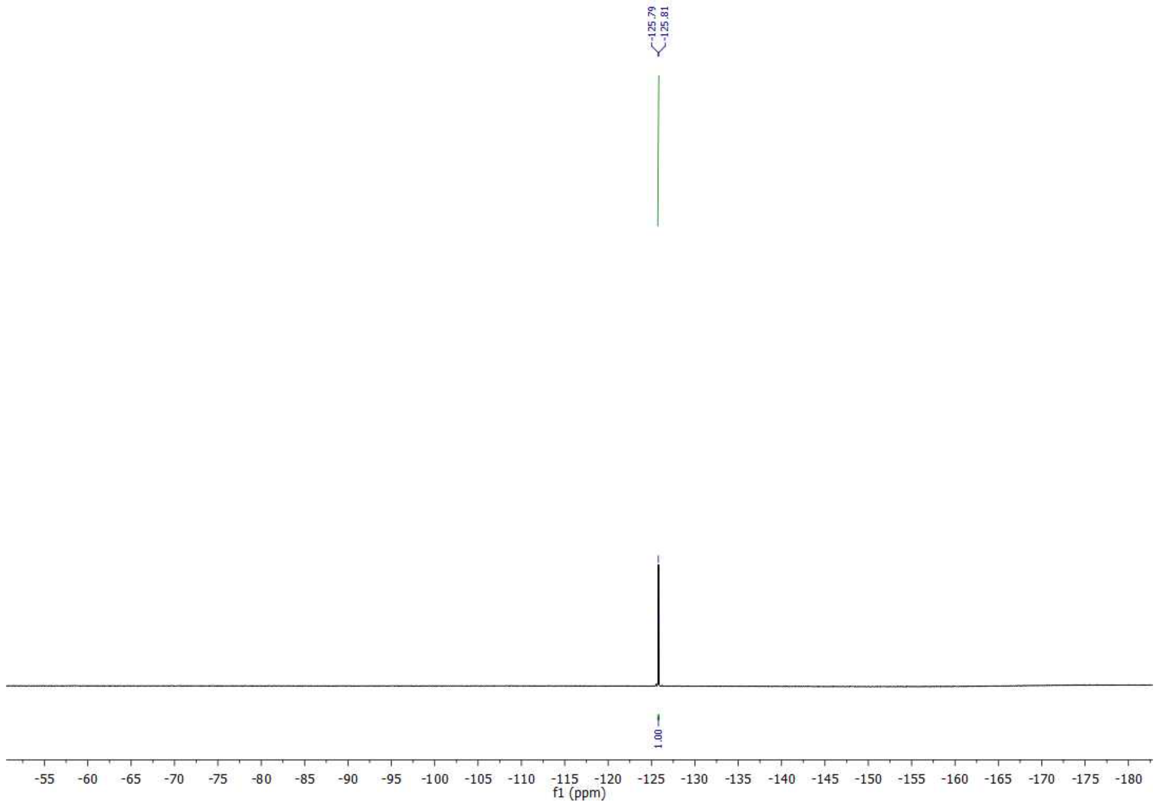

Figure S11. ^{19}F NMR spectra (377 MHz, $CDCl_3$ at 298 K of **10**.

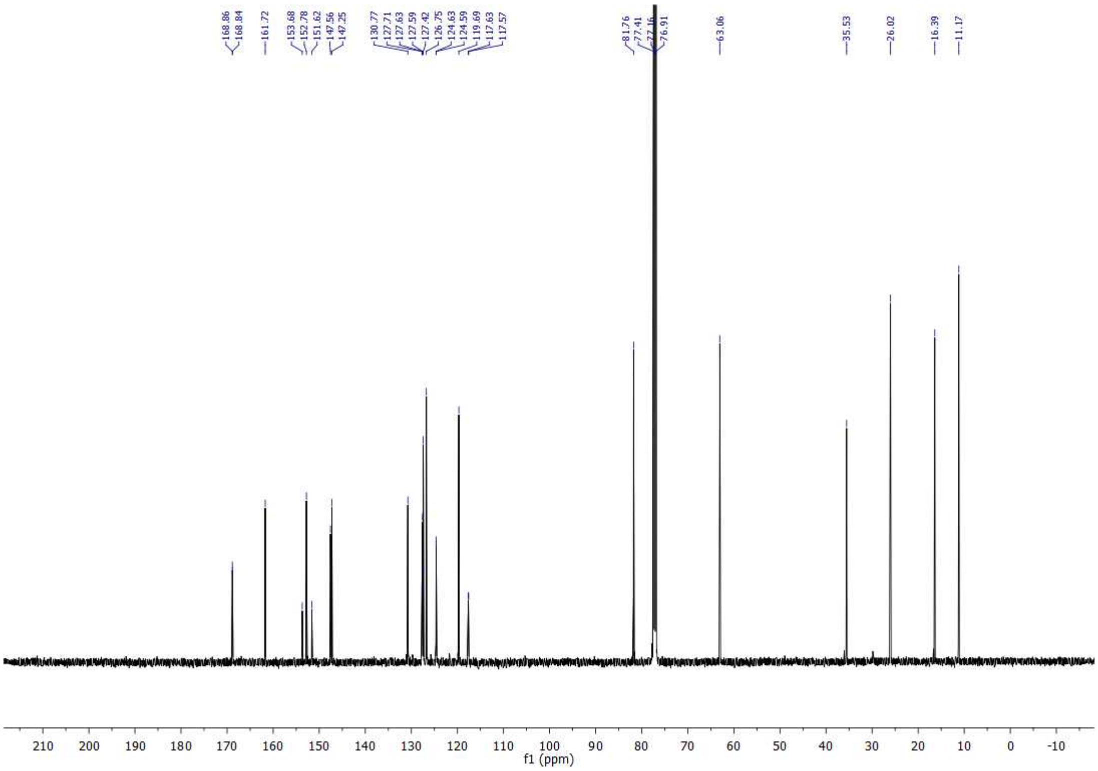

Figure S12. ^{13}C NMR spectrum (101 MHz, $CDCl_3$ at 298 K) of **10**.

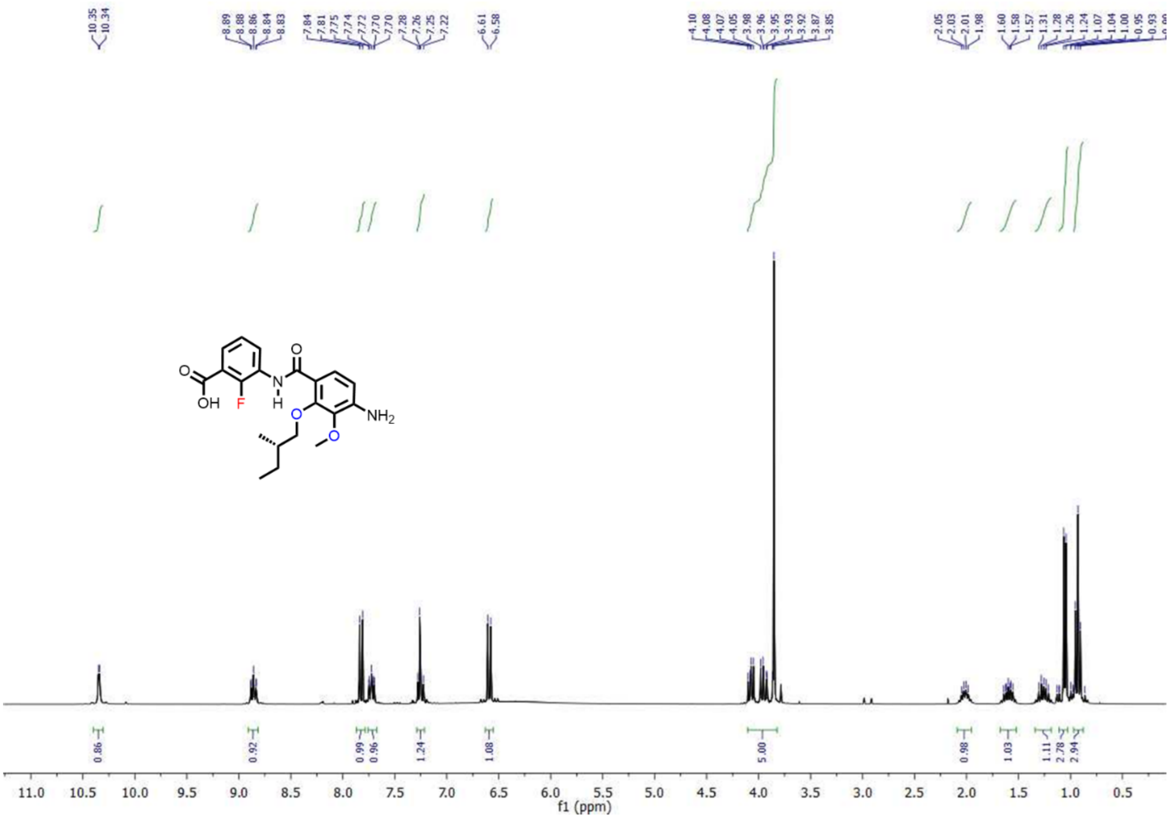

Figure S13. ^{1}H NMR spectrum (400 MHz, $CDCl_3$ at 298 K) of **2**.

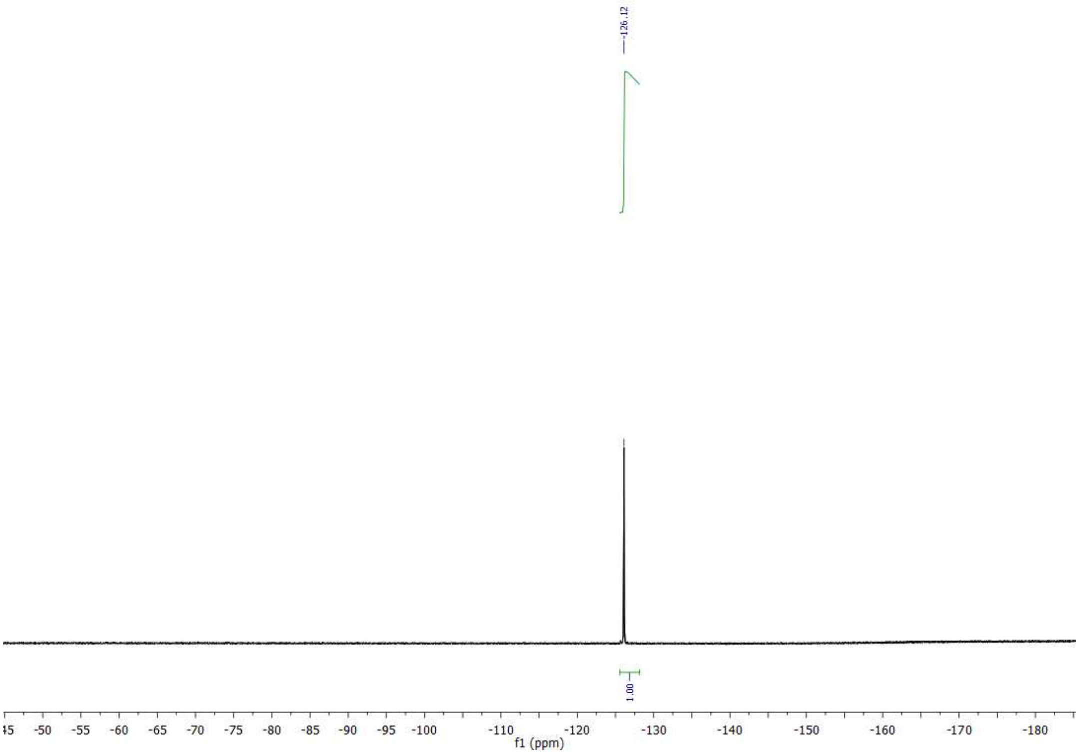

Figure S14. ^{19}F NMR spectra (377 MHz, $CDCl_3$ at 298 K) of **2**.

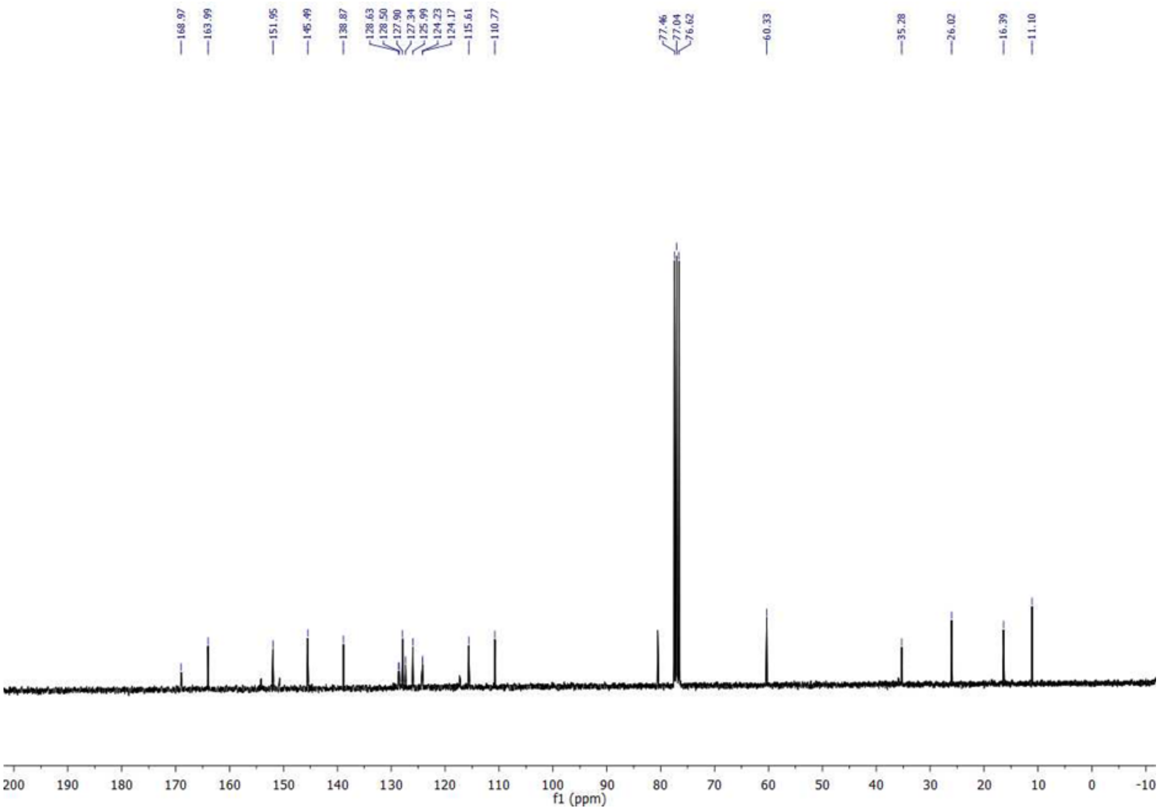

Figure S15. ^{13}C NMR spectrum (101 MHz, $CDCl_3$ at 298 K) of **2**.

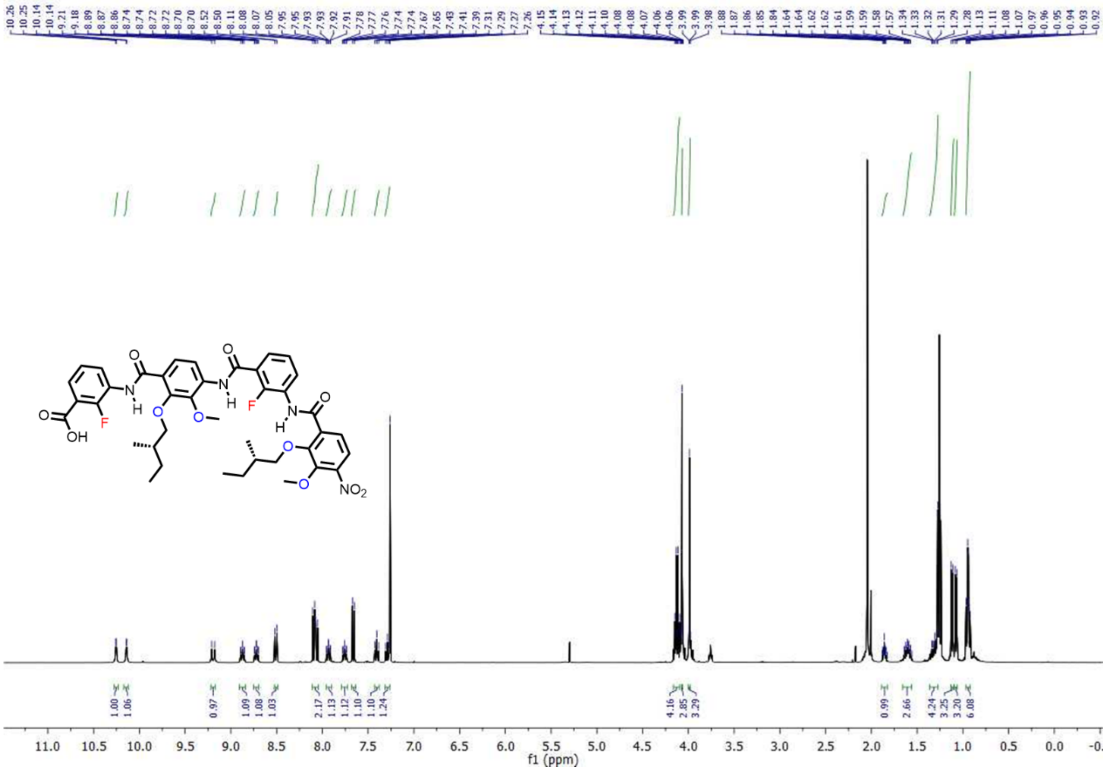

Figure S16. ^{1}H NMR spectrum (400 MHz, $CDCl_3$ at 298 K) of **11**.

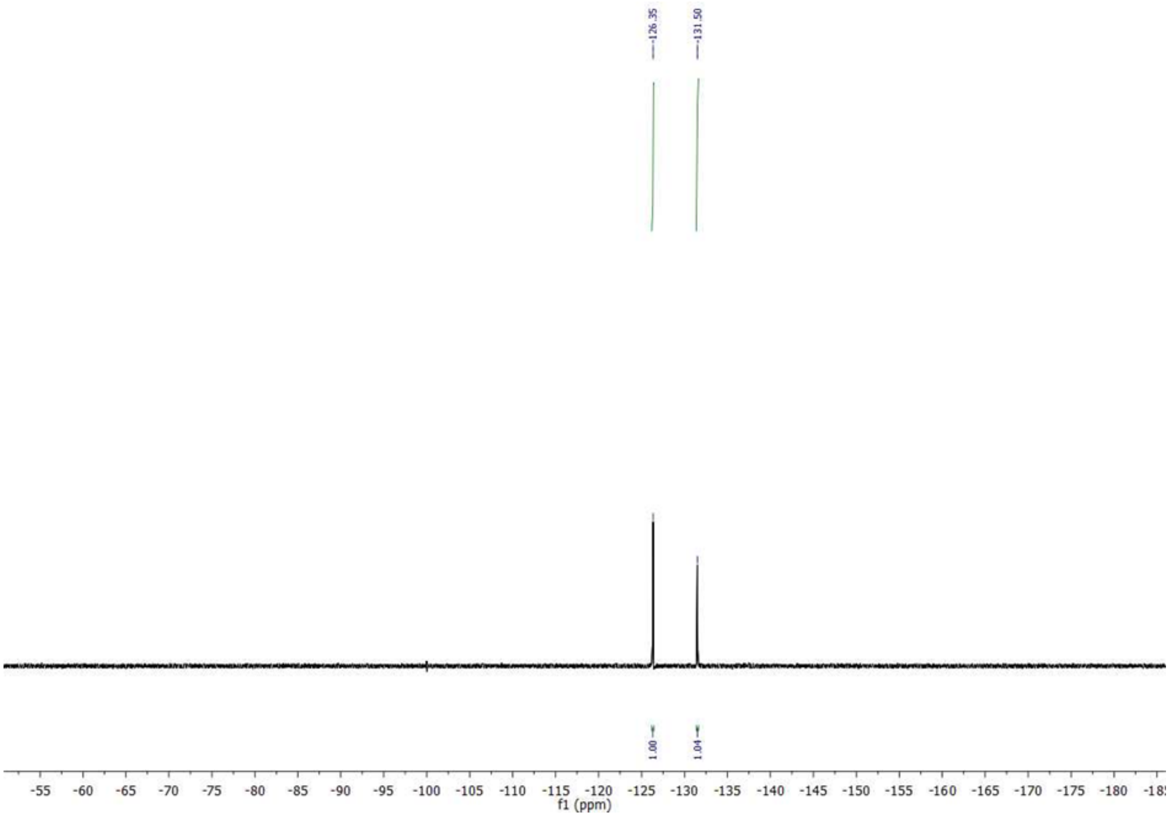

Figure S17. ^{19}F NMR spectra (377 MHz, $CDCl_3$ at 298 K) of **11**.

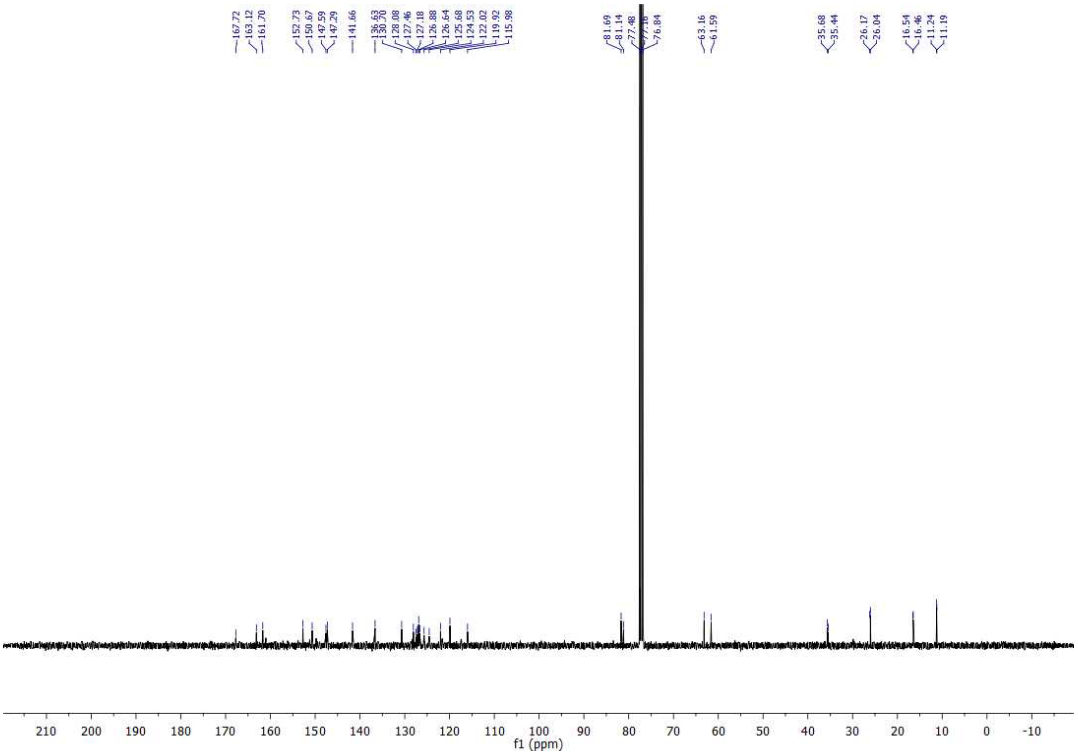

Figure S18. ^{13}C NMR spectrum (101 MHz, $CDCl_3$ at 298 K) of **11**.

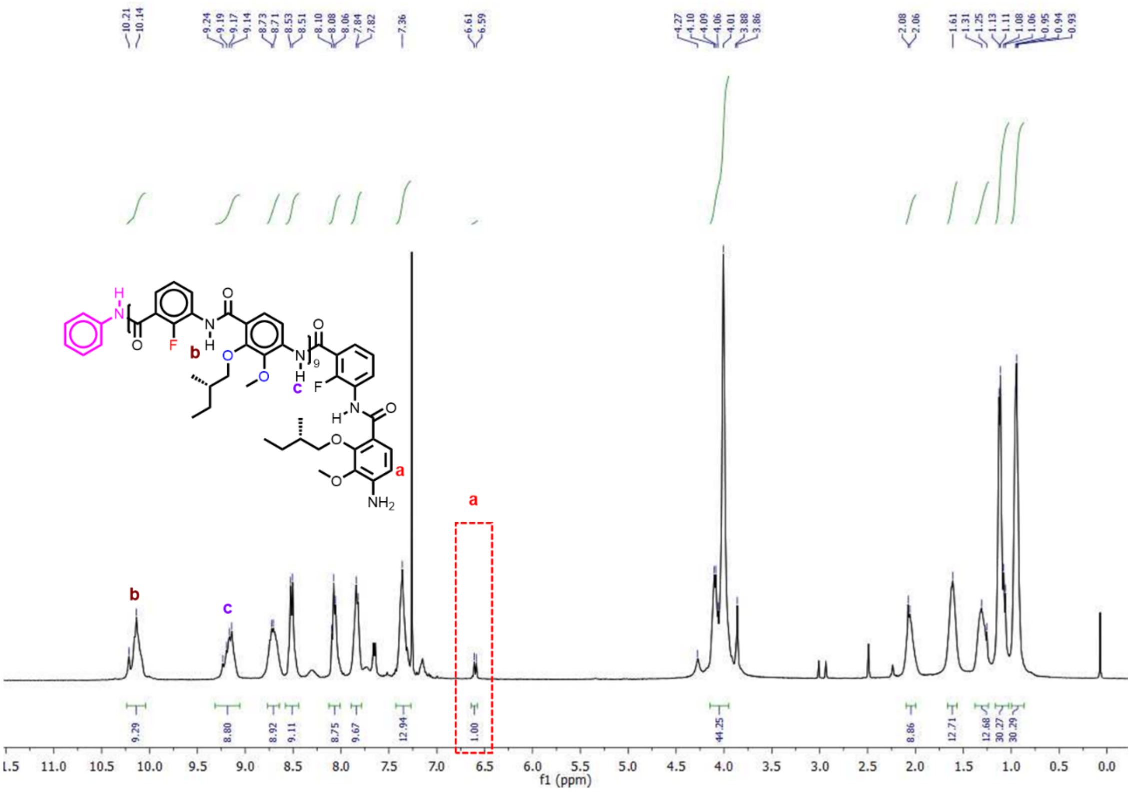

Figure S19. ^{1}H NMR spectrum (400 MHz, $CDCl_3$ at 298 K) **Poly-10mer**.

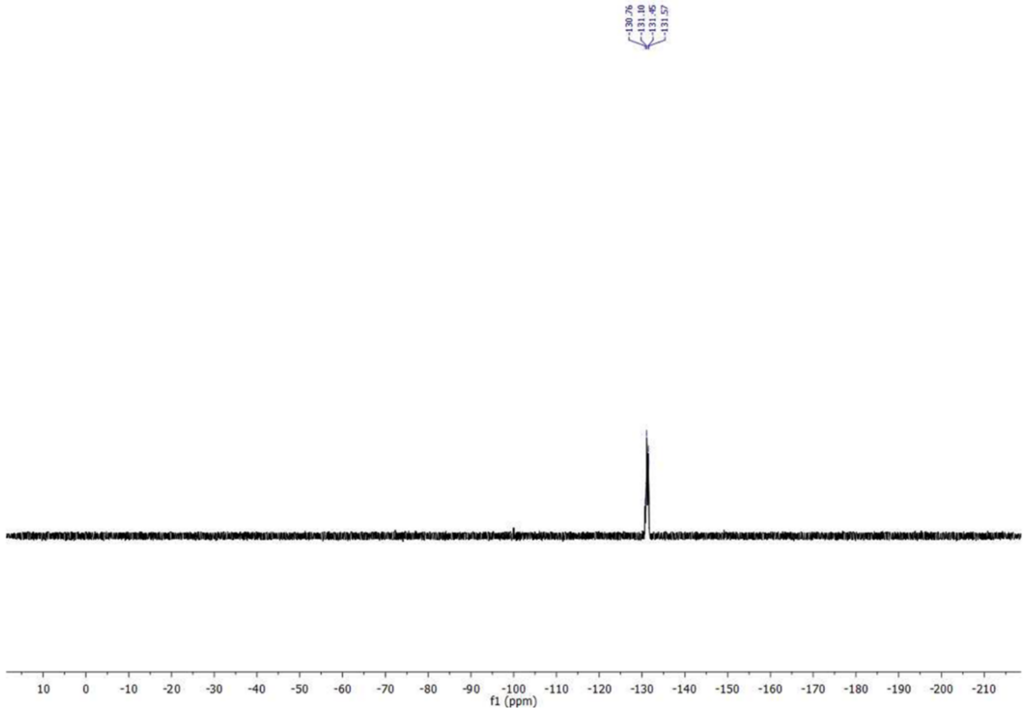

Figure S20. ^{19}F NMR spectra (377 MHz, $CDCl_3$ at 298 Kd) of **Poly-10mer**.

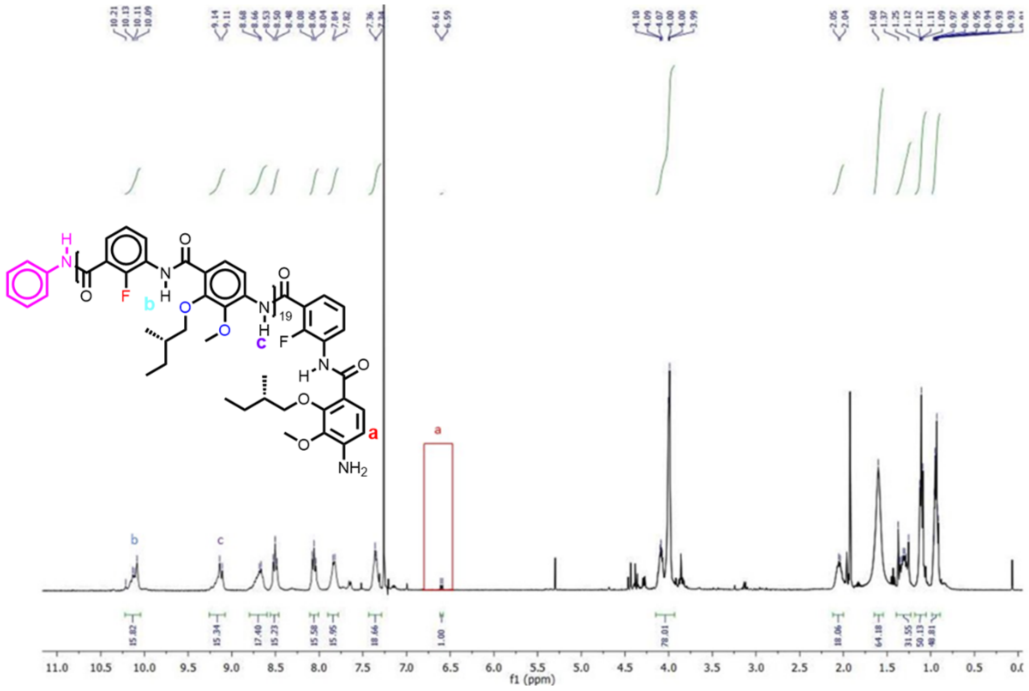

Figure S21. ^{1}H NMR spectrum (400 MHz, $CDCl_3$ at 298 K) **Poly-20mer**.

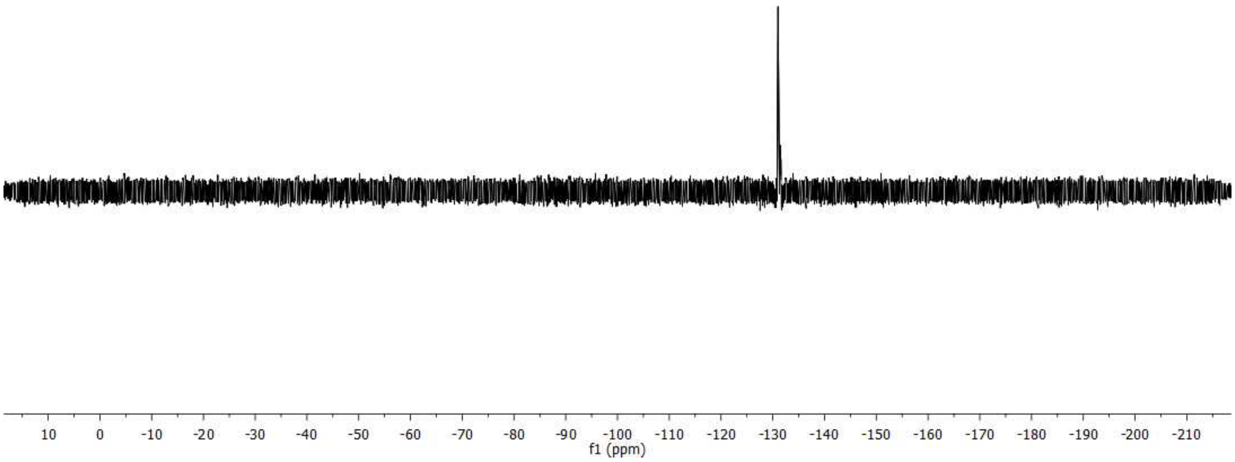

Figure S22. ^{19}F NMR spectra (377 MHz, $CDCl_3$ at 298 K) of **Poly-20mer**.

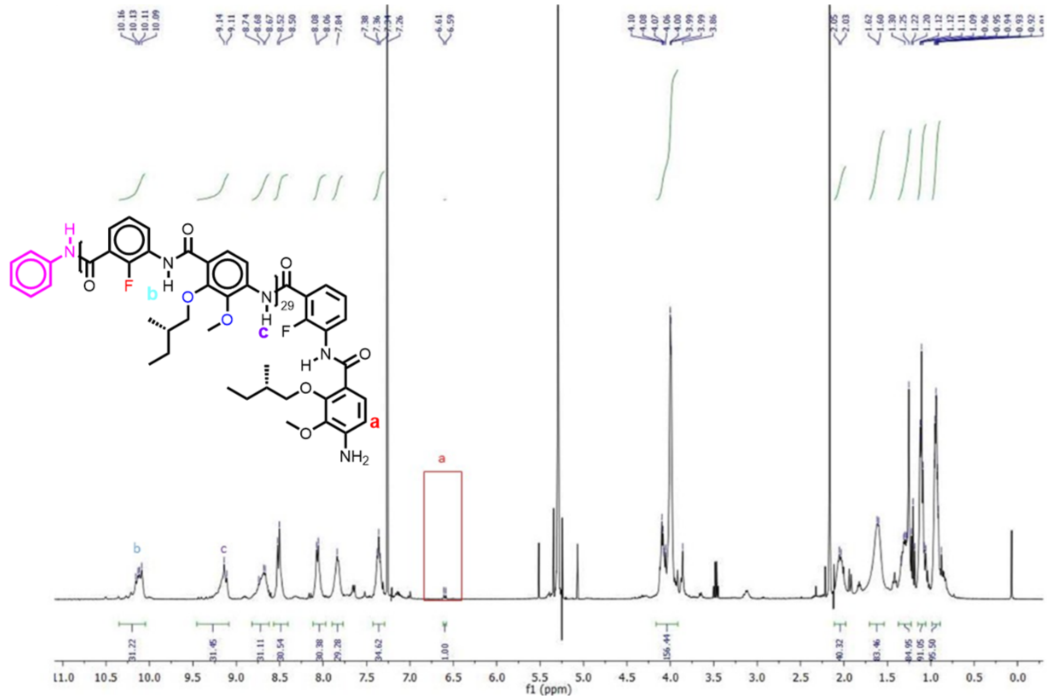

Figure S23. ^{1}H NMR spectrum (400 MHz, $CDCl_3$ at 298 K) **Poly-30mer**.

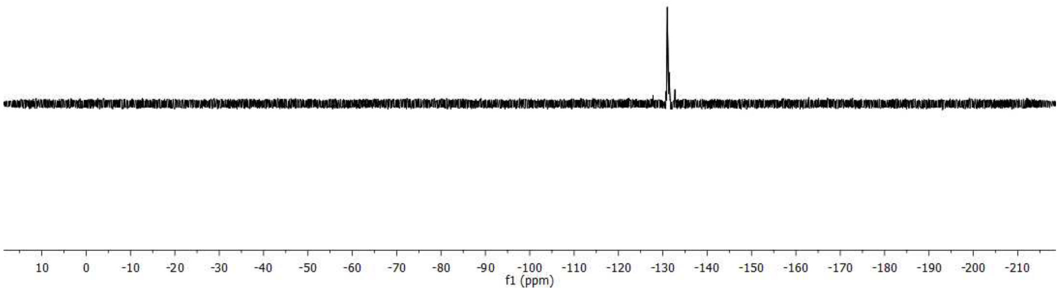

Figure S24. ^{19}F NMR spectra (377 MHz, $CDCl_3$ at 298 K) of **Poly-30mer**.

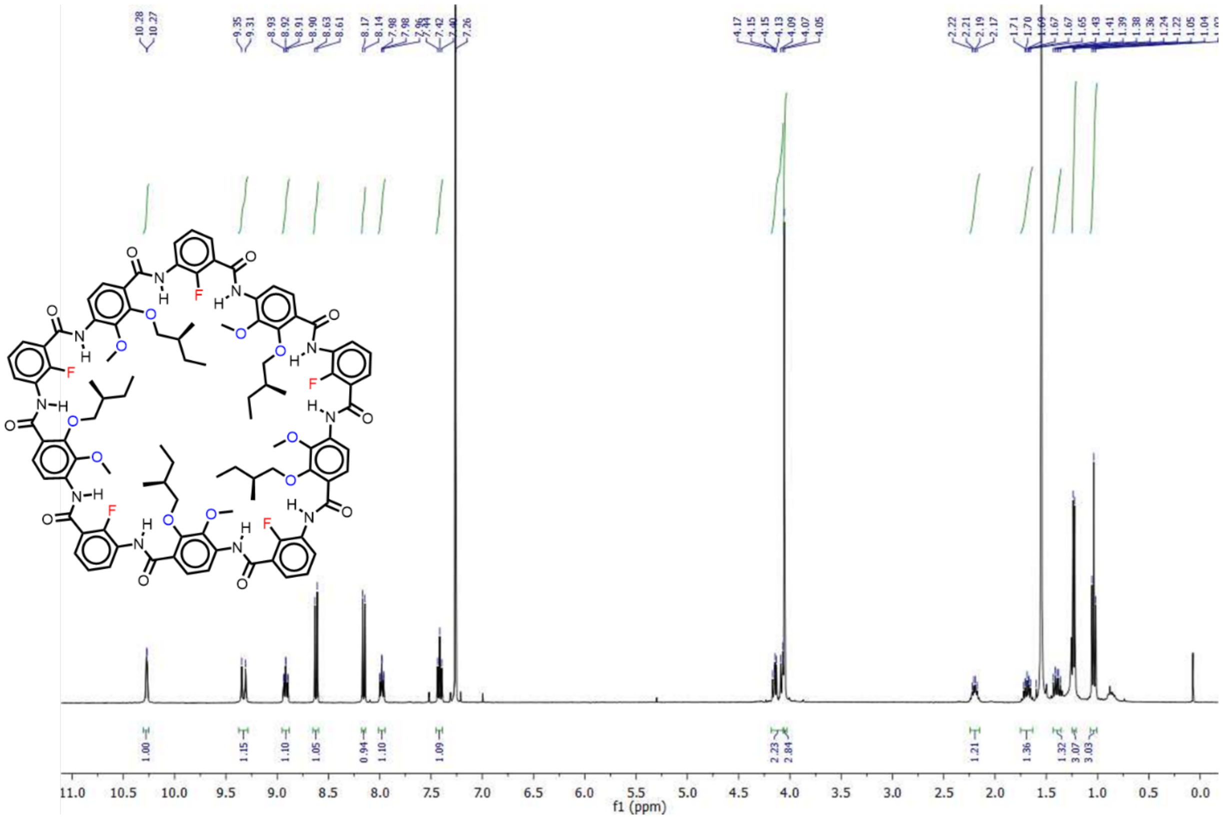

Figure S25. ^{1}H NMR spectrum (400 MHz, $CDCl_3$ at 298 K) of **4**.

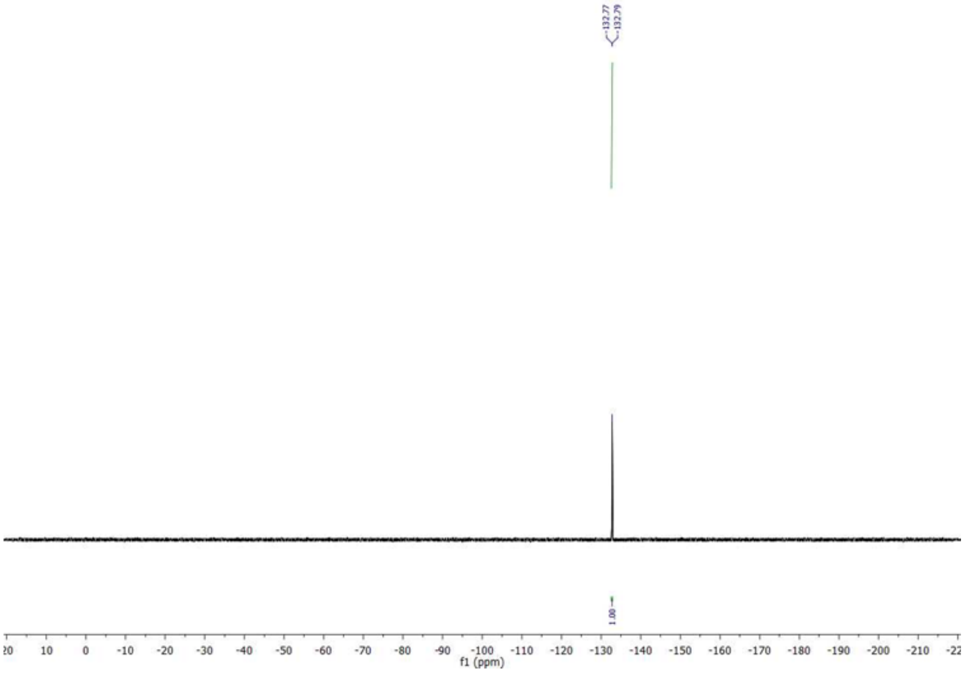

Figure S26. ^{19}F NMR spectra (377 MHz, $CDCl_3$ at 298 K) of **4**.

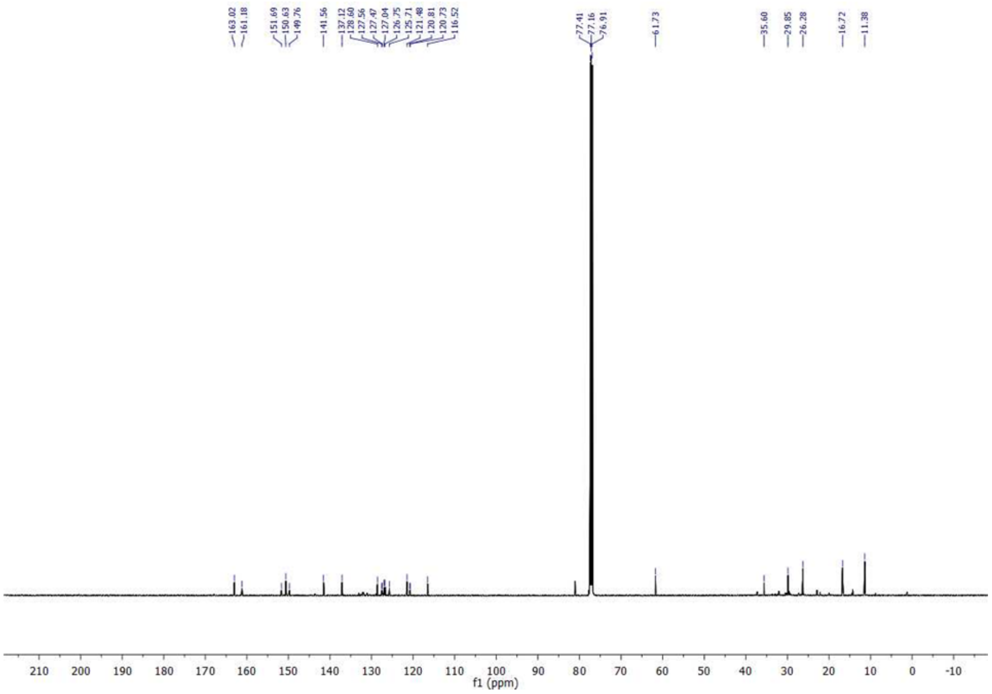

Figure S27. ^{13}C NMR spectrum (101 MHz, $CDCl_3$ at 298 K) of **4**.

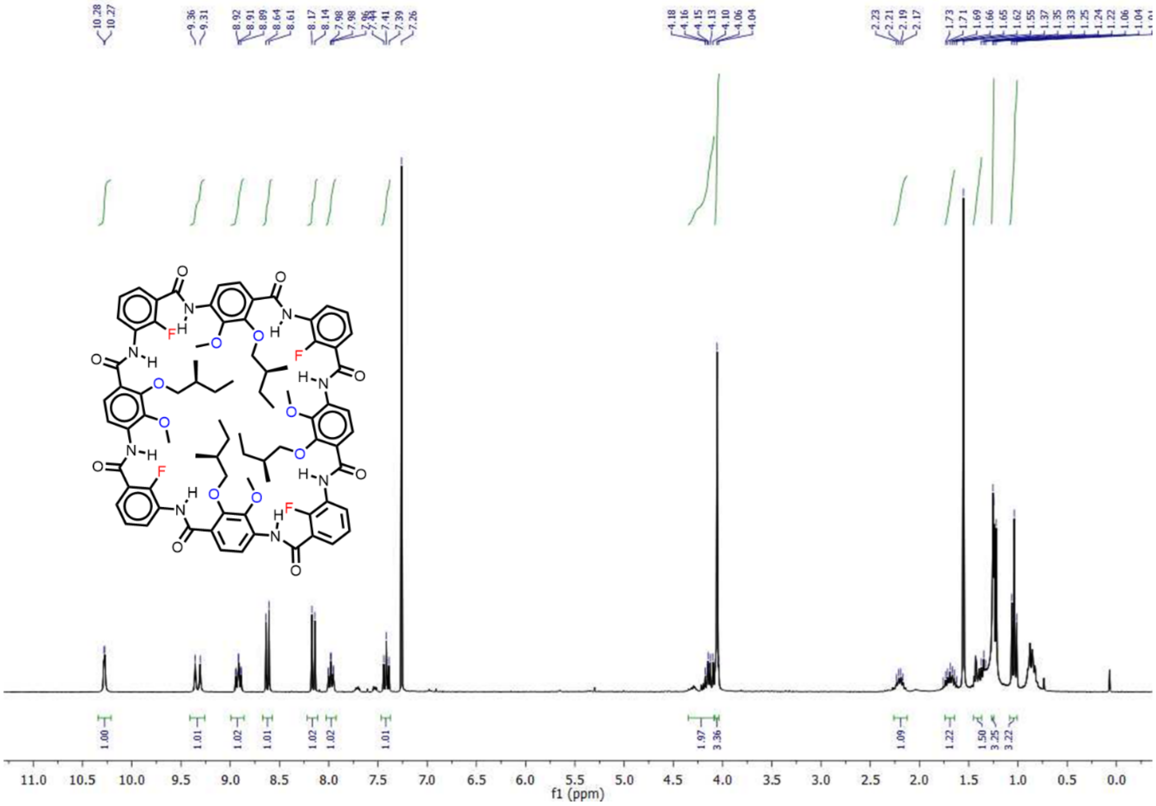

Figure S28. ^{1}H NMR spectrum (400 MHz, $CDCl_3$ at 298 K) of **3**.

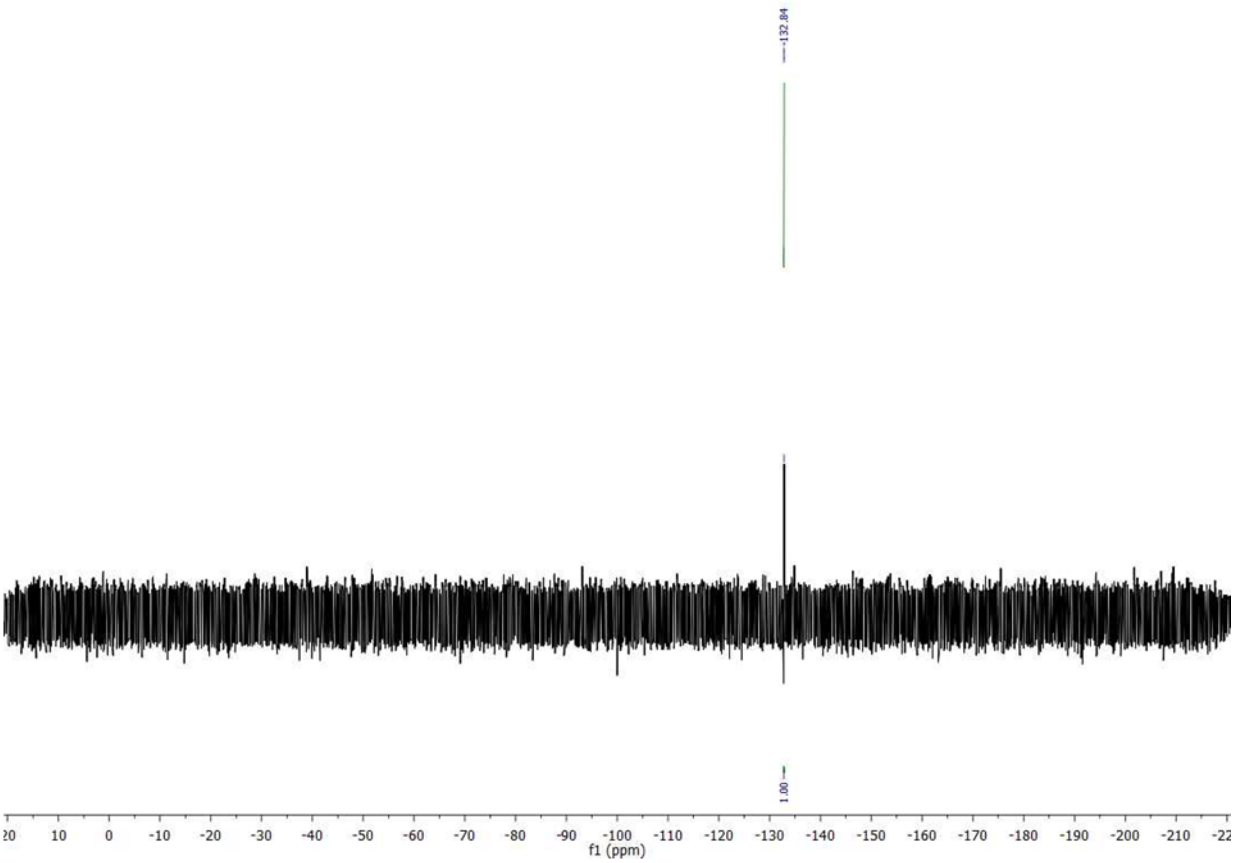

Figure S29. ^{19}F NMR spectra (377 MHz, $CDCl_3$ at 298 K) of **3**.

5. Polymer characterization data

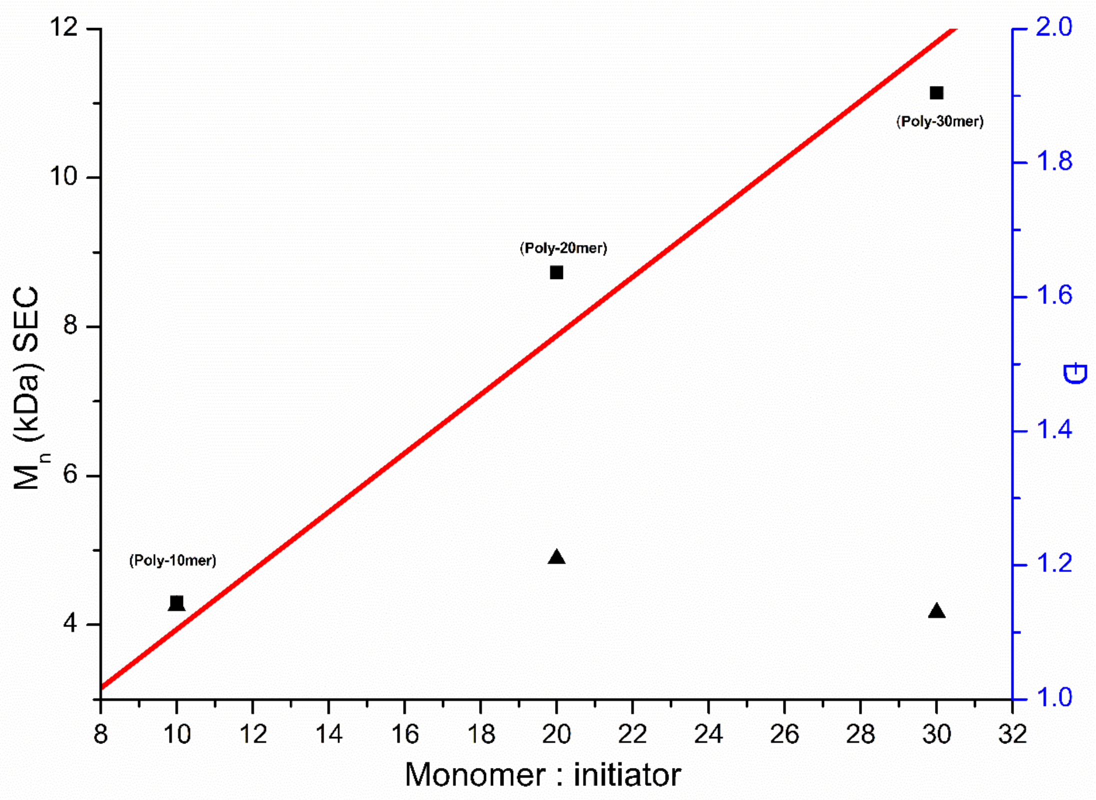

Figure S30. Linear relationship of number average molecular weights (**M_n**) vs monomer: initiator ratio in polymers **Poly-10mer** to **Poly-30mer**.

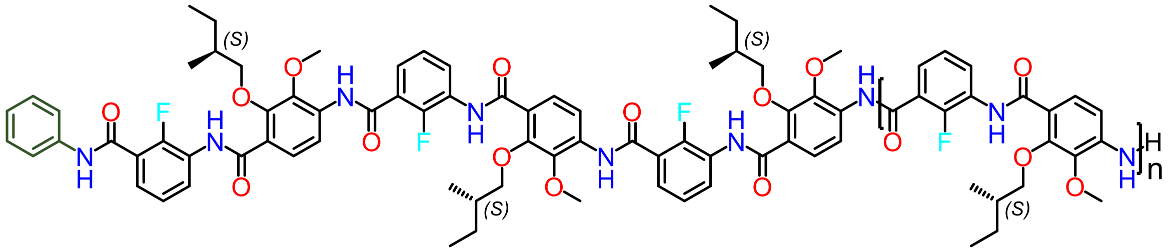

Poly-10mer; n = 7

Poly-20mer; n = 13

Poly-30mer; n = 23

Figure S31. Chemical structures of polymers **Poly-10mer** to **Poly-30mer**.

Table S1. Number average molecular weights (M_n) and polydispersity indexes (Đ = M_w/M_n) of the polymers synthesized from monomer **7** and initiator (aniline).

Polymer	M/I	Mn-theo. (kDa)	$M_{n\text{-obser.}}$ SEC ($CHCl_3$)(kDa)	$M_{n\text{-obser.}}$ ^{1}H-NMR(kDa)	Đ
**Poly-10mer**	**10**	3.8	4.3	3.5	1.14
**Poly-10mer**	**20**	7.5	8.7	6.1	1.21
**Poly-10mer**	**30**	11.2	11.1	11.2	1.13

Preparative HPLC data

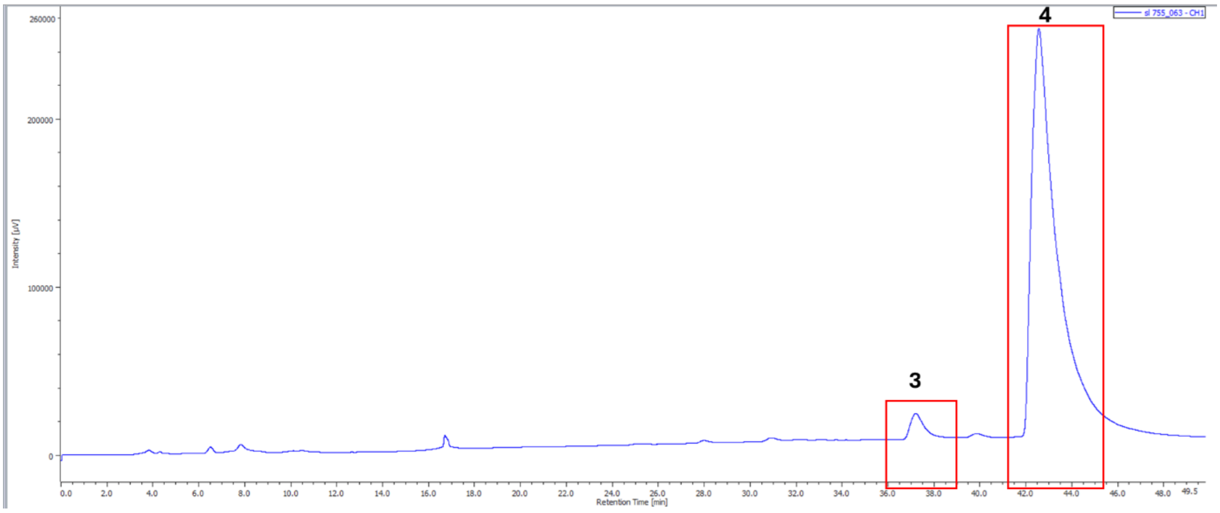

Figure S32. Preparative HPLC of the reaction mixture of monomer **2** using **PHOS3** (**1**)without an initiator at room temperature. Separation was done using 10-20 % diethyl ether in dichloromethane to give pure **4** and **3** as white solids.

MALDI-ToF spectra

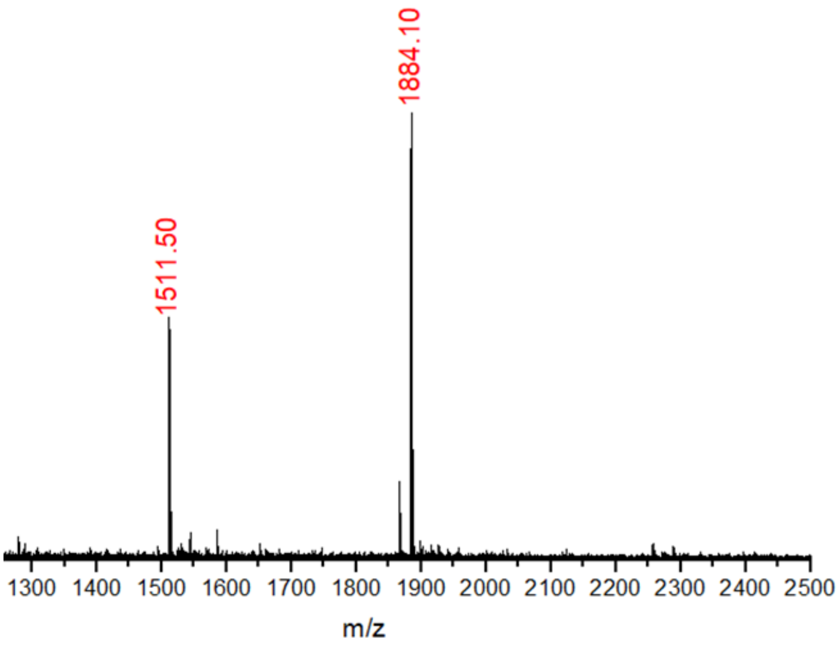

Figure S33. MALDI-ToF mass spectrum (DCTB, NaTFA) of a reaction mixture of monomer **2** using **1** without an initiator at room temperature.

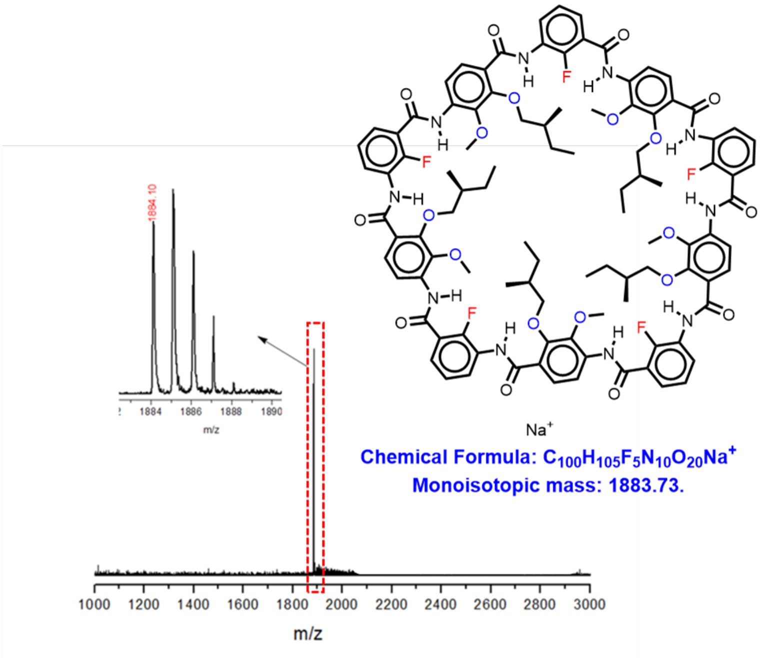

Figure S34. MALDI-ToF mass spectrum (DCTB, NaTFA) of **4**.

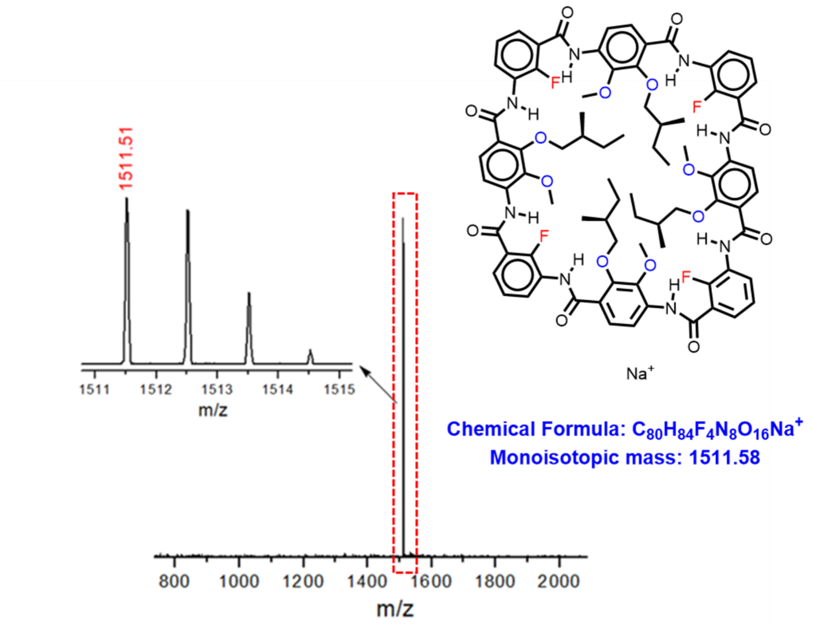

Figure S35. MALDI-ToF mass spectrum (DCTB, NaTFA) of **3**.

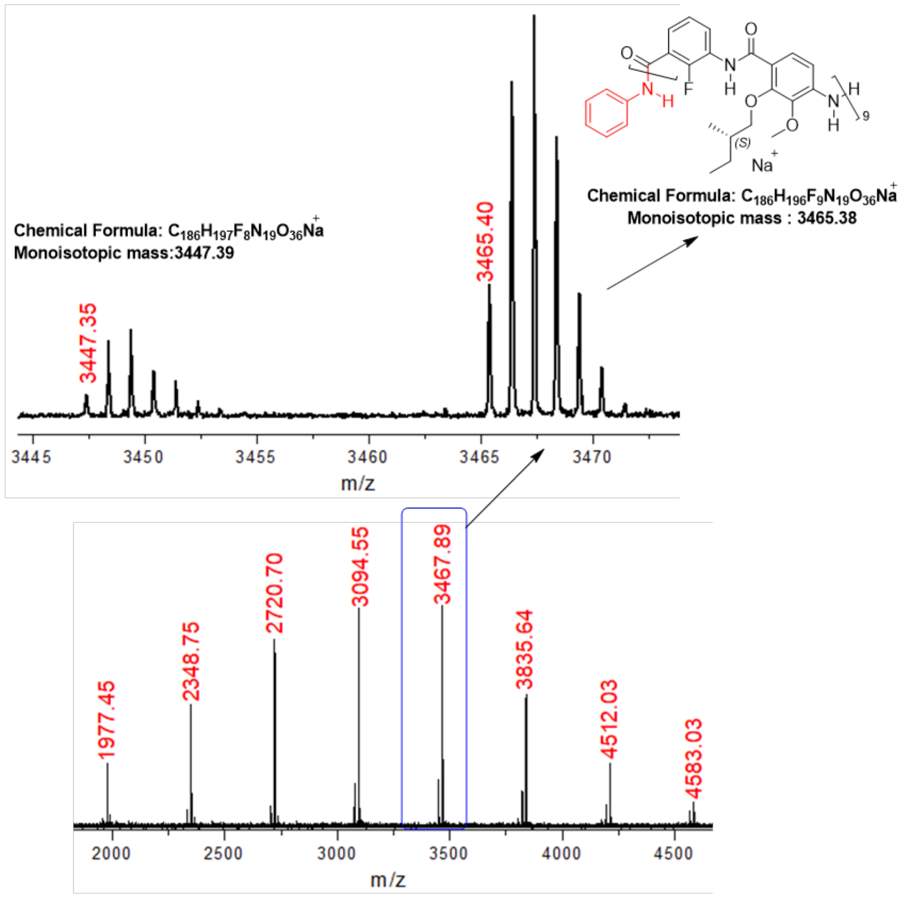

Figure S36. MALDI-ToF mass spectrum (DCTB, NaTFA) of **Poly-10mer**.

The minor mass distribution of **Poly-10mer** matched a structure in which one fluorine atom was replaced by hydrogen in only one of the repeating units (Figure S36). We believe this defluorination occurs during the MALDI-ToF MS measurement rather than during polymerization. To confirm this assumption, we carried out MALDI-ToF analysis on dimer **11**, which was fully characterized by ^{1}H NMR (SI, Figure S17), ^{13}C-NMR, ^{19}F-NMR spectroscopy (SI, Figure S18), and HRMS (SI, Figure S54) and no defluorination was observed. Interestingly, **11** also showed two mass distributions (SI, Figure S37) in the MALDI-ToF mass spectrum: one being **11** as a sodium cation adduct and the other the defluorinated product (as a sodium cation adduct). This experiment confirmed that the mono-defluorination is most likely a result of the MALDI MS analysis.

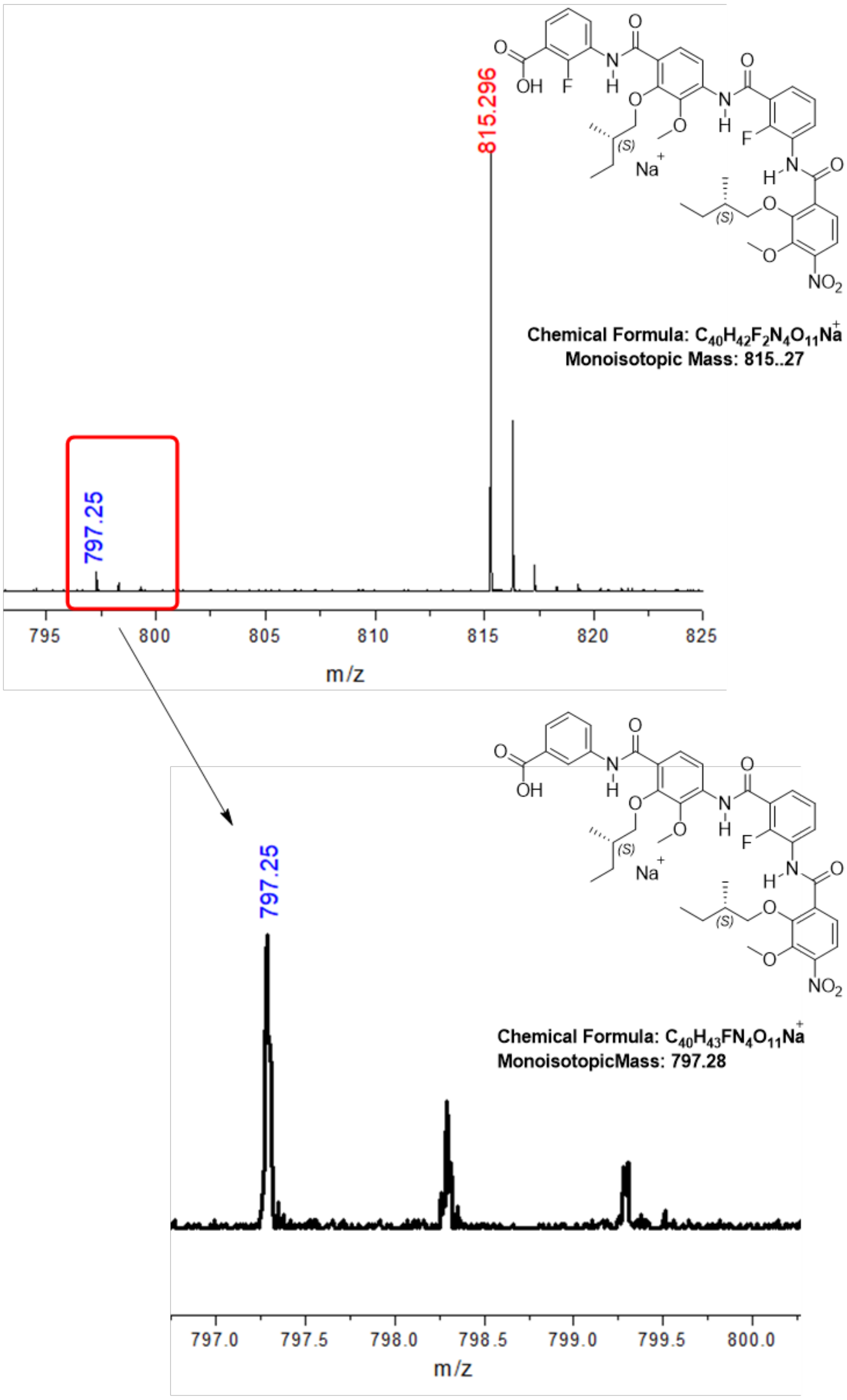

Figure S37. MALDI-ToF mass of spectrum (DCTB, NaTFA) of **11**.

SEC elugrams of polymers

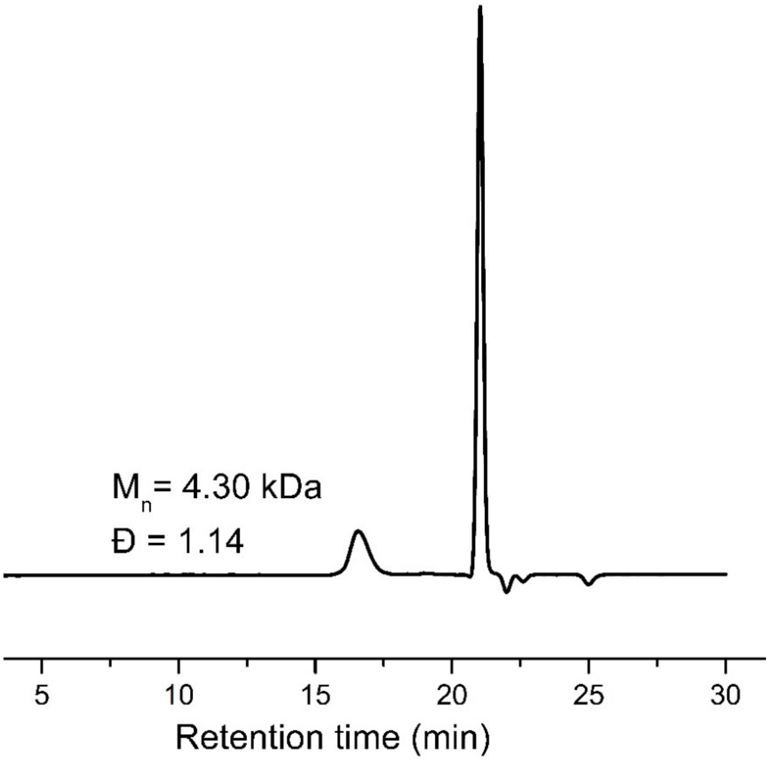

Figure S38. SEC ($CHCl_3$) of **Poly-10mer**.

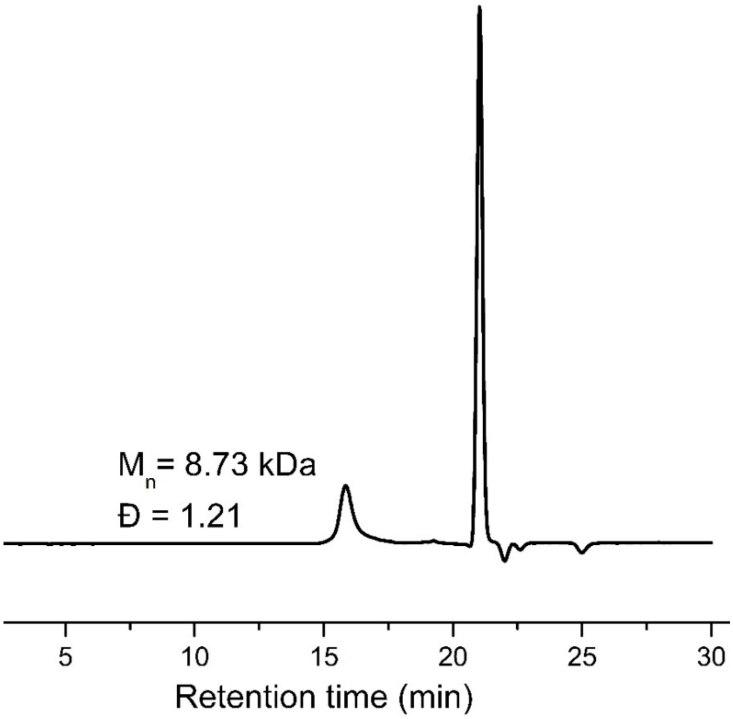

Figure S39. SEC ($CHCl_3$) of **Poly-20mer**.

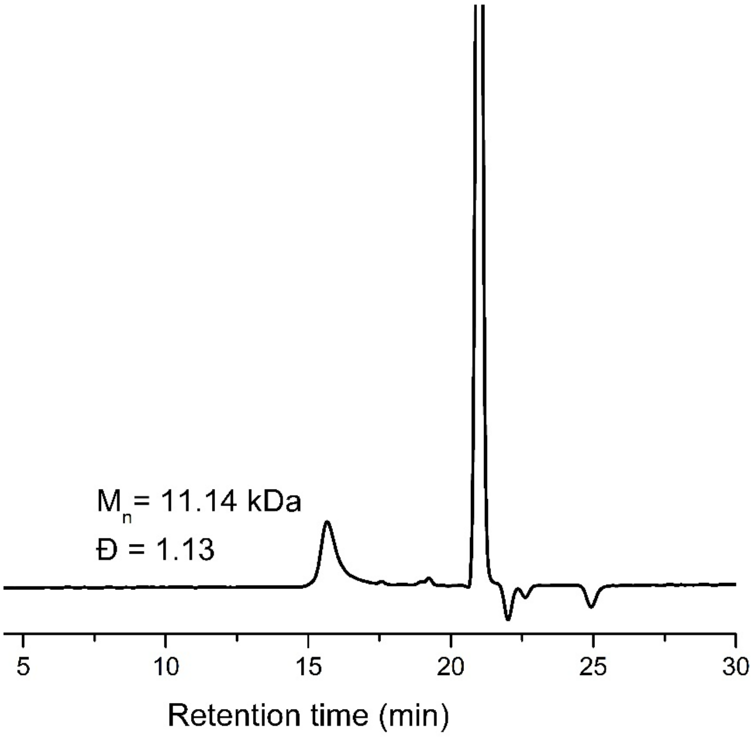

Figure S40. SEC ($CHCl_3$) of **Poly-30mer**.

CD spectra

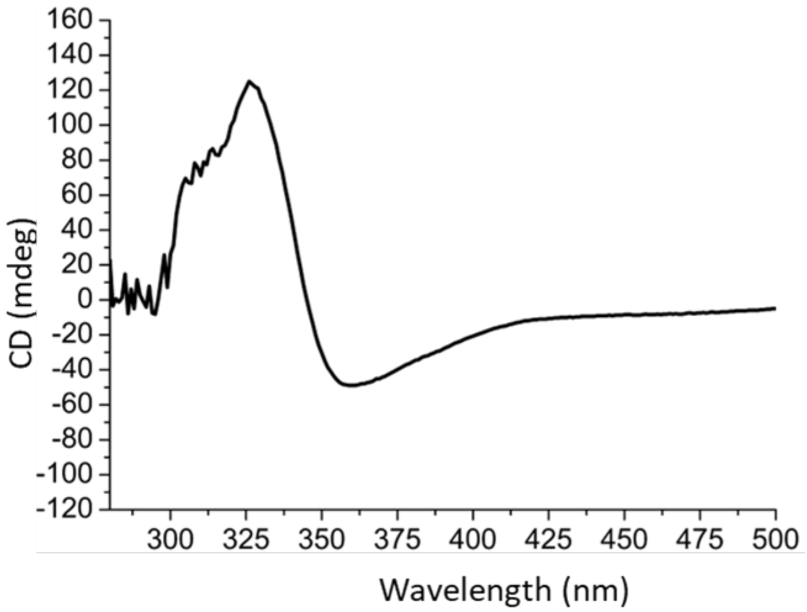

***Figure S41*.** CD spectra of a **Poly-10mer** recorded in toluene at 298 K, concentration 100 mg/L. **Poly-10mer** was dissolved in 2-3 drops of chloroform and then diluted with the required amount of toluene at room temperature.

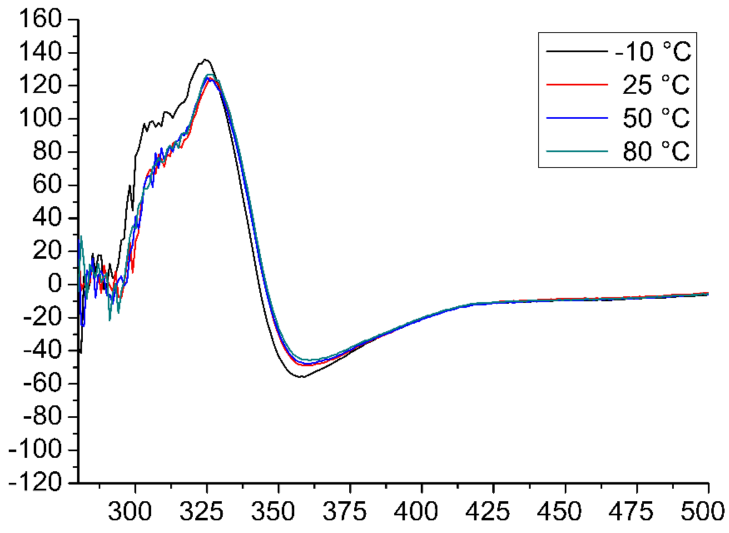

***Figure S42*.** CD spectra of a **Poly-10mer** recorded in toluene at different temperatures, concentration 100 mg/L. **Poly-10mer** was dissolved in 2-3 drops of chloroform then diluted with the required amount of toluene at room temperature.

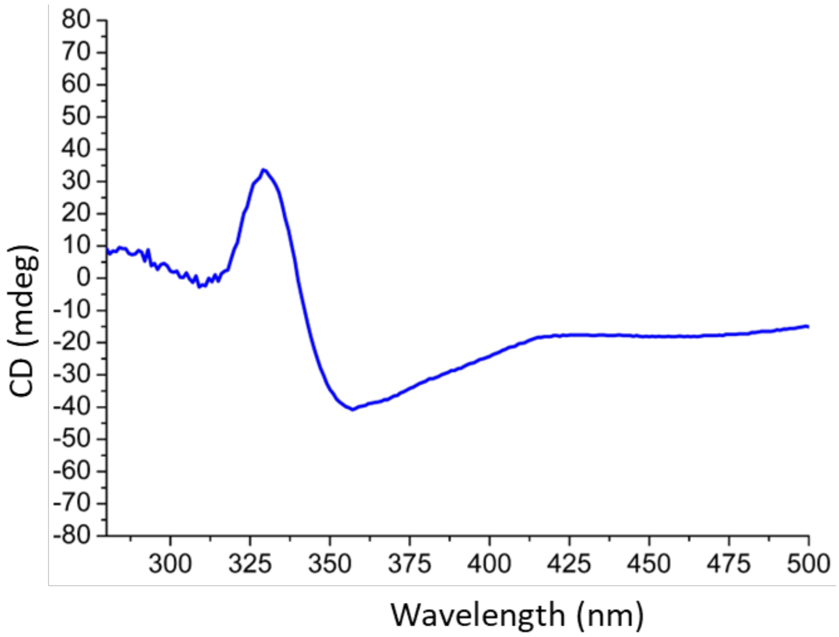

Figure S43. CD spectra of a **Poly-10mer** recorded in CCl_4 at 298 K, concentration 100 mg/L. **Poly-10mer** was dissolved in 2-3 drops of chloroform then diluted with the required amount of CCl_4 at room temperature.

DLS measurements

The hydrodynamic diameter and polydispersity index (PDI) of the samples were obtained by dynamic light scattering (DLS) measurements, which were performed with a NanoLab 3D system (LS Instruments AG, Fribourg, Switzerland). Measurements were carried out at 25 °C, at a scattering angle of 90° with a laser wavelength of 638 nm. To understand the effect of concentration and solvent type on particle size, samples were prepared at different concentrations by dissolving **Poly-10mer** was dissolved in 2-3 drops of chloroform and then adding required amount of toluene. Hydrodynamic diameter and PDI were calculated from the normalized autocorrelation function (ACF) via 2nd order cumulant analysis.[6] Acquisition time was set to 10 seconds and the presented diameters and PDIs are averages of 10 accumulations, with error bars representing a 95% confidence interval.

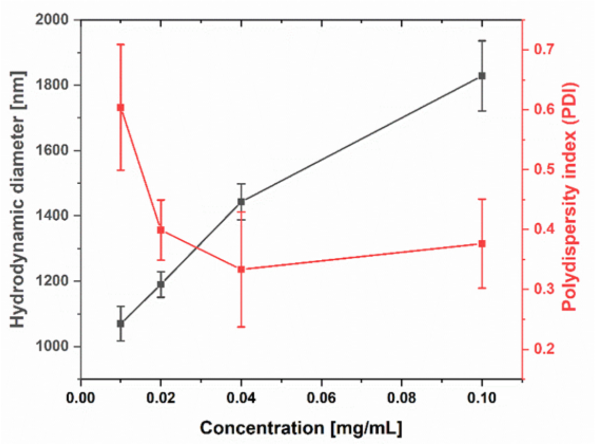

Figure S44. Hydrodynamic diameters and PDIs calculated from dynamic light scattering of **Poly-10mer** recorded in toluene. Concentrations: 0.01 mg/mL, 0.02 mg/mL, 0.04 mg/mL, and 0.1 mg/mL. **Poly-10mer** was dissolved in 2-3 drops of chloroform then diluted with the required amount of toluene at room temperature.

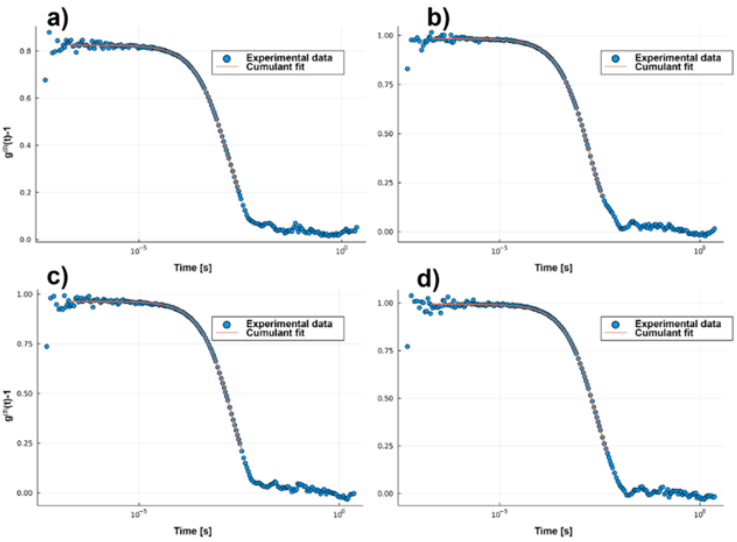

Figure S45. Autocorrelation curves obtained via DLS and their respective cumulant fits for **Poly-10mer** in toluene at different concentrations: (a) 0.01 mg/mL, (b) 0.02 mg/mL, (c) 0.04 mg/mL, and (d) 0.1 mg/mL).

6. X-Ray single Crystal data

X-ray single crystal structure of **10**.

Table S2 Crystal data and structure refinement for 10.

Identification code	10
Empirical formula	$C_{20}H_{21}FN_2O_7$
Formula weight	420.39
Temperature/K	250(2)
Crystal system	monoclinic
Space group	$P2_1$
a/Å	17.8381(12)
b/Å	7.0138(3)
c/Å	17.5390(12)
α/°	90
β/°	109.931(5)
γ/°	90
Volume/Å^3	2062.9(2)
Z	4
ρ_{calc}g/cm^3	1.354
μ/mm^{-1}	0.928
F(000)	880.0
Crystal size/mm^3	0.24 × 0.12 × 0.04
Radiation	Cu Kα (λ = 1.54186)
2Θ range for data collection/°	10.732 to 136.42
Index ranges	-20 ≤ h ≤ 21, -6 ≤ k ≤ 8, -20 ≤ l ≤ 20
Reflections collected	44171
Independent reflections	5362 [R_{int} = 0.0369, R_{sigma} = 0.0293]
Data/restraints/parameters	5362/37/590
Goodness-of-fit on F^2	1.031
Final R indexes [I>=2σ (I)]	R_1 = 0.0613, wR_2 = 0.1790
Final R indexes [all data]	R_1 = 0.0714, wR_2 = 0.2111
Largest diff. peak/hole / e Å^{-3}	0.50/-0.23
Flack parameter	0.4(4)

Table S3 Hydrogen Bonds for 10.

D	H	A	d(D-H)/Å	d(H-A)/Å	d(D-A)/Å	D-H-A/°
O7	H7	O13	0.83	1.82	2.648(5)	175.4
N2	H2	O1	0.91	1.98	2.714(4)	137.1
C9	H9	O5	0.94	2.25	2.853(6)	120.9
O14	H14	O6	0.83	1.81	2.637(5)	175.1
N4	H4A	O8	0.77	2.07	2.713(4)	140.

Experimental

Single crystals of $C_{20}H_{21}FN_2O_7$ **[10]** were crystallization by slow evaporation of DCM. A suitable crystal was selected and mounted on loop with oil on a STOE STADIVARI diffractometer. The crystal was kept at 250(2) K during data collection. Using Olex2[7], the structure was solved with the SHELXT[8] structure solution program using Intrinsic Phasing and refined with the SHELXL[9] refinement package using Least Squares minimisation.

Crystal structure determination of [10]

Crystal Data for $C_{20}H_{21}FN_2O_7$ (M =420.39 g/mol): monoclinic, space group P2$_1$ (no. 4), a = 17.8381(12) Å, b = 7.0138(3) Å, c = 17.5390(12) Å, β = 109.931(5)°, V = 2062.9(2) Å^3, Z = 4, T = 250(2) K, μ(Cu Kα) = 0.928 mm^{-1}, $Dcalc$ = 1.354 g/cm^3, 44171 reflections measured (10.732° ≤ 2Θ ≤ 136.42°), 5362 unique (R_{int} = 0.0369, R_{sigma} = 0.0293) which were used in all calculations. The final R_1 was 0.0613 (I > 2σ(I)) and wR_2 was 0.2111 (all data).

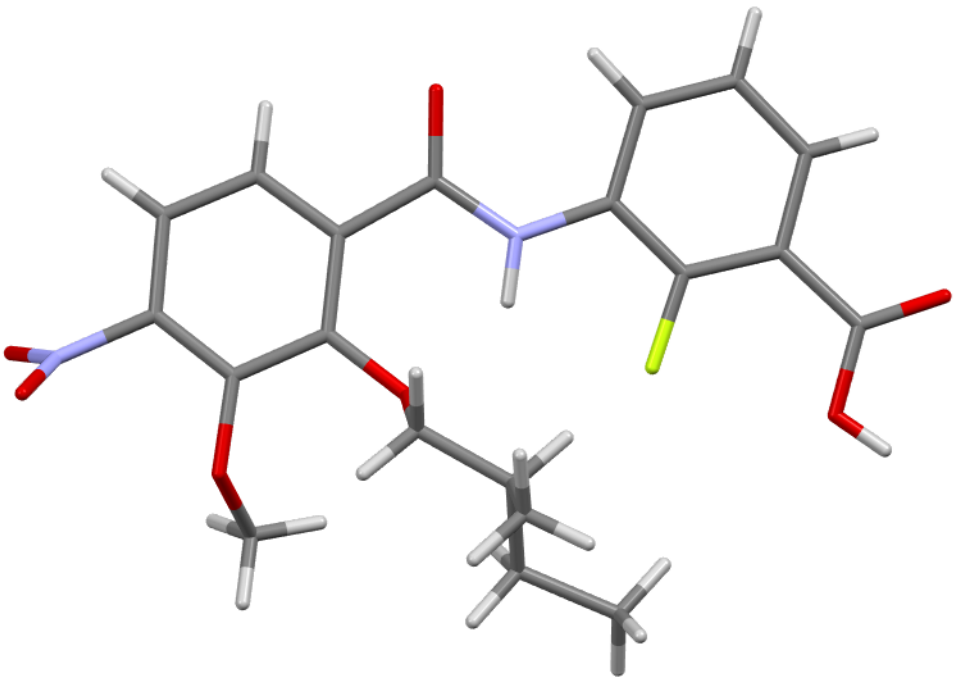

Figure S46. X-ray crystal structure of **10**.

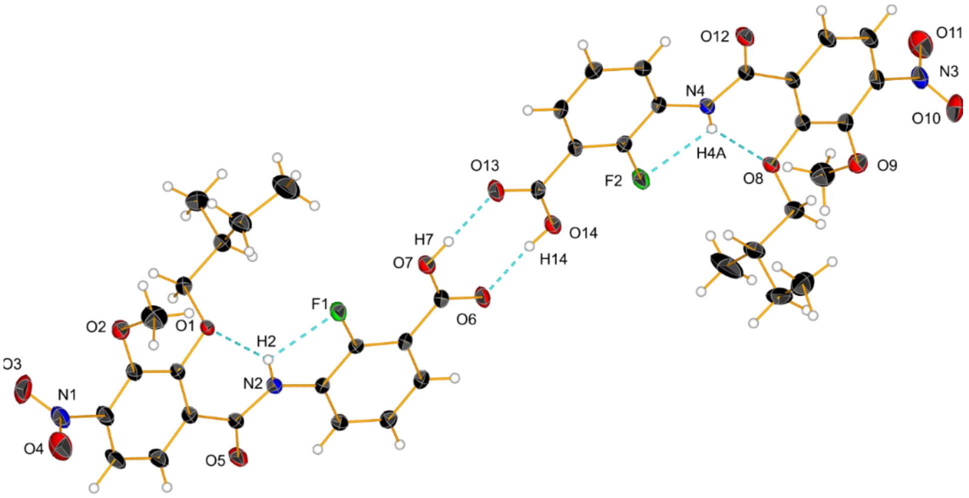

Figure S47. The ORTEP plot of crystal structure **10**.

X-ray single Crystal structure of **11**.

Table S4 Crystal data and structure refinement for 11.

Identification code	11
Empirical formula	$C_{80}H_{83}F_4N_8O_{22}$
Formula weight	1584.54
Temperature/K	250
Crystal system	triclinic
Space group	P-1
a/Å	11.5764(2)
b/Å	14.8804(2)
c/Å	23.1221(3)
α/°	93.7290(10)
β/°	99.4930(10)
γ/°	95.0890(10)
Volume/Å^3	3900.13(10)
Z	2
ρ_{calc}g/cm^3	1.349
μ/mm^{-1}	0.888
F(000)	1662.0
Crystal size/mm^3	0.16 × 0.04 × 0.02
Radiation	Cu Kα (λ = 1.54186)
2Θ range for data collection/°	7.396 to 136.546
Index ranges	-13 ≤ h ≤ 10, -17 ≤ k ≤ 17, -26 ≤ l ≤ 27
Reflections collected	111021
Independent reflections	13834 [R_{int} = 0.0770, R_{sigma} = 0.0347]
Data/restraints/parameters	13834/1/1001
Goodness-of-fit on F^2	1.040
Final R indexes [I>=2σ (I)]	R_1 = 0.1018, wR_2 = 0.2719
Final R indexes [all data]	R_1 = 0.1126, wR_2 = 0.2801
Largest diff. peak/hole / e Å^{-3}	0.75/-0.76

Table S5 Hydrogen Bonds for 11.

D	H	A	d(D-H)/Å	d(H-A)/Å	d(D-A)/Å	D-H-A/°
O2	H2	O1[1]	0.83	1.80	2.618(5)	169.1
N1	H1	F1	0.87	2.28	2.640(5)	105.2
N1	H1	O4	0.87	2.00	2.712(5)	138.1
N2	H2A	F2	0.87	2.02	2.736(4)	138.3
N2	H2A	O5	0.87	2.27	2.664(5)	107.2
N3	H3	F2	0.87	2.25	2.633(4)	106.6
N3	H3	O8	0.87	2.12	2.786(5)	132.7
O12	H12	O13[2]	0.83	1.81	2.596(5)	157.6
N5	H5A	F3	0.87	2.26	2.631(4)	105.3
N5	H5A	O15	0.87	2.00	2.708(5)	137.9
N6	H6A	F4	0.87	1.99	2.707(4)	139.4
N6	H6A	O16	0.87	2.22	2.645(5)	109.5
N7	H7A	F4	0.87	2.23	2.616(4)	107.1
N7	H7A	O19	0.87	2.02	2.746(5)	140.3

Experimental

Single crystals of $C_{80}H_{83}F_4N_8O_{22}$ **[11]** were crystallized from slow evaporation of chloroform. A suitable crystal was selected and mounted on loop with oil on a STADIVARI_LANA_250K diffractometer. The crystal was kept at 250 K during data collection. Using Olex2[7] , the structure was solved with the SHELXT[8] structure solution program using Intrinsic Phasing and refined with the SHELXL[9] refinement package using Least Squares minimisation.

Crystal structure determination of [11]

Crystal Data for $C_{80}H_{83}F_4N_8O_{22}$ (M =1584.54 g/mol): triclinic, space group P-1 (no. 2), a = 11.5764(2) Å, b = 14.8804(2) Å, c = 23.1221(3) Å, α = 93.7290(10)°, β = 99.4930(10)°, γ = 95.0890(10)°, V = 3900.13(10) Å^3, Z = 2, T = 250 K, μ(Cu Kα) = 0.888 mm^{-1}, $Dcalc$ = 1.349 g/cm^3, 111021 reflections measured (7.396° ≤ 2Θ ≤ 136.546°), 13834 unique (R_{int} = 0.0770, R_{sigma} = 0.0347) which were used in all calculations. The final R_1 was 0.1018 (I > 2σ(I)) and wR_2 was 0.2801 (all data) .

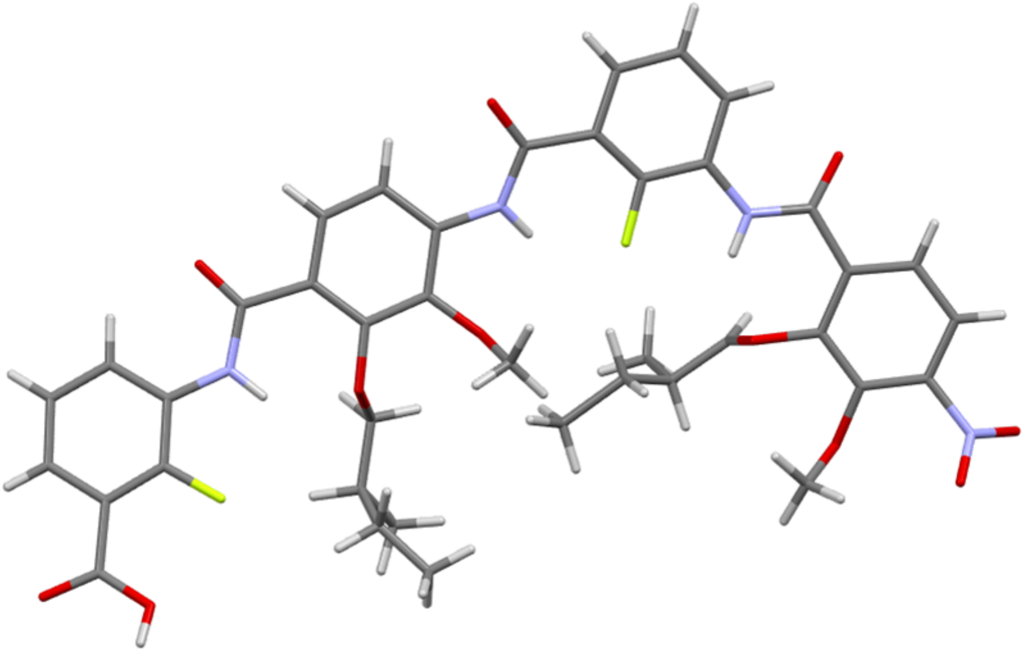

Figure S48. X-ray crystal structure of **11**.

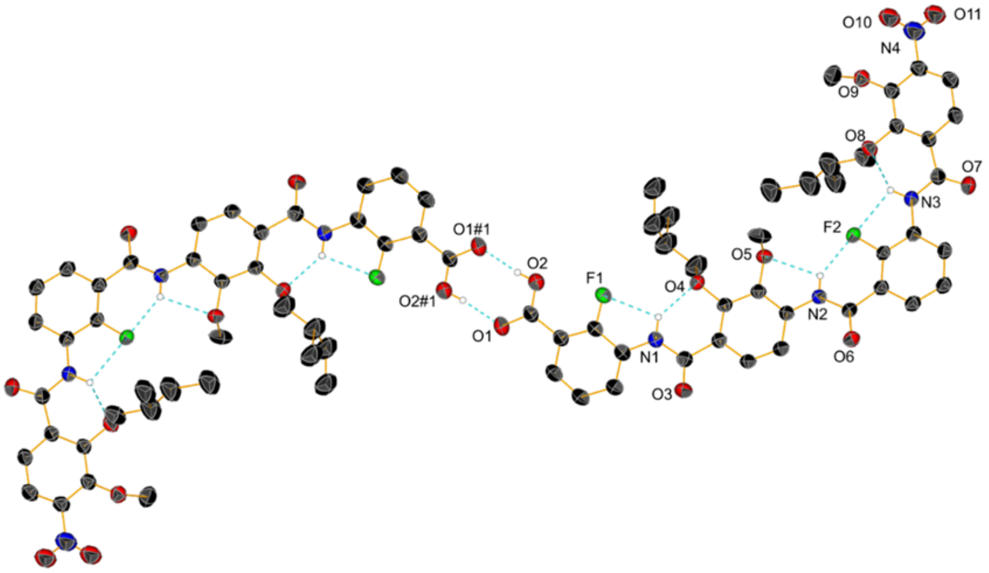

Figure S49. The ORTEP plot of crystal structure **11**.

X-ray single crystal structure of **4**.

***R_1* = 8.59%**

Crystal Data and Experimental

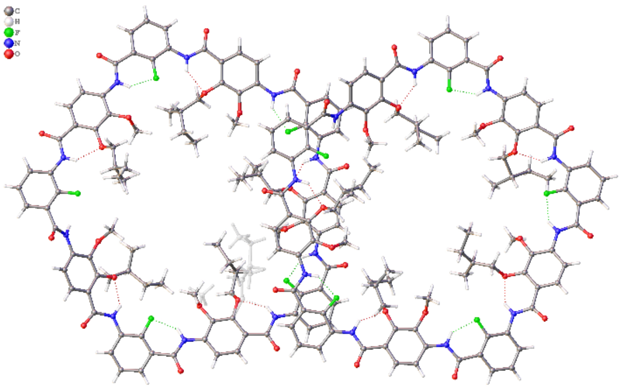

Experimental. Single clear pale colourless prism-shaped crystals of **4** were used as supplied. A suitable crystal with dimensions 0.28 × 0.23 × 0.16 mm³ was selected and mounted on a XtaLAB Synergy R, DW system, HyPix-Arc 150 diffractometer. The crystal was kept at a steady T = 140.00(10) K during data collection. The structure was solved with the **ShelXT** (Sheldrick, 2015) solution program using dual methods and by using **Olex2** 1.5 (Dolomanov et al., 2009) as the graphical interface. The model was refined with **ShelXL** 2018/3 (Sheldrick, 2015) using full matrix least squares minimisation on $\boldsymbol{F^2}$.

Crystal Data. $C_{100}H_{105}F_5N_{10}O_{20}$, M_r = 1861.93, monoclinic, $P2_1$ (No. 4), a = 7.41791(16) Å, b = 39.2528(5) Å, c = 34.5952(5) Å, β = 90.1072(16)°, α = γ = 90°, V = 10073.2(3) Å³, T = 140.00(10) K, Z = 4, Z' = 2, μ(Cu K_α) = 0.769, 158019 reflections measured, 32549 unique (R_{int} = 0.0513) which were used in all calculations. The final wR_2 was 0.2785 (all data) and R_1 was 0.0859 (I≥2 σ(I)).

**Compound**	**4**
Formula	$C_{100}H_{105}F_5N_{10}O_{20}$
$D_{calc.}$/ g cm^{-3}	1.228
μ/mm^{-1}	0.769
Formula Weight	1861.93
Colour	clear pale colourless
Shape	prism-shaped
Size/mm³	0.28×0.23×0.16
T/K	140.00(10)
Crystal System	monoclinic
Flack Parameter	0.1(2)
Hooft Parameter	-0.12(5)
Space Group	$P2_1$
a/Å	7.41791(16)
b/Å	39.2528(5)
c/Å	34.5952(5)
α/°	90
β/°	90.1072(16)
γ/°	90
V/Å³	10073.2(3)
Z	4
Z'	2
Wavelength/Å	1.54184
Radiation type	Cu K_α
Θ_{min}/°	2.251
Θ_{max}/°	66.600
Measured Refl's.	158019
Indep't Refl's	32549
Refl's I≥2 σ(I)	23574
R_{int}	0.0513
Parameters	2527
Restraints	2472
Largest Peak	1.231
Deepest Hole	-0.481
GooF	1.036
wR_2 (all data)	0.2785
wR_2	0.2484
R_1 (all data)	0.1126
R_1	0.0859

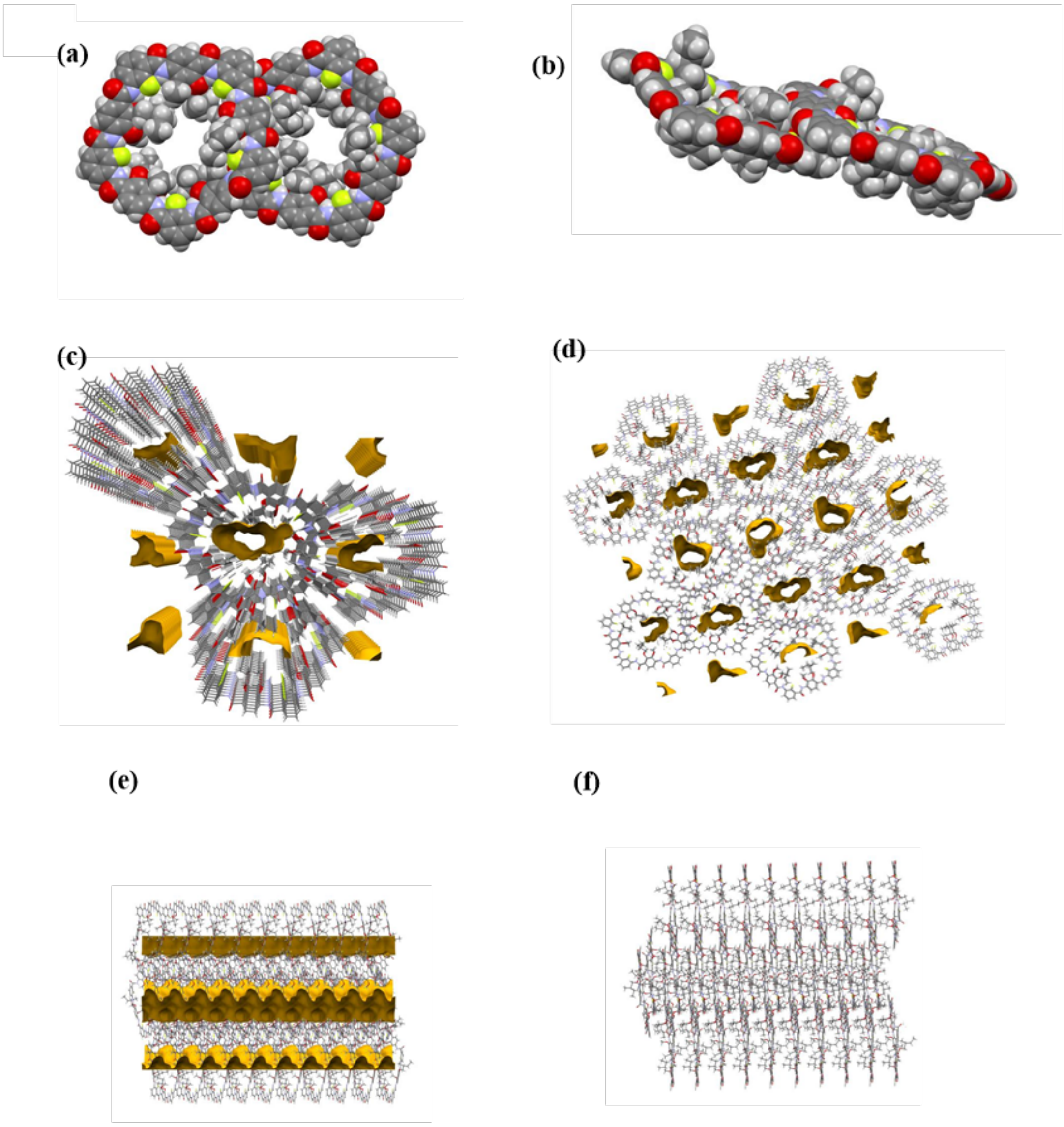

Figure S50. Space filling representations of the X-ray structure of **4**, (a) top view and (b) side view, (c & d) 2D molecular packing along axis *a* with available voids (yellow), (e) 2D molecular packing along axis *b*, with available voids (yellow), (e) 2D molecular packing along axis *b* without voids.

Structure Quality Indicators

**Reflections:**	d min (Cu\a) 2Θ=133.2° 0.84	I/σ(I) 21.4	Rint 5.13%	Full 133.2° 99.9
**Refinement:**	Shift -0.001	Max Peak 1.2	Min Peak -0.5	GooF 1.036

A clear pale colourless prism-shaped crystal with dimensions 0.28 × 0.23 × 0.16 mm^3 was mounted. Data were collected using a XtaLAB Synergy R, DW system, HyPix-Arc 150 diffractometer operating at T = 140.00(10) K.

Data were measured using ω scans with Cu K_α radiation. The diffraction pattern was indexed and the total number of runs and images was based on the strategy calculation from the program CrysAlisPro 1.171.42.72a (Rigaku OD, 2022). The maximum resolution that was achieved was Θ = 66D.600° (0.84 Å). The unit cell was refined using CrysAlisPro 1.171.42.72a (Rigaku OD, 2022) on 46009 reflections, 29% of the observed reflections.

Data reduction, scaling and absorption corrections were performed using CrysAlisPro 1.171.42.72a (Rigaku OD, 2022). The final completeness is 99.90 % out to 66.600° in Θ. A gaussian absorption correction was performed using CrysAlisPro 1.171.42.72a (Rigaku Oxford Diffraction, 2022). The numerical absorption correction was based on gaussian integration over a multifaceted crystal model. The empirical absorption correction was done using spherical harmonics, implemented in SCALE3 ABSPACK scaling algorithm. The absorption coefficient μ of this crystal is 0.769 mm^{-1} at this wavelength (λ = 1.54184Å) and the minimum and maximum transmissions are 0.404 and 1.000.
The structure was solved and the space group $P2_1$ (# 4) determined by the ShelXT (Sheldrick, 2015) structure solution program using dual methods and refined by full matrix least squares minimisation on F^2 using version 2018/3 of **ShelXL** (Sheldrick, 2015). All non-hydrogen atoms were refined anisotropically. Hydrogen atom positions were calculated geometrically and refined using the riding model.
_refine_special_details: Refined as a 2-component inversion twin.
_smtbx_masks_special_details: A solvent mask was calculated and 322 electrons were found in a volume of 1362Å^3 in 5 voids per unit cell. This is consistent with the presence of 1.5[DMA], 1.5[DMA] per Asymmetric Unit which account for 324 electrons per unit cell.
The value of Z' is 2. This means that there are two independent molecules in the asymmetric unit.
The Flack parameter was refined to 0.1(2). Determination of absolute structure using Bayesian statistics on Bijvoet differences using the Olex2 results in -0.12(5). Note: The Flack parameter is used to determine the chirality of the crystal studied, the value should be near 0, a value of 1 means that the stereochemistry is wrong and the model should be inverted. A value of 0.5 means that the crystal consists of a racemic mixture of the two enantiomers.

7. AFM studies

AFM analysis of macrocycle **4** was measured at a concentration of 4 mg/L. A stock solution of macrocycles in $CHCl_3$ was prepared and then diluted with $CHCl_3$ (HPLC grade).

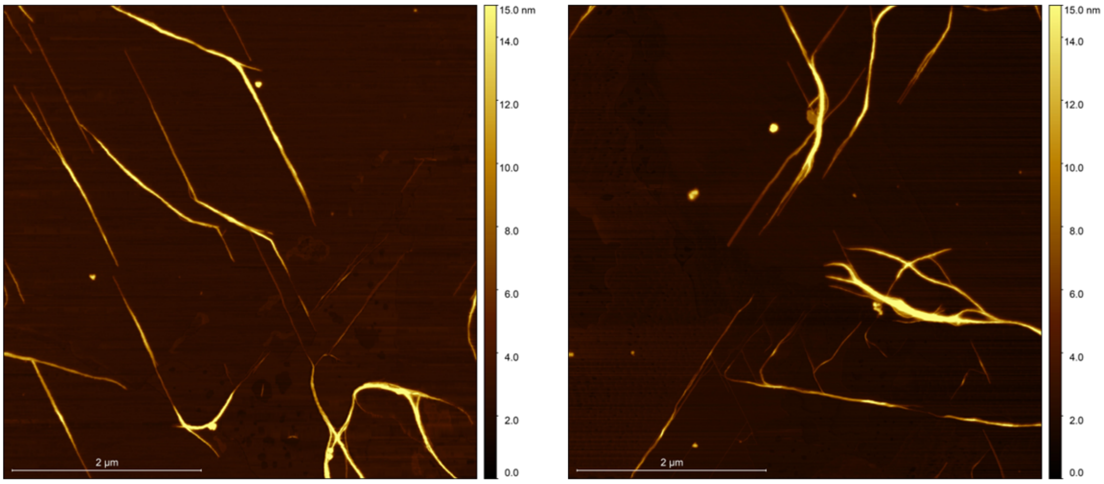

Figure S51. High-resolution AFM image of **4** with a scale bar 2 µm. Concentration (4 mg/L, Chloroform).

8. HRMS studies

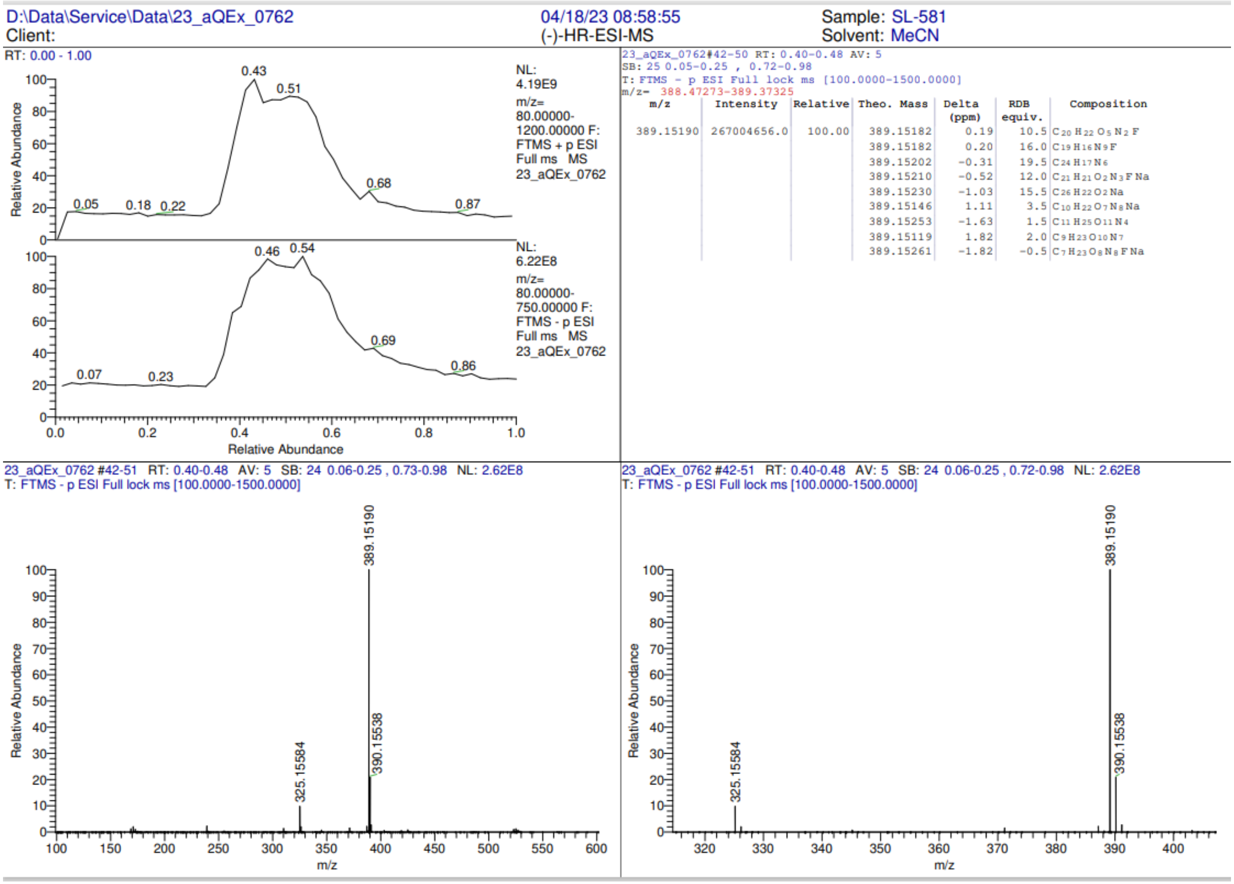

m/z	Intensity	Relative	Theo. Mass	Delta (ppm)	RDB equiv.	Composition
389.15190	267004656.0	100.00	389.15182	0.19	10.5	$C_{20}H_{22}O_5N_2F$
			389.15182	0.20	16.0	$C_{19}H_{16}N_9F$
			389.15202	-0.31	19.5	$C_{24}H_{17}N_6$
			389.15210	-0.52	12.0	$C_{21}H_{21}O_2N_3FNa$
			389.15230	-1.03	15.5	$C_{26}H_{22}O_2Na$
			389.15146	1.11	3.5	$C_{10}H_{22}O_7N_8Na$
			389.15253	-1.63	1.5	$C_{11}H_{25}O_{11}N_4$
			389.15119	1.82	2.0	$C_9H_{23}O_{10}N_7$
			389.15261	-1.82	-0.5	$C_7H_{23}O_8N_8FNa$

Figure S52. HRMS spectrum of **monomer 2**.

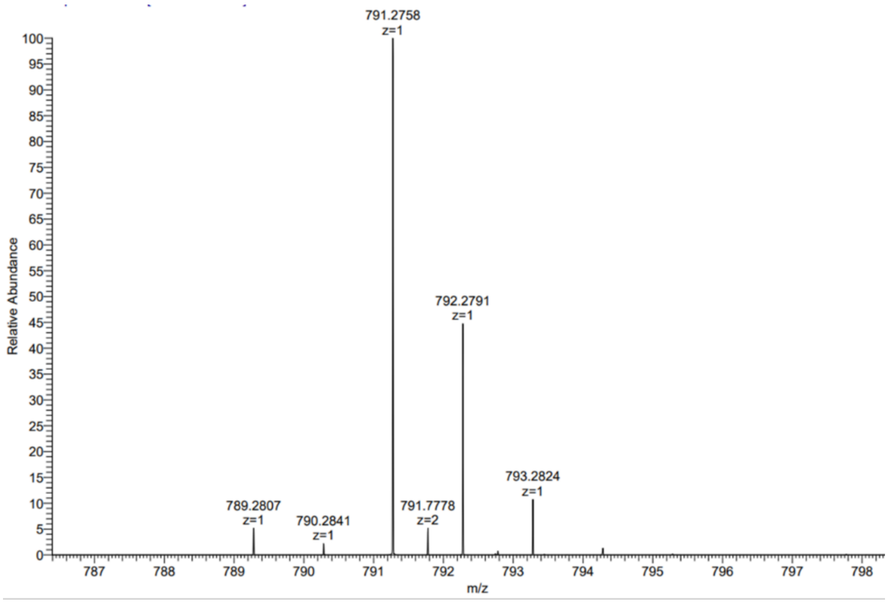

Figure S53. HRMS spectrum of **dimer 11**.

Guinier fit of SAXS data

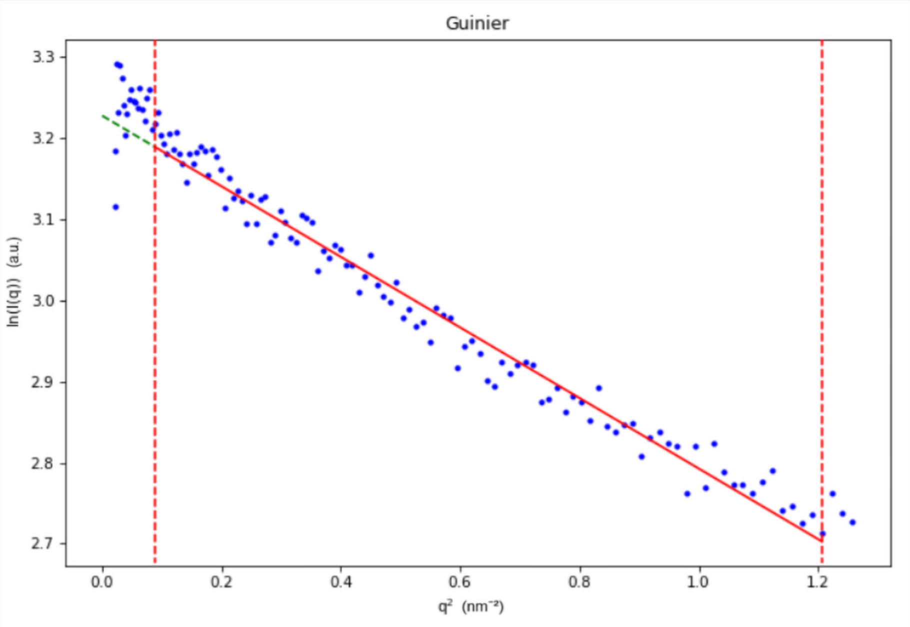

Figure S54. Guinier fit of SAXS curve of **Poly-10mer** in toluene/DMSO 9:1 v/v. R_g (1.1 nm) was extracted from the slope with Eq. S1.

9. Water Transport Experiments

Stopped-flow experiments for Water transport activity:[S11-S13]

HEPES buffer preparation for experiments: The required buffer consisting of 100 mM of NaCl and 10 mM of HEPES in Milli Q water and adjusted to pH = 7.0 by adding the required volume of NaOH (0.5 M) in water.

Vesicles preparation for permeability assay:

First, 0.25 mL of DOPC lipid solution (25 mg/mL in chloroform) was taken in a 10 mL round-bottomed flask, and required amount of channel-forming molecule (mol% compound with respect to lipid) in chloroform was added. The chloroform in the compound lipid solution was then evaporated by a slow stream of nitrogen gas while slowly rotating the round-bottomed flask to get a thin film of lipid inside it. Then the trace amount of chloroform present in the compound-lipid was evaporated by drying it in a high vacuum for about 12 h. The thin film of lipid was hydrated with buffer solution (10 mM HEPES, 100 mM NaCl, pH 7.0), and stirred for 1 h. Then the hydrated vesicles suspension was subjected to 15 freeze-thaw cycles (liquid nitrogen and 55 °C temperature water bath) and extruded 19 times (must be an odd number) using 200 nm polycarbonate membrane. The extruded vesicles were then diluted to 6 mL using the buffer above (10 mM HEPES, 100 mM NaCl, pH 7.0) to get the desired concentration (mol% of compound with respect to lipid), assuming no loss of lipid throughout the process. Vesicle composition: inside - 10 mM HEPES, 100 mM NaCl, pH 7.0 and outside - 10 mM HEPES, 100 mM NaCl, pH 7.0.

Description of stopped-flow experiments for water transport studies:

The above-mentioned vesicle solution was exposed to an equal volume of 300 mM sucrose solution in buffer (300 mM Sucrose, 10 mM HEPES, 100 mM NaCl, pH 7.0), which leads to the shrinkage of the vesicles due to the outwardly directed water transport through any water channels. The sudden shrinkage of the size of the vesicle changes the observed light scattering intensity with time (90˚ angle recorded at 600 nm wavelength), which was monitored on a stopped-flow instrument (KinetAsyst SF-61DX2 from TgK Scientific). The light scattering intensity was plotted against time by fitting the following exponential decay equation (Eq. S2).

$$y = A.exp(-kx) + y_0 \qquad \text{Eq. S2}$$

where, x is the time, y is the light scattering intensity change, and k is the exponential coefficient for the light scattering change.

This exponential coefficient value, k was used to calculate the osmotic permeability value, P_f, following the below equation:

$$Pf = \frac{k}{\frac{S}{Vo} * Vw * \Delta osm} \qquad \text{Eq. S3}$$

where, P_f is the osmotic permeability, k is the exponential coefficient, S is the initial surface area of the vesicles, V_0 is the initial volume of the vesicles, V_w is the molar volume of water, Δosm is the change in osmolarity of the vesicular suspension after the addition of sucrose solution.

The corrected water permeability, P_w, by the water channel molecules were calculated by subtracting the exponential coefficient value a compound from the exponential coefficient value of the blank data from the following equation:

$$P_w = (P_f(compound) - P_f(blank)) \times S/N \qquad \text{Eq. S4}$$

where, P_w is the corrected water permeability, P_f (compound) is the osmotic permeability of the compounds, P_f (blank) is the osmotic permeability of the blank vesicles, S is the surface area of the vesicles, and N is the number of channels per vesicles and can be calculated using the Eq. S5.

$$N = \frac{SA_{total}}{SA_{unit}} = \frac{2\pi r^2 + 2\pi(r-5)^2}{A_{channel} + \left(\frac{1-x}{x}\right) \times 0.34} \qquad \text{Eq. S5}$$

where, SA_{total} is the total surface area of a vesicles; SA_{unit} is the 'unit area'; $A_{channel}$ is the cross-sectional area of a single channel aggregate; x is the corrected mCLR. The number of single channels per liposome can then be used to calculate the single channel permeability (P_w) following Eq. S4.

The water permeability values were further modified using the recently reported equation S6 which accounts for the intra- and extra-vesicular osmolyte concentrations correction factor.

$$P_{f(corrected)} = \frac{k}{\frac{S}{V_0} \times V_w} \times \frac{C_{in,t=0} + C_{out}}{2 \times {C_{out}}^2} \qquad \text{Eq. S6}$$

where, $C_{in,\ t=0}$ is the change in osmolyte concentration inside the vesicles at t = 0 and C_{out} is the osmolyte concentration outside the vesicles.

The $P_{f(corrected)}$ values obtained from Eq. S6, considering the osmolyte concentration correction factor, were further used to calculate the corrected water permeability and single channel permeability for all channel forming molecules following the Eq. S4 and S4.

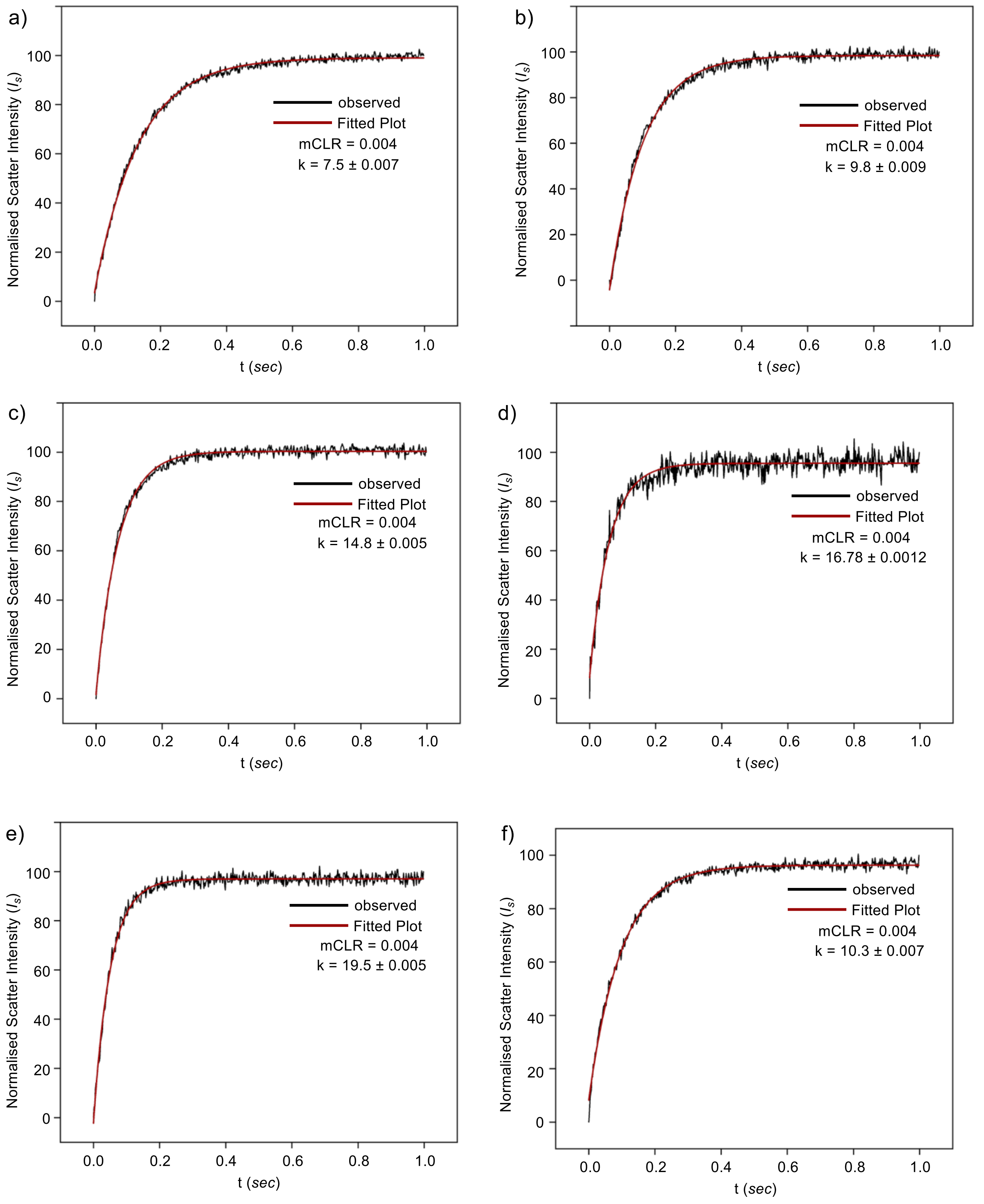

Figure S55. Kinetic curves for water permeability measurement. a) Blank, b) **3**, c) **4**, d) **Poly-10mer**, e) **Poly-20mer**, and f) **Poly-30mer**.

10. Ion Transport studies

Cation transport studies in HPTS assay:[S11-S13]

HEPES buffer, HPTS solution, and stock solution preparation for assay:

The buffer solution was prepared using milliQ water consisting of 10 mM HEPES (pH 7.0) and this buffer was used to prepare a salt solution of strength 200 mM of M_2SO_4 (M^+ = Na^+ and K^+). The pH was adjusted to 7 using the corresponding MOH (0.5 Molar, M^+ = Na^+ or K^+) solution. Then HPTS solution of 1 mM was prepared from solid HPTS using the above-mentioned HEPES buffer solution (without salt). The compound studied was preincubated in all the experiments.

Vesicles preparation for cation transport assay:

Firstly, 0.5 mL of EYPC lipid solution (25 mg/mL in chloroform) and compound (in $CHCl_3$) was taken in a 10 mL round bottom flask. The chloroform present in the lipid solution was then evaporated by a slow stream of nitrogen gas while rotating the round-bottomed flask to form a thin film of lipid. Then the trace amount of chloroform present in the lipid was evaporated by drying it in a high vacuum for about 12 h. The thin film of lipid was hydrated with HPTS solution (1.0 mM HPTS, 10 mM HEPES, pH 7.0) while vortexing 4-5 times over the period of 1 h. Then the hydrated vesicle suspension was subjected to 15 freeze-thaw cycles and extruded 19 times (must be an odd number) using a 200 nm polycarbonate membrane. The extravesicular dye was separated from vesicles by size exclusion column chromatography (using Sephadex G-50 gel) eluting with buffer solution (10 mM HEPES, pH 7). After collecting, the vesicles from the column were diluted to 3 mL using buffer (10 mM HEPES, pH 7.0) to get a concentration of ~ 5.5 mM of EYPC-LUVs⊃HPTS, assuming no loss of lipid throughout the process. Vesicle composition: inside - 1 mM HPTS, 10 mM HEPES, pH 7.0 and outside - 10 mM HEPES, pH 7.0.

Description of cation transport study:

In a clean cuvette, 1975 µL of buffer solution (10 mM HEPES, 200 mM of M_2SO_4 (M^+ = Na^+ and K^+), pH 7.0), 25 µL of HPTS trapped vesicles solution (encapsulating compound) were taken and placed in the fluorometer equipped with a magnetic stirrer. The fluorescence emission intensity of the HPTS dye was measured at λ_{em} = 510 nm (where, λ_{ex} = 450 nm) for 350 s. For each reading, the start time of the instrument was considered as t = 0 s. Then at t = 100 s, 20 µL of 0.5 M NaOH solution was added to the same cuvette to generate a pH gradient (ΔpH = 0.8) between intra- and extravesicular medium. At t = 300 s, 10% triton X-100 (25 µL) was added to lyse all the vesicles to remove the pH gradient.

The fractional emission intensity (in percentage), I_F was calculated after normalizing all the data using the following equation (Eq. S7).

$$\% I_F = (I_t - I_0) / (I_\infty - I_0) \times 100 \qquad \text{Eq. S7}$$

Where I_0 is the initial fluorescence intensity *i.e.* before the addition of the transporter compound, I_t is the fluorescence intensity at time t and I_∞ is the final fluorescence intensity i.e. after the addition of Triton X-100.

The time axis was normalized using the following equation:

$$t = t - 100 \qquad \text{Eq. S8}$$

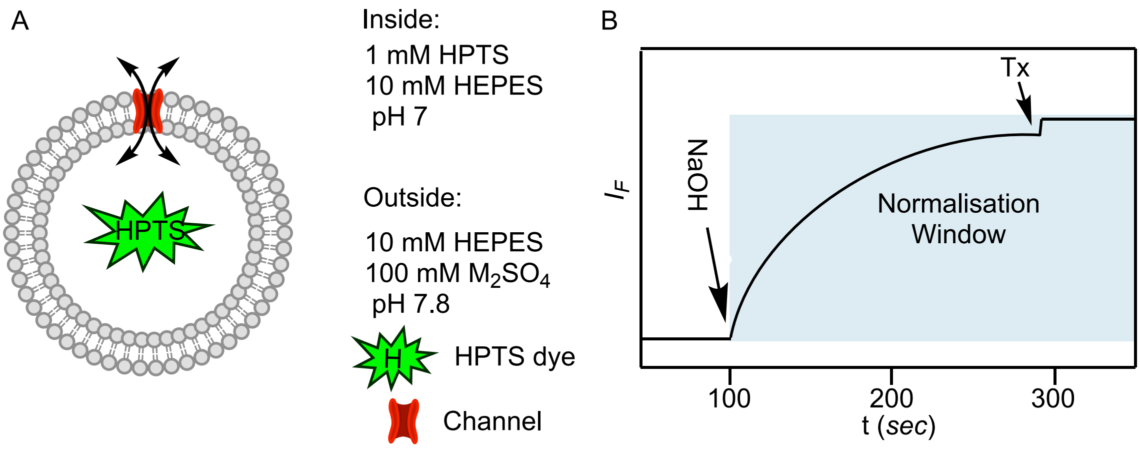

Figure S56. (A) Schematic representation of fluorescence kinetics assay for checking cation transport activity across EYPC-LUVs⊃HPTS and (B) normalisation working window for the same experiment.

Proton and anion transport studies in HPTS assay:[S11,S12,S13]

HEPES buffer, HPTS solution

The buffer solution was prepared using milliQ water consisting of 10 mM HEPES (pH 7.0) and this buffer was used to prepare a salt solution of strength 100 mM of NaCl and 66 mM of Na_2SO_4, followed by adjusting to pH 7.0 using NaOH (0.5 M). Then HPTS solution of 1.0 mM was prepared from solid HPTS using the above-mentioned HEPES buffer solution (100 mM of NaCl).

Vesicles preparation for proton and anion transport assay:

Firstly, 1 mL of EYPC lipid solution (25 mg/mL in chloroform) and compound (in $CHCl_3$) were taken in a 10 mL round bottom flask. The chloroform present in the lipid solution was then evaporated by a slow stream of nitrogen gas while rotating the round-bottomed flask to form a thin film of lipid. Then the trace amount of chloroform present in the lipid was evaporated by drying it in a high vacuum for about 12 h. The thin film of lipid was hydrated with HPTS solution (1.0 mM HPTS, 100 mM NaCl, 10 mM HEPES, pH 7.0) while vortexing 4-5 times over the period of 1 h. Then the hydrated vesicles suspension was subjected to 15 freeze-thaw cycles and extruded 19 times (must be an odd number) using a 200 nm polycarbonate membrane. The extravesicular dye was separated from vesicles by size exclusion column chromatography (using Sephadex G-50 gel) eluting with buffer solution (10 mM HEPES, 100 mM NaCl, pH 7.0). After collection, the vesicles from the column were diluted to 6 mL using the buffer as mentioned above (10 mM HEPES, 100 mM NaCl, pH 7.0) to get a concentration of ~ 5.5 mM of EYPC-LUVs⊃HPTS, assuming no loss of lipid throughout the process. Vesicle composition: inside - 1.0 mM HPTS, 10 mM HEPES, 100 mM NaCl, pH 7.0 and outside - 10 mM HEPES, 100 mM NaCl, pH 7.0.

Description of proton and anion transport study:

In a clean cuvette, 1975 μL of buffer solution (10 mM HEPES, 66 mM of Na_2SO_4, pH 7.0), 25 μL of vesicles entrapping HPTS and compound were and placed in a fluorescence instrument equipped with a magnetic stirrer. The fluorescence emission intensity of the HPTS dye, I_t was measured at λ_{em} = 510 nm (where λ_{ex} = 450 nm) for 350 s. For each reading, the start time of the instrument was considered as t = 0 s. Then at t = 100 s, 20 μL of 0.5 M NaOH solution was added to the same cuvette to generate a pH

gradient (ΔpH = 0.8) between intra- and extravesicular medium. At t = 300 s, 10% Triton X-100 (25 μL) was added to lyse all the vesicles to dissipate the pH gradient.

The fractional emission intensity (in percentage), I_F (Fig. S12B) was calculated after normalizing all the data using the following equation (Eq. S7).

$$\% I_F = (I_t - I_0) / (I_\infty - I_0) \times 100 \qquad \text{Eq. S7}$$

Where, I_0 is the initial fluorescence intensity i.e., before the addition of the transporter compound, I_t is the fluorescence intensity at time t, and I_∞ is the final fluorescence intensity i.e., after the addition of Triton X-100.

Again, the time axis was normalized using the following equation:

$$t = t - 100 \qquad \text{Eq. S8}$$

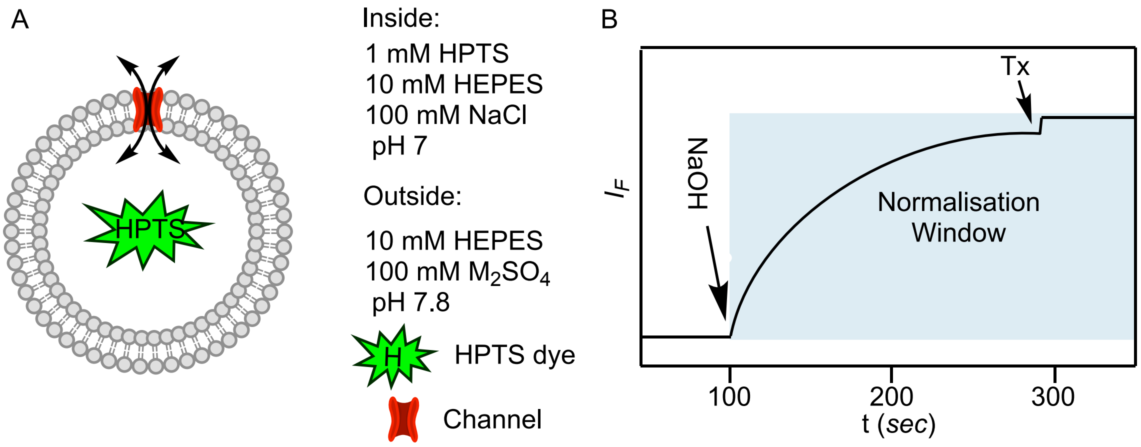

Figure S57. (A) Schematic representation of fluorescence kinetics assay for checking proton and anion transport activity across EYPC-LUVs⊃HPTS and (B) normalized working window for the same experiment.

Lucigenin assay for checking Cl^- transport activity

Salt and Stock solution preparation for Lucigenin assay:

225 mM $NaNO_3$ salt solution was prepared using milliQ water and phosphate buffer with pH adjusted to 7. Then 1.0 mM Lucigenin solution was prepared using this buffer. The compound under investigation was preincubated in lipids before liposome formation.

Vesicles preparation procedure for Lucigenin assay:

Firstly, 1.0 mL of EYPC lipid solution (25 mg/mL in chloroform) was taken in a 10 mL round bottom flask followed by addition of compound (in $CHCl_3$). The chloroform present in the solution was then evaporated by a slow stream of nitrogen gas while rotating the round-bottomed flask to form a thin film of lipid. Then the last trace amount of chloroform present in the lipid was evaporated by drying it in a high vacuum for about 12 h. The thin film of lipid was hydrated with 1 mL of Lucigenin solution (1.0 mM Lucigenin, 225 mM $NaNO_3$, 10 mM sodium phosphate buffer, pH 7) while vortexing 4-5 times for 2 min and stirred for 1 h. Then the hydrated vesicles suspension was subjected to 15 freeze-thaw cycles and extruded 19 times (must be an odd number) using a 200 nm polycarbonate membrane. The extravesicular dye was separated from vesicles by size exclusion column chromatography (using Sephadex G-50 gel) eluting

with $NaNO_3$ solution (225 mM). After collecting, the vesicles from the column were diluted to 4 mL using the buffer as mentioned above (225 mM $NaNO_3$) to get the concentration of ~5mM of EYPCLUVs⊃Lucigenin, assuming no loss of lipid throughout the process. Vesicle composition: inside - 1.0 mM Lucigenin, 225 mM $NaNO_3$ and outside - 225 mM $NaNO_3$.

Description of lucigenin assay:

In a clean cuvette, 1950 µL of salt solution (225 mM $NaNO_3$, phosphate buffer, pH 7), 50 µL of vesicles entrapping lucigenin and compound were taken and placed in a fluorescence instrument equipped with a magnetic stirrer. The fluorescence emission intensity of the lucigenin dye, I_t was measured at λ_{em} = 535 nm (where λ_{ex} = 450 nm) for 350 s. For each reading, the start time of the instrument was considered as t = 0 s. Then at t = 100 s, 33 µL of 2 N NaCl solution was added to the cuvette for generating a Cl^- concentration gradient across the lipid bilayer. At *t* = 300 s, 10% Triton X-100 (25 µL) was added to lyse all the vesicles to dissipate the Cl^- concentration gradient.

The fractional emission intensity (in percentage), I_F was calculated after normalizing all the data using the following equation (Eq. S9).

$$\% I_F = (I_t - I_0) / (I_\infty - I_0) \times (-100) \qquad \text{Eq. S9}$$

Where, I_0 is the initial fluorescence intensity i.e., before the addition of the transporter compound, I_t is the fluorescence intensity at time t, and I_∞ is the final fluorescence intensity i.e., after the addition of Triton X-100.

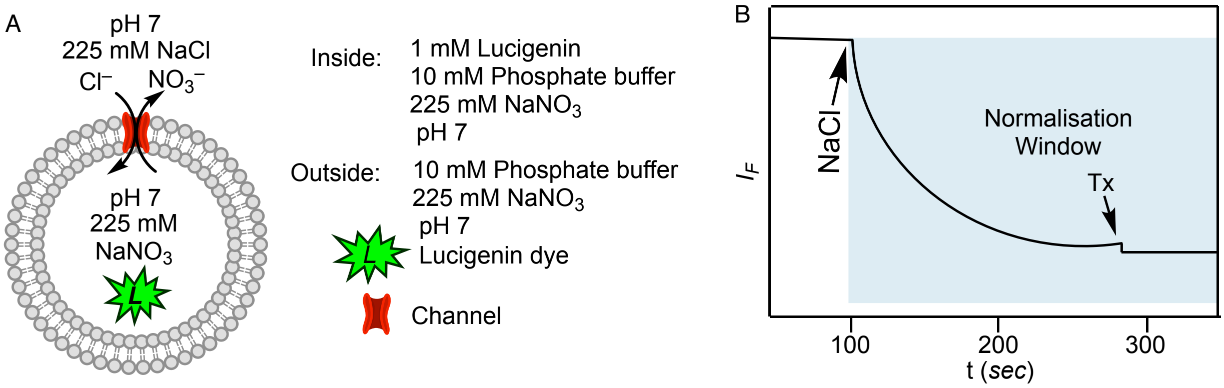

Figure S58. (A) Schematic representation of fluorescence kinetics assay for checking Cl− ion transport selectivity across EYPC-LUVs⊃Lucigenin and (B) normalized working window for the same experiment.

11. Molecular dynamics simulations

Based on the molecular dynamics simulation trajectories, the following analyses were conducted to evaluate the system's structural stability, compactness, and dynamic behavior. Additionally, the cavity diameter—a critical structural property—was examined to better understand the internal geometry of the polymer structures and their capacity to accommodate water molecules. In the context of molecular dynamics simulations, cavity diameter variations were analyzed over time using trajectory data to assess changes in size and structural adaptability.

Radius of gyration (Rg)

The radius of gyration (Rg) calculated from the molecular dynamics (MD) trajectory reflects the compactness and structural rigidity of the system throughout the simulation. Higher Rg values correspond to a more expanded structure, whereas lower Rg values indicate increased stability and compactness.

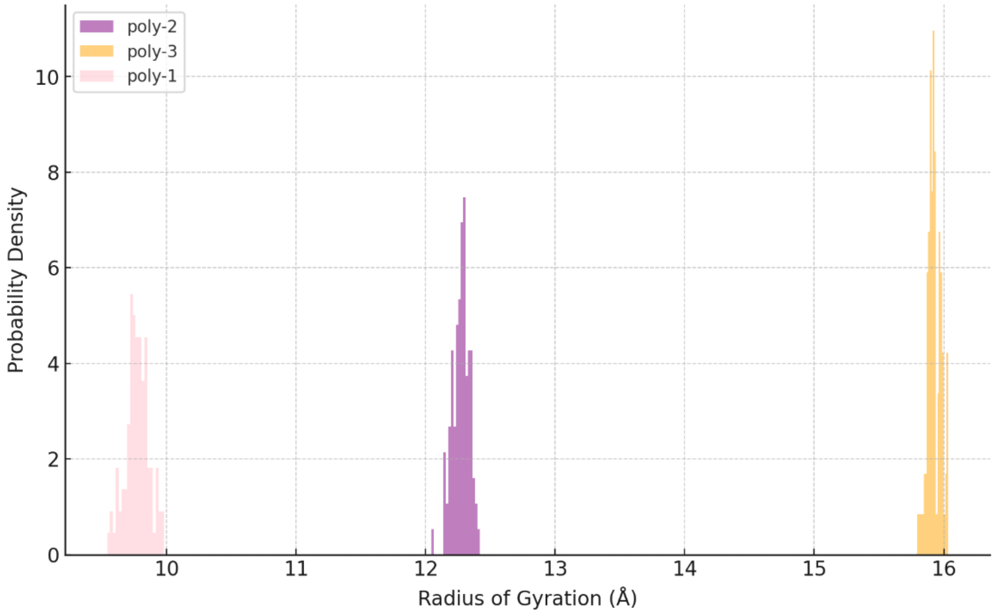

Figure S59. Probability distribution of radius of gyration for **Poly-10mer**, **Poly-20mer,** and **Poly-30mer**.

Cavity diameter

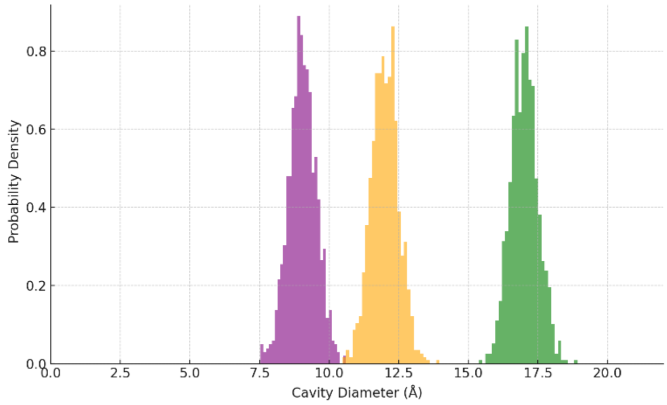

Structure	Cavity Diameter (Å)
Poly-10mer (purple)	8.9
Poly-20mer(green)	17.5
Poly-30mer (orange)	12.4

Figure S60. Probability distribution of cavity diameter for the **Poly-10mer**, **Poly-20mer**, and **Poly-30mer**.

The **Poly-10mer** exhibits the smallest cavity diameters, ranging from 8 to 10 Å, indicating more confined internal spaces. In contrast, the **Poly-20mer** has the largest cavity diameters, ranging from 16 to 18 Å, suggesting a more open internal structure. The **Poly-30mer** shows an intermediate cavity size distribution, centered around 12.5 Å, representing a balance between compactness and openness.

Root mean square fluctuation RMSF

RMSF is a measure of the flexibility or mobility of atoms in a molecular system throughout a simulation. It quantifies the average deviation of each atom's position from its reference position (e.g., the average or initial structure) during the trajectory.

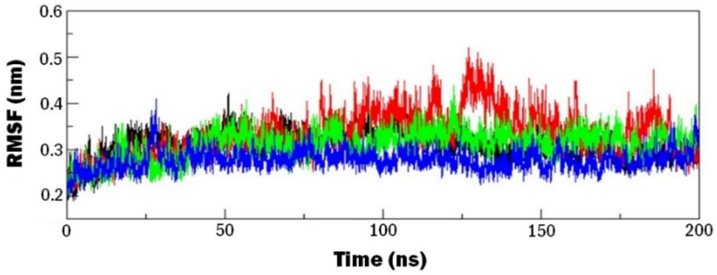

Figure S61. RMSF for **Poly-10mer** (blue), **Poly-20mer (**(green), and **Poly-30mer** (red).

Poly-10mer is the most compact and spherical structure, which aligns with its low asphericity. Low RMSF reflects high structural stability and minimal flexibility. The green trace corresponds to **Poly-20mer** (highest asphericity), showing significantly higher fluctuations than **Poly-10mer** but lower than **Poly-30mer**, where we have an additional factor, which is the length of the structure. Fluctuations appear more widespread across the structure, indicating a more flexible and dynamic system.

Key properties

Table S6: Collected data for each structure's mean pore size, cavity diameter, channel volume, and Rg.

Structure	Mean Pore Size (Å)	Cavity Diameter (Å)	Mean Channel Volume (Å³)	Rg (Å)
Poly-10mer	5.50	8.97	115.8	9.13
Poly-20mer	6.02	17.4	100.5	11.78
Poly-30mer	5.95	12.4	110.3	15.53

12. Previous reported aramide water channels

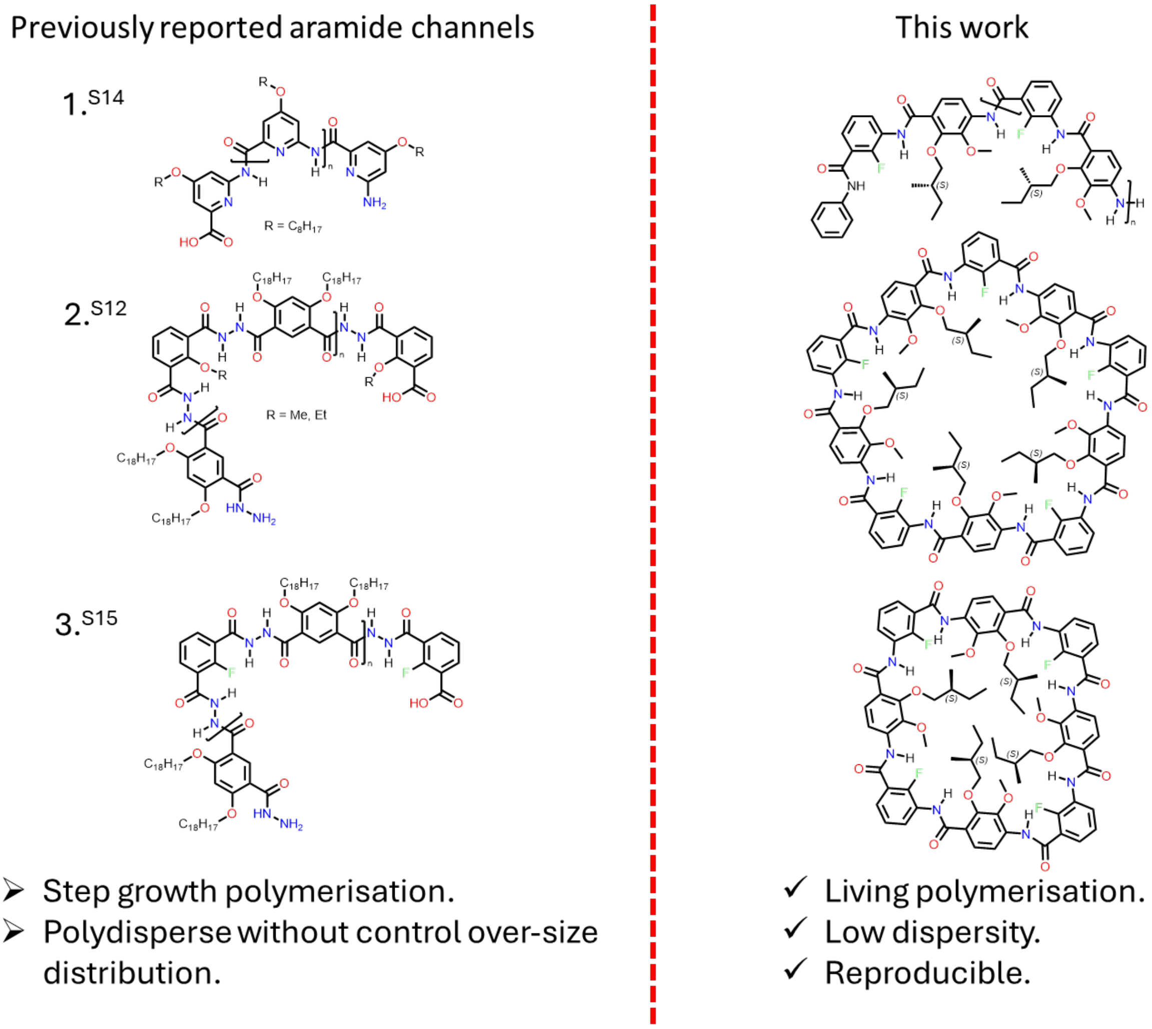

13. References

(S1) H. Berendsen, D. van der Spoel, R. van Drunen, *Comput. Phys. Commun.* **1995**, *91*, 43–56.

(S2) E. Lindahl, B. Hess, D. van der Spoel, *J. Mol. Model.* **2001**, *7*, 306–317.

(S3) B. Hess, C. Kutzner, D. van der Spoel, E. Lindahl, *J. Chem. Theory Comput.* **2008**, *4*, 435–447.

(S4) A. K. Malde, L. Zuo, M. Breeze, M. Stroet, D. Poger, P. C. Nair, C. Oostenbrink, A. E. Mark,*J. Chem. Theory Comput.* **2011**, *7*, 4026–4037.

(S5) S. Kishimoto, S. Nishimura, M. Hatano, M. Igarashi, H. Kakeya,*J. Org. Chem.* **2015**, *80*, 6076–6082.

(S6) B. J. Frisken, *Applied Optics,* **2001**, *40*, 4087–4091.

(S7) O. V. Dolomanov, L. J. Bourhis, R. J. Gildea, J. A. K. Howard, H. Puschmann, *J. Appl. Cryst*. **2009**, **42**, 339–341.

(S8) G. M. Sheldrick, *Acta Cryst*. **2015**, *A71*, 3–8.

(S9) G. M. Sheldrick, *Acta Cryst.* **2015**, C71, 3–8.

(S10) CrysAlisPro Software System, Oxford Diffraction, (**2022**).

(S11) Y. Leduc, M. Michau, A. Gilles, V. Gence, Y. M. Legrand, A. Vanderlee, S. Tingry, M. Barboiu, *Angew. Chem., Int. Ed.* **2011**, *50*, 11366–11372.

(S12) A. Roy, J. Shen, H. Joshi, W. Song, Y. M. Tu, R. Chowdhury, R. Ye, N. Li, C. Ren, M. Kumar, A. Aksimentiev, H. Zeng,*Nat. Nanotechnol*. **2021**, *16*, 911–917.

(S13) A. Mondal, D. Mondal, S. Sarkar, U. Shivpuje, J. Mondal, P. Talukdar, *Angew. Chem., Int. Ed*. **2025**, *64*, e202415510.

(S14) J. Shen, J. Fan, R. Ye, N. Li, Y. Mu, H. Zeng, *Angew. Chem., Int. Ed.* ,**2020**, *59*, 13328-13334.

(S15) J. Shen, A. Roy, H. Joshi, L. Samineni, R. Ye, Y. M. Tu, W. Song, M. Skiles, M. Kumar, A. Aksimentiev, H. Zeng, *Nano Lett.* **2022**, *22*, 4831–4838.
